# Material Diets for Climate-Neutral Construction

**DOI:** 10.1021/acs.est.1c05895

**Published:** 2022-04-04

**Authors:** Olga Beatrice Carcassi, Guillaume Habert, Laura Elisabetta Malighetti, Francesco Pittau

**Affiliations:** † Department of Architecture, Built Environment and Construction Engineering (ABC), Politecnico di Milano, Via G. Ponzio 31, 20133 Milan, Italy; ‡ Department of Civil, Environmental, and Geomatic Engineering, ETH Zurich, Stefano-Franscini-Platz 5, CH-8093 Zurich, Switzerland

**Keywords:** climate-neutral construction, embodied GHG, fast-growing biobased material, GWP_bio_, material GHG compensation

## Abstract

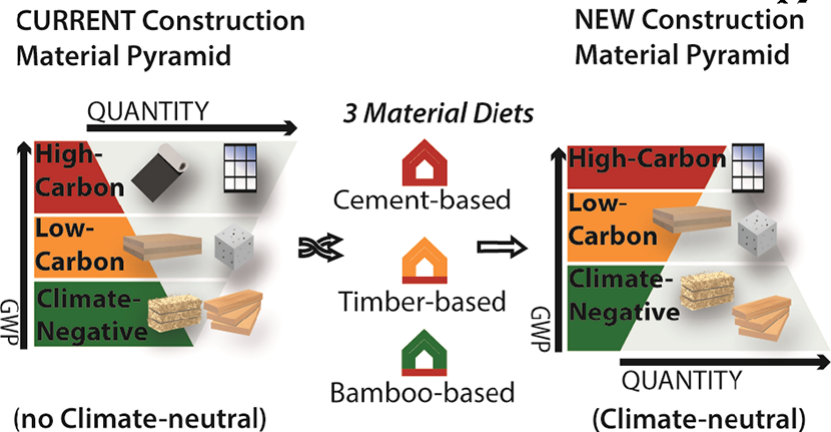

The climate crisis is urging us to act fast. Buildings are a key leverage point in reducing greenhouse gas (GHG) emissions, but the embodied emissions related to their construction often remain the hidden challenge of any ambitious policy. Therefore, in this paper, we explored material GHG neutralization where herbaceous biobased insulation materials with negative net-global warming potentials (GWPs) were used to compensate for building elements that necessarily release GHGs. Different material diets, as well as different building typologies, were modeled to assess the consequences in terms of biobased insulation requirements to reach climate neutrality. Our results show that climate-neutral construction can be built with sufficient energy performance to fulfill current standards and with building component thicknesses within a range of 1.05–0.58 m when timber- and bamboo-based construction is chosen. Concrete-based ones require insulation sizes that are too large and heavy to be supported by the dimensioned structures or accepted by urban regulations. Moreover, a time horizon of 20 years is more appropriate for assessing the contribution of material shifts to biobased materials in the transition period before 2050. This paper demonstrates that this is technically feasible and that climate neutrality in the construction sector just depends on the future that we choose.

## Abstract 2

This article explores the consequences in terms of material choices to reach climate neutrality at the construction scale. To reverse the current material pyramid, three material diets are designed: cement-based, timber-based, and bamboo-based. With these optimized material selection strategies, high- and low-carbon material emissions are compensated by the appropriate amount of climate-negative or herbaceous biobased materials to reach climate-neutral construction.

## 1 Introduction

The climate crisis is prompting an intensive examination into the reduction of anthropogenic greenhouse gas (GHG) emissions.^1^ Because the latest IPCC report highlighted that limiting warming to close to 1.5 °C or even 2 °C will be beyond reach without immediate, rapid, and large-scale reductions in GHG emissions,^2^ the question of budgets and orientations for future industries has become more stringent.^3^ The new Green Deal in the EU^4^ and many national climate-neutral initiatives have been engaged.^5,6^ Although current efforts are still clearly not in line with planetary boundaries,^1^ the objective of a net-zero emission target by mid-century is an accepted goal.

Buildings are clearly identified by policy makers as a key leverage point to reduce GHG emissions.^7^ Current research has traditionally focused on the use-phase emissions of buildings (called operational GHG emissions),^8,9^ while neglecting the emissions arising from the manufacturing and processing of building materials (called embodied GHG emissions).^10,11^ However, the embodied GHG emissions of energy-efficient buildings are approximately responsible for 45–50% of the total GHG emissions when a full life cycle is considered, unlike 20–25% of buildings that follow the current energy performance regulations.^12,13^ Evidently, a compromise between embodied and operational emissions exists.^14^ Nonetheless, when normalizing for the building service life and transforming the values to the combined sum of embodied and operational GHG emissions throughout the years of the life cycle,^12^ the operational emissions can be cut with the energy transition toward low-carbon alternatives and virtuous user behavior.^11^ In addition, the production and construction emissions are actuated in the early building life-cycle stage according to today’s energy mix and material production technologies without the possibility of being diminished.^11,15,16^ In fact, embodied emissions are manifested as a “carbon spike”, that is, a consistent amount of emissions occurring now in a short span of time,^17,18^ with the risk of consuming the remaining GHG budget that should be employed to manufacture low-carbon energy production plants and meet the climate neutrality target for 2050.^19,20^

### 1.1 Existing Climate-Neutral Strategies for Construction

Strategies for mitigating embodied construction emissions currently focus on the reduction of building construction and demolition waste,^21^ on the enhancement of material efficiency^9^ or by choosing alternative materials characterized by lower embodied emissions.^22^ Although these strategies could reduce the emissions for construction by 50%, they cannot stop releasing GHGs and, as a consequence, reach “absolute zero” emissions.^23^ For example, most buildings require cement for concrete foundations or structures, and complete decarbonization is not possible due to energy-intensive manufacturing processes and emissions related to calcination reactions.^24−26^ New frontiers for carbon-neutral concrete solutions have been explored^27,28^ but cannot cope with the scale of the construction boom due to future urbanization^29^ and the pace of decarbonization required to stay within planetary boundaries.^30^

Unlike absolute zero emissions, “net zero” implies the possibility of offsetting the remaining GHG emissions with carbon dioxide removal or “negative emission” strategies.^31^ Biobased materials fall into this category, as they are able to remove carbon dioxide through photosynthesis with the growth or regrowth of the plant once the biomass is harvested.^32,33^ Accordingly, the replacement of concrete by timber in construction is an interesting option, as it simultaneously reduces the emissions coming from concrete production and allows for the storage of carbon in the building stock. Buildings can then be considered a global carbon sink,^33^ but the question of resource availability limits the extent of a full transition from concrete to timber for structural materials.^35^ Depending on the local conditions, economic constraints, and resource availability, timber cannot be imposed everywhere in the world without the risk of reducing carbon sinks from forests, as has been recently observed in Europe.^36^ In the Global South, bamboo is a promising solution to avoid massive deforestation of tropical forests.^33,37^ Additionally, recent studies have demonstrated the efficiency of substituting GHG-intensive materials with fast-growing or herbaceous biobased materials, for example, bamboo and straw, due to their carbon removal potential and reduced life-cycle emissions.^32^ The advantage of choosing these biomasses instead of woody ones is that they exhibit a shorter rotation period of regrowth (approximately 1 year), hence a higher yield,^35^ and they are usually byproducts of croplands that can be transformed into high-value applications,^38^ which avoids land use competition between buildings and food production. Nonetheless, in the literature, there is no consensus on how to model biogenic carbon released and reabsorbed during the biobased material life cycle.^39^ The established approaches can be summarized as static 0/0 or +1/–1^40,41^ and dynamic,^42,43^ with carbon uptake before or after construction, which is able to include the impact of timing of the carbon emissions and the influence of the rotation period related to the biomass growth.^44^ Moreover, Guest and coauthors^45^ proposed an index, the biogenic global warming potential index (GWP_bio index_), which is able to directly compute the carbon dioxide regeneration with the biogenic CO_2_ pulse emissions. Indeed, this index is capable to consider the storage period of harvested biomass with different rotation periods in the anthroposphere as a negative value to be considered at the beginning of a standard life cycle assessment (LCA), both for a 100 or 500-year time horizon, in a semistatic way. Among them, the dynamic one is able to include the impact of the timing of the carbon emissions and the influence of the rotation period related to the biomass growth.^44^ The benefit of using a dynamic approach can also be appreciated in solving the inconsistency of different time frames observed with traditional LCA when replacing building assemblies and components during building service life.^12^ Only when considering these dynamics can the potential of achieving climate neutrality be estimated, and stakeholders can be assisted in defining optimized material selection strategies.

Unfortunately, not all construction materials can be replaced with herbaceous materials, and a compromise between GHG emissions and biobased materials should be made. In fact, by leveraging their negative biogenic GWP, this research proposes a new way of approaching the design of climate-neutral construction by quantifying herbaceous biomass, or climate-negative materials, needed to bring to net-zero the total embodied emissions of emitting, or climate-positive, materials. While looking for analogies with other human activities,^46^ we would like to position the debate in this current paper on the appropriate material diet required to build climate-neutral construction. Under this innovative vision, the thickness of insulation was designed for climate neutrality rather than for energy efficiency, as it is clear that, under the current energy transition goal, the net-zero emissions embedded in materials have to be the primary objective to pursue.^47^

## 2 Materials and Methods

### 2.1 Climate Neutrality at the Construction Scale Using Climate-Negative Materials

**Figure 1:**
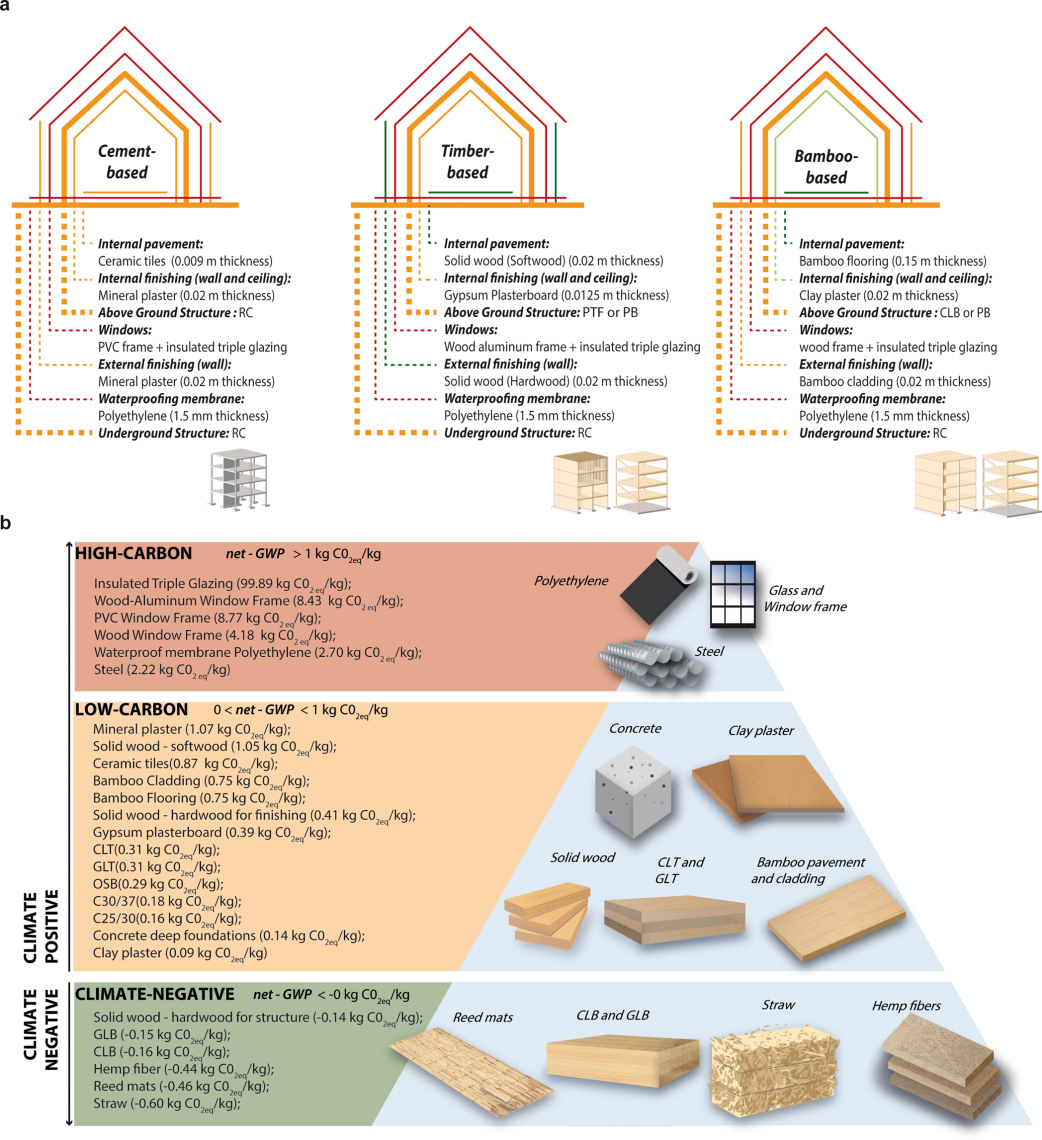
(a) Material diets. From left: Concrete-based, timber-based, and bamboo-based material diets. (b) Material classification according to the net-GWP value that divides them into climate-positive (high-carbon and low-carbon) or climate-negative materials (see Paragraph S2.3).

In this paper, the dynamic LCA approach was used to consider the time dependence of biogenic carbon and of building element replacement. We assessed three different material diets by decomposing the building into six construction elements that play a major role in building embodied emissions,^48^ namely, aboveground and underground structures, windows, waterproofing membranes, finishing—divided into internal pavement, walls, ceilings, and exterior walls—and insulation. By mixing more conventional, for example, concrete, and unconventional, for example, bamboo, ingredients as building materials, we designed different material diets to achieve climate neutrality. The material diets were defined according to the gradual use of herbaceous materials, from the insulation up to the structural level: cement-based, timber-based, and bamboo-based (Figure 1a). Each material is classified as climate-positive or climate-negative (Figure 1b) according to its carbon release and removal potential. More precisely, the materials used were divided into three main categories (i) high-carbon, (ii) low-carbon, and (iii) climate-negative materials, according to their resulting net-GWP value. The net-GWP is the sum of the GWP at a 100-year index of each material and the CO_2eq_ removal of biobased materials calculated according to the accounting approach proposed in Paragraph 2.3. The concrete-based diets with concrete structures represent the reference to the “business as usual”, as concrete will remain the reference material for a majority of construction.^49^ For all diets, the insulation materials were herbaceous ones, in particular, reed mats, straw, and hemp fibers with different carbon removal capacities. The replacement of building elements was considered. In particular, the building service life was assumed to be 60 years.^50^ The service life of the structural elements corresponds to that of the building, that is, 60 years, together with the waterproof membrane in polyethylene.^14^ All finishing, window and window frames have a service life of 30 years.^51^ Regarding the biobased insulation, 60 years were chosen as suggested by Göswein et al. 2021^52^ (see Paragraph 1.4 in the Supporting Information).

The essential information of the materials used in the project are collected in Table S4 in the Supporting Information. The λ-values were noted only for finishing and insulation materials because they were useful in evaluating the thermal performance of the external envelope, while the rest of the data were used to evaluate the net-GWP for each material.

To test climate neutrality, we focused on new residential building typologies in the European context because the European Union aims to become the first climate-neutral continent by 2050 with the “Green Deal for Europe” in line with the Paris Agreement.^4^ However, the building decomposition used, with insulation designed to compensate emissions and not to fulfil the energy requirements of building codes, makes these building typologies much more appropriate to a wider context than Europe. In particular, we utilized the four typical building typologies (BT), namely, single-family house (SFH), terraced house (TH), multifamily house (MFH), and apartment block (AB) to create the geometrical reference buildings from the Tabula/Episcope database.^53^ We reported the results only for the statistically significant values of these data sets, which are the low whisker (0th quartile), up whisker (4th quartile), and median (2nd quartile) (see Figure SI1, Table SI1, and Paragraph 1.1 in Extended Methods in the Supporting Information), to obtain three geometrical configurations that would represent the whole data set. For these geometrical configurations, a parametric model was set up to quantify the structural mass incidence per gross floor area of a given structure over the total number of stories of the building. Consequently, we computed the material quantity (kg/m_RES_^2^) and the related GHG emissions (kg CO_2eq_/kg) to calibrate the climate neutrality for the three diets. The herbaceous material quantity corresponds to a specific insulation thickness whose architectural and structural feasibility is assessed to respond to the maximal linear loads allowed according to the structural preliminary dimensioning (see Supporting Information Paragraph 1.2.4) and passive house requirements for the operational energy targets.^54^ Finally, the 100-year time horizon global warming potentials (GWP_100_) and the 20-year time horizon global warming potentials (GWP_20_) for each BT and material diet are compared for evaluation, as a long-term horizon may hide the contribution of material shifts to biobased diets to reach climate neutrality by 2050 (see Supporting Information Paragraph 3). All data were normalized according to the reference energy surface (RES).

### 2.2 Structural Mass Incidence

To define the carbon footprint of the different structural systems, a parametric model was set up in MATLAB^55^ (see Supporting Information Annex A for the script and Supporting Information Paragraph 1.2 for extended methods) to quantify the material incidence per gross floor area of a given structure over the total number of stories of the building. Reinforced concrete as well as timber and engineered laminated bamboo were chosen for the abovementioned ground structures, while the foundation was made with reinforced concrete and eventually deep foundation out of steel when needed. Moreover, four different structural configurations were defined (see Figure S2 in the Supporting Information). A reinforced concrete (RC) structural scheme was designed for concrete-based diets as in situ cast concrete columns and walls supporting a reinforced concrete plate. The structural scheme for timber-based diets represents a platform timber frame (PTF) system composed of walls with offsite assembled load-bearing elements (massive sawn timber and OSB panels) and beams in solid wood. Engineered cross-laminated bamboo (CLB) was used for the bamboo-based structural scheme, which was modeled as load-bearing walls and floor panels. Finally, a post and beam (PB) frame structure with diagonal bracing and floor panels was specifically designed for high-rise structures for both timber- and bamboo-based buildings with more than 10 stories. All values were finally normalized according to the gross floor area of the module to obtain normalized values and were applied to the different building typologies according to the diets and building height (see Figures S4–S8 in the Supporting Information).

### 2.3 Net-GWP Calculation and Climate-Neutral Construction Assessment

To quantify total CO_2eq_ emissions, we performed a dynamic LCA for all construction materials. In particular, when the materials are produced at year 1, the dynamic method can be simplified with tabulated values of the GWP as defined by the IPCC 2013 method (GWP_100,IPCC_), and Guest and co-authors simplified semistatic indices (GWP_bio index_) to account for the biogenic carbon cycle.^45^ When building elements are replaced after 30 years, both the fossil emissions (GWP_100,dyn_) and the CO_2_ uptake (GWP_bio index,dyn 31–60_) are calculated with the “DynCO2” calculation tool.^56^ The net-GWP of construction materials is the sum of the GWP at the 100-year index of each material expressed in kg CO_2eq_/kg and the CO_2_ removal of biobased materials, here called GWP_bio_.^45^ Details of the GWP_bio_ calculation can be found in Paragraph 1.5 in the Supporting Information. In this study, ISO Standard 14067:2018^57^ was used for fossil-related emission calculations, whereas EN 15804:2021^58^ was assumed to define scope and objectives, functional unit, and system boundaries. Here, the calculation was limited to the cradle to gate stages (Modules A1-3) as well as waste disposal (Module C4). For biobased materials, the waste disposal scenario considered was incineration; for steel, it was recycling, whereas for residual materials, it was landfilling.

After the computation of the total mass of the construction product used in the building, we multiplied it for each net-GWP value for the four BT and the three material diets as calculated in the climate neutrality eq 1
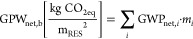
1where

- GWP_net,b_ is the specific net-GWP value calculated for each diet,
- GWP_net,*i*_ is the net-GWP value of each material, expressed in kg CO_2eq_/kg,
- *m*_*i*_ is the mass of each construction material, expressed in kg/m_RES_^2^.

The total construction positive GWP_100_, based on fossil emissions, needs to be neutralized by the fast-growing biobased insulation (see Figure S11 and Table S6 in the Supporting Information). The mass of insulation to be installed in the envelope that can compensate through negative CO_2_ emissions and the positive GWP_100_ of material production and final disposal can be calculated according to eq 2
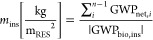
2where

- *m*_ins_ is the mass of insulation needed to achieve climate neutrality in 100 years normalized according to the RES (kg/m_RES_^2^),
- GWP_net,*i*_ is the net-GWP value of a generic noninsulating material, expressed in kg CO_2eq_/m_RES_^2^,
- GWP_bio,ins_ is the GWP_bio_ value of the selected insulation material, expressed in kg CO_2eq_/kg.

We performed this calibration with three herbaceous biobased insulation materials. From the Ecoinvent^59^ databases, we chose reed mats, which exhibited the highest net-GWP value (max), hemp fibers, which exhibited the lowest value (min), and straw characterized by a value between the two (med).

### 2.4 Architectural and Structural Feasibility Assessment

In conclusion, we evaluated the architectural and thermal feasibility of the quantity of insulation obtained. First, the wall thickness was calculated according to eq 3
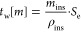
3where

- *t*_w_ is the mean thickness of the envelope,
- *m*_ins_ is the mass of insulation, in kg/m_RES_^2^,
- ρ_ins_ is the volumetric mass of the insulation, in kg/m^3^,
- *S*_e_ is the total surface of the envelope, in m^2^/m_RES_^2^_._

The total surface of the envelope (*S*_e_) is the sum of the exterior wall, roof, and basement area because we made the assumption of filling each envelope element with a constant insulation level. Second, we checked if the *U*-value of the three different material diet wall assemblies fulfilled the most stringent European passive house (*U*/value ≤0.10 W/m^2^ K^60^) standards.

Once the different biobased wall insulation thicknesses and their related thermal performance are computed, it is possible to calculate the corresponding line load on the structure to control that it does not exceed the one considered during the structural dimensioning for different material diets (see Paragraph 1.7 in the Supporting Information).

## 3 Results and Discussion

### 3.1 Material Quantities

**Figure 2:**
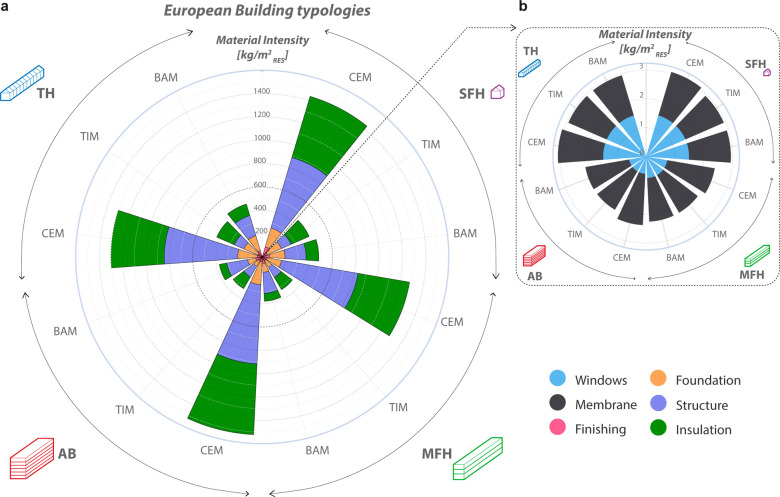
(a) Material diets shown as a pyramid logic in a wind rose graph representing the quantity (kg) of materials needed to have climate-neutral construction per m_RES_^2^ (Material Intensity). The quantity of the material is expressed for the four building typologies. Each building typology is represented here by the median geometrical configurations and median herbaceous insulation, that is straw, for the three diets, namely, CEM = cement-based diet; TIM = timber-based diet; and BAM = bamboo-based diet. (b) Zoomed in view of the windows and the waterproofing membrane to appreciate their values because they are of another order of magnitude in comparison to the other building elements. See Table S8 in the Supporting Information for the rest of the data and for the other geometrical configurations and biobased insulations. The graph was implemented in JavaScript starting with the Highcharts.com script available online.

Figure 2 shows the material quantities required for climate-neutral construction depending on the diet and the BT.

Cement-based diets are the most mass-intensive diets for all building typologies. The insulation required to bring to zero the total construction emissions ranges between 449 and 608 kg/m_RES_^2^ depending on the BT when straw is used. In contrast, bamboo-based diets are the least mass-intensive and require between 65 and 110 kg/m_RES_^2^ of straw to reach climate neutrality, even if bamboo is transported from Asian countries.^61^ Future local cultivation of bamboo in some southern European regions would further decrease the impact of bamboo-based construction. The timber-based diets are closer to the bamboo-based diets (115–170 kg/m_RES_^2^). Structure and foundation control building weight, regardless of diet. In contrast, windows and membranes have a small influence on the final mass.

### 3.2 Material Diet Index for Construction: a Ratio between Climate-Negative and Climate-Positive Materials

**Figure 3:**
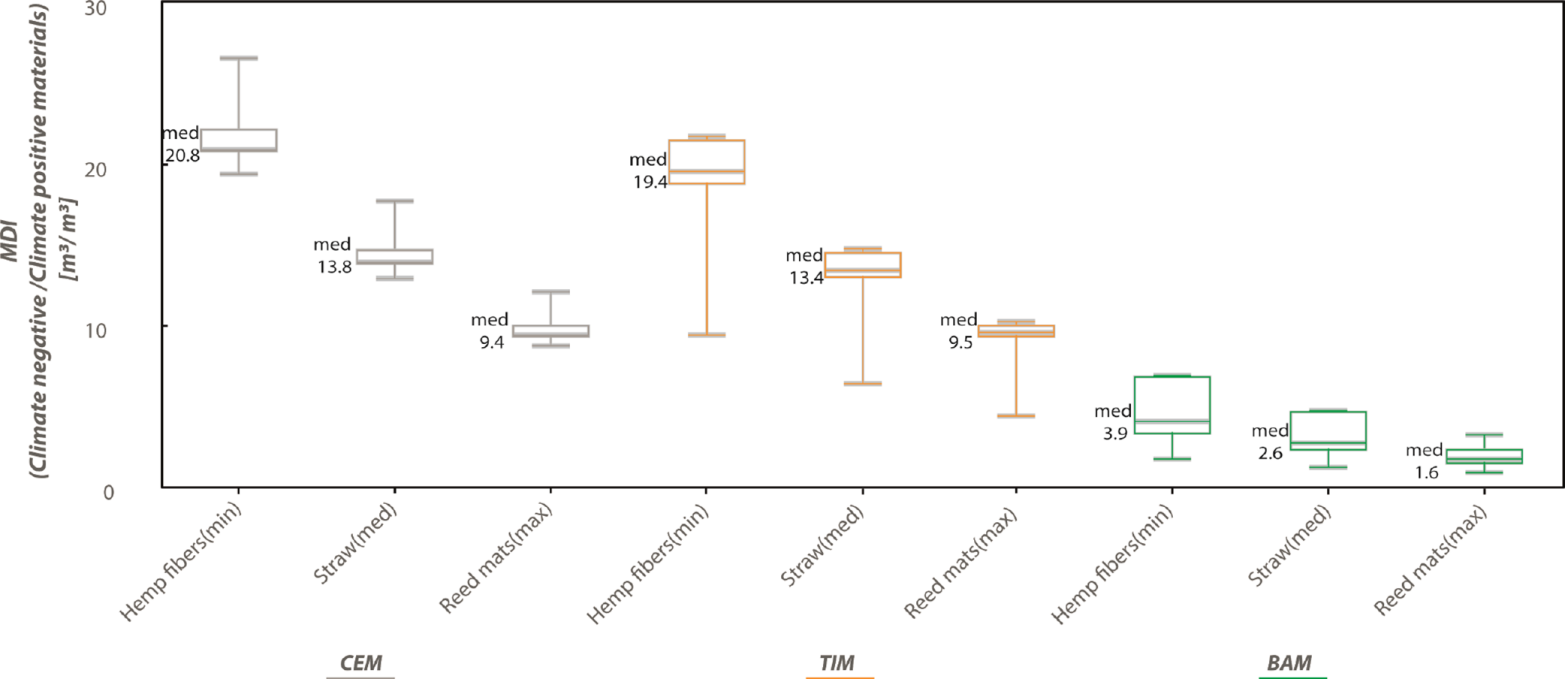
Box plot to show the MDIs between the volume of climate-negative and climate-positive materials (*y* axes) needed to reach climate-neutral construction with the use of three different biobased materials for the three diets (*x* axes), namely, hemp fibers (min), straw (med), and reed mats (max). The three diets are CEM = cement-based diet; TIM = timber-based diet; and BAM = bamboo-based diet. The graph is implemented in JavaScript starting with the HighCharts.com script available online. For the data, see Table S7 in the Supporting Information.

To select climate-negative materials for building envelopes, we performed an architectural feasibility analysis. First, the mass quantities were converted into volumetric quantities to define the spatial footprint that designing climate-neutral construction would demand. Second, to compensate for the use of climate-positive materials with climate-negative materials, the necessary volumetric ratios among these two material families, or material diet indices (MDIs), were calculated (Figure 3). The greater the value is close to 1, the greater the two material volumes (negative vs positive) are similar. The MDIs are usually greater than 1, except for one bamboo-based diet when using reed mats in an apartment block typology and up whisker building geometry. Usually, climate-negative material volumes are larger than climate-positive material volumes, whereas for this specific bamboo-based exception, we need fewer climate-negative materials to reach climate neutrality. Depending on BT and material choices, MDIs range between 0.74 and 26.46 m^3^/m^3^, indicating that every cubic meter of a carbon-emitting material, for example, glass, concrete, and so on, should be compensated by 0.74–26.46 m^3^ of climate negative-materials, that is, biobased ones. Additionally, the results highlighted that for each diet, the insulation material choice controls the MDI regardless of building typology.

### 3.3 Climate Neutrality for Construction and Energy Efficiency for Operation

The results for the envelope thermal performance (Table 1) show two possible situations. The first one is when the construction obeys the strictest *U*-value defined for the passive house standard and fulfils the operational energy requirements with the established envelope thickness. The second one is when construction does not cope with the energy requirement and therefore requires a higher insulation level. This would contribute to an additional increment of the carbon removal potential, and this extra contribution can be spent on other building components or installations, for example, PV systems and energy storage. The latter appears in very few cases, mainly for timber and bamboo-based diets, as the demonstration that the envelope composition obtained with a climate-neutral construction design strategy in most cases is able to meet the energy requirements. Consequently, designing for climate neutrality with material GHG compensation also allows energy-efficient building standards to be reached.

**Table 1:**
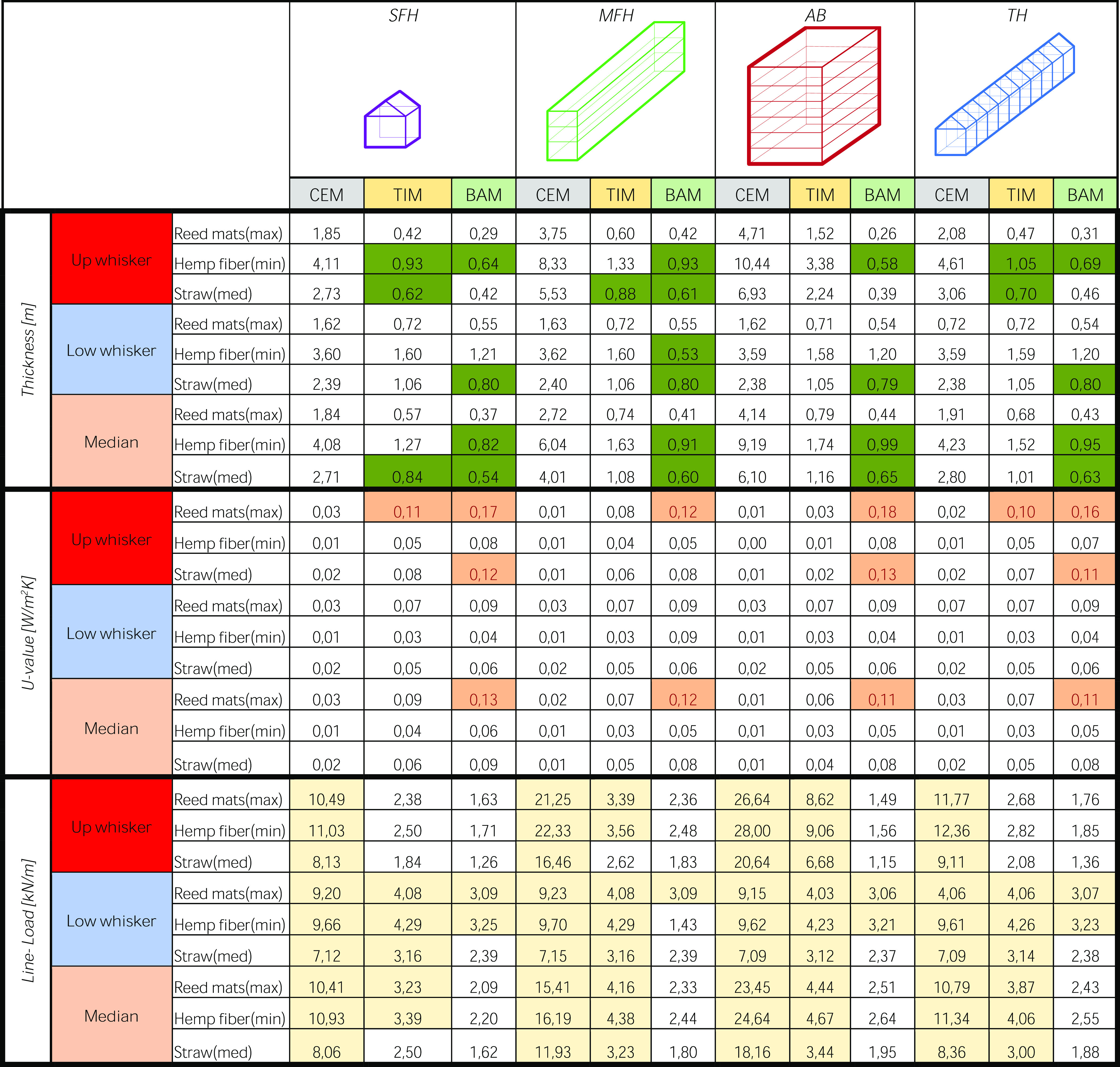
Insulation Wall Thickness for the Three Material Diets (Thickness), Their Related *U*-Values and Resulting Line-Loads for all Geometrical Configurations, the Four Building Typologies, and the Three Material Diets^a^

a Red values represent *U*-values > 0.10 W/(m^2^/K), for example, that do not respect the most stringent value for passive house standards, with the necessity of adding the insulation material to achieve the energy performance goals. Yellow-filled values represent line-load > 0.29 kN/m, for example, the maximal value calculated for the insulation material during structural predimensioning. Bold values in green cells represent thicknesses respecting both the passive house energy and structural requirements. The three geometrical configurations are Up whisker, Low whisker, and Median. The four building typologies include SFH, TH, MFH, and AB. The three diets are CEM = cement-based diet; TIM = timber-based diet; and BAM = bamboo-based diet for the three herbaceous insulations, namely, hemp fibers (min), straw (med), and reed mats (max).

### 3.4 Envelope Thickness of Climate Neutral Construction

All thicknesses reported in Table 1 correspond to climate-neutral construction depending on insulation type and BT. However, only the bold values in green cells meet both the thermal and structural requirements. For the hemp fibers (with the worst net-GWP value), the wall thicknesses can reach unfeasible values as high as 10.44 m depending on structural choices and building typology. Even with timber or bamboo structures, some BT (e.g., AB) would require 1.2 to nearly 3.38 m of hemp materials. The straw values remain for most construction solutions within an acceptable range for the wall thickness when timber or bamboo structures are used, except for the timber-based AB case with a 2.24 m wall thickness. They are usually smaller than 1 m and can be in a range of 0.39–1.16 m for many BT. With a concrete structure, the straw wall thicknesses would be larger than 2 m. For timber- and bamboo-based diets, the use of reeds results in thicknesses that are smaller than the necessary ones to respond to the thermal requirements and close to 2 m for concrete-based diets. Regardless, its larger density (180.5 kg/m^3^) with respect to straw (95 kg/m^3^) could make it less favorable when controlling the line load for structural capacity (e.g., timber-based diet for the MFH). In northern Europe, current construction usually accounts for a wall thickness of 40–50 cm.^53,62,63^ This paper showed that with straw or reed insulation, it would be possible to build similar wall dimensions with timber and bamboo structures. In contrast, concrete construction requires insulation sizes that are too large and heavy for the dimensioned structures or accepted by urban regulations, even if complying with thermal needs. Indeed, all calculated insulation thicknesses are representative of climate-neutral construction. Nonetheless, larger thicknesses correspond to more thermal performant walls but heavier and urban-impeding solutions, as in the case of cement-based diets; in contrast, lighter but less thermal performant solutions are correlated to smaller thicknesses, such as in the case of bamboo-based diets. The timber-based ones are similar to the bamboo-based construction but still require a more insulation material. These results highlight the need for a tradeoff between material embodied emissions and structural and thermal performances. Hence, once the basic requirements in terms of GHG emissions are set and the corresponding dimension of insulation is defined, the typical iterative design process should be performed to ensure an optimal configuration of the final building in terms of other performances (e.g., structural consistency, operational energy, sound proofing, fire resistance, etc).

### 3.5 Recommendations for Immediate Climate Neutrality in New Construction

Our findings demonstrate that it is possible to build climate-neutral construction thanks to the use of herbaceous biobased insulation materials. The building element dimensions can be controlled, and the thermal performance is for most cases satisfied in accordance with the high-energy efficiency standard. In fact, the contemporary construction built with straw has similar thicknesses (e.g., architect Werner Schmidt’s straw-bale construction with 0.80 m thick walls^64^). Hence, new climate-neutral construction would have a similar appearance as the nonconventional biobased ones currently built in northern countries, and construction technologies already available on the market can be used. The only exception is the CLB, which is used as the structural material for tall construction, that is, more than three stories, is limited thus far.^65^ We included the scenario of having multistory construction with CLB in the perspective that the market will move in this direction soon.

According to our results, we can then build climate-neutral construction that complies with the operational energy requirements and avoids the carbon lock-in situation that is feared when energy-saving requirements are implemented without considering the consequential embodied emissions.^19^

Regarding the structural design, we did not dimension the timber and bamboo elements according to fire safety requirements. Nevertheless, no exposed structural membranes are assumed in the design because protecting layers out of gypsum or clay plaster are assumed for fire protection.^66^ Another assumption we made is the possibility of adopting biobased insulation for basement insulation, which is not recommended due to the high water absorption risk and consequential fast decay. To reduce the risk, we added a waterproofing membrane that increases embodied emissions but removes high-moisture content risks. An alternative biobased solution would be cork due to its nonputrescible properties, but costs and availability make it difficult to reach the full European market.^67,68^

Finally, it is important to mention that the GHG-fossil emissions linked to the use of concrete could be further reduced by implementing low-carbon concrete solutions.^27,28^ Another strategy is the optimization of structural concrete design, which could be facilitated in the near future by BIM and automation construction.^69−71^ In this paper, we used conventional concrete emissions to represent the business as usual in our construction practices, but available alternatives allow us to reduce concrete-based emissions by a factor of 2.^23,26^ This would lower the insulation volume by the same order of magnitude and therefore lead to the possibility of building concrete climate-neutral construction with wall dimensions similar to those of current construction but still larger than those needed in the case of timber and bamboo structures. Finally, the results show that some building typologies are much more favorable for climate-neutral construction than others. MFHs and THs require a much lower wall thickness than ABs. This should provide a strong incentive for city planners to densify cities where MFH and TH are more favorable than AB.

Our work provides a practical approach that can be used by policy makers to propose incentives for climate-negative technologies for the building and construction industry. Moreover, thanks to this concept, designers can be assisted during the early-design phase and become aware of the embodied GHG emissions resulting from their construction material choices and the physical ratios (MDIs) among the climate-negative and climate-positive ones. These preliminary considerations could guide them in the choice of the structural solutions and the resulting envelope dimensions that could be limited by urban-planning regulations.

### 3.6 Calculating Immediate Climate Neutrality with the LCA Method

**Figure 4:**
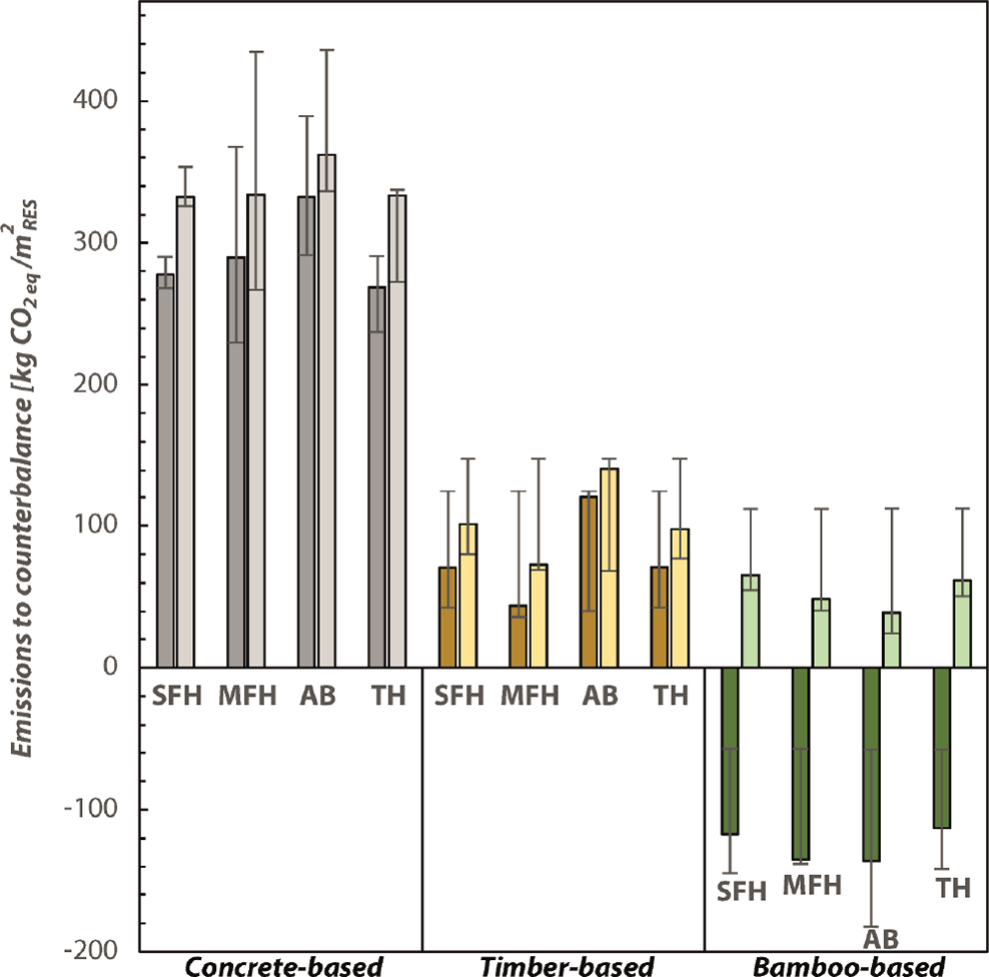
Emissions expressed in kg CO_2eq_/m_RES_^2^ to neutralize by biobased insulations. The values are reported for all material diets, building typologies and if the GWP used is with a time horizon of 20 or 100 years. The four building typologies include SFH, TH, MFH, and AB. The three diets are the cement-based diet, timber-based diet, and bamboo-based diet.

It is important to note that all calculations presented in the current work were performed with the GWP_100_ impact assessment method within a 100-year time horizon. Although this is the calculation method used in most climate models and LCA calculations of construction, a long time horizon may hide the contribution of material shifts to biobased climate neutrality in the transition period before 2050. The closest calculation method to approximate this immediate climatic goal would be to calculate the GWP with a time horizon of 20 years (GWP_20_). Figure 4 shows the deviation of the results between GWP_100_ and GWP_20_ for each BT and material diet when all materials, except the compensating insulation, are considered (see Supporting Information Paragraph 3). More precisely, the total construction emissions to be neutralized by means of biogenic insulation are presented. It clearly shows that no significant differences can be seen for concrete and timber-based diets, whereas emissions from the bamboo-based diet become negative even without insulation. This is due to the very short rotation period of bamboo and would indicate that the construction is climate-negative after 20 years (in 2040) and finally becomes climate neutral in a longer time span (by 2120) when biogenic CO_2_ is emitted at the end of the building service life. Consequently, it confirms the main results of this paper, showing that it is possible to reach climate neutrality at the building scale and before 2050, when biobased insulations are used and combined with appropriate building typology (MFH and TH).

## Supporting Information Available

Methods and results and MATLAB Script (PDF)

## Abreviations

GHG: greenhouse gas
GWP: global warming potential
LCA: life cycle assessment
GWP_bio index_: biogenic global warming potential index
BT: building typologies
SFH: single-family house
TH: terraced house
MFH: multifamily house
AB: apartment block
SI: Supporting Information
RC: reinforced concrete structural scheme 1
PTF: platform timber frame structural scheme 2
CLB: engineered cross-laminated bamboo structural scheme 3
PB: posts and beams structural scheme 4
RES: reference energy surface
MDI: material diet index
