# Supplementary material for: Material Diets for Climate-Neutral Construction
Source: Environ Sci Technol. 2022 Apr 4;56(8):5213–23. doi: 10.1021/acs.est.1c05895 (PMC9022436; doi:10.1021/acs.est.1c05895)
Supplement: Supplementary file 1 — es1c05895_si_001.pdf [file es1c05895_si_001.pdf]

Supporting Information (SI) for:

Material diets for Climate-Neutral Construction

Olga Beatrice Carcassi[1*], Guillaume Habert[2], Laura Elisabetta Malighetti[1], Francesco Pittau[1,2]

[1] Department of Architecture, Built environment and Construction engineering (ABC), Politecnico di Milano, Via G. Ponzio 31, 20133 Milan, Italy
[2] Department of Civil, Environmental, and Geomatic Engineering, ETH Zurich, Stefano-Franscini-Platz 5, CH-8093 Zurich, Switzerland

[*] Corresponding author: Department of Architecture, Built environment and Construction engineering (ABC), Politecnico di Milano, Via G. Ponzio 31, 20133 Milan, Italy.
E-mail: olgabeatrice.carcassi@polimi.it , phone: +39 331 210 6578

This document, which is the Supporting Information accompanying the main article, provides additional information on the methods, results and it includes the MATLAB Script in Annex A.

Contents:

1. ***Extended Methods*** **2**
2. ***Extended Results*** **18**
3. ***Further analysis: Contribution to global warming with a time horizon of 20 years (GWP_{20})*** . **24**
4. ***References*** **25**
5. ***Annex A*** **28**

1. Extended Methods

1.1. Geometric parameters for the Building Typologies

The TABULA/Episcope database [1] contains the main information about the composition of the reference residential buildings which are representative of the stocks across 21 European Countries, where more than 2700 buildings are analyzed and collected.
From the excel file "tabula calculator.xlsx, the data that have been considered are:
- A_C_Storey = single conditioned storey surface [m^2],
- N_Storey_effective_envelope = number of conditioned storeys [-],
- A_Esto, _W all_ExtAir = exterior wall surface [m^2],
- A_Estim_Window = window surface [m^2].

Further simplifications were made in the definition of the geometric configurations. More precisely, the single area for every floor was kept the same for each storey. All the data of the different BT collected from TABULA/Episcope database were normalized according to the Reference Energy Surface (RES). RES is the total surface of the conditioned building, which in this case was the single conditioned storey surface multiplied by the number of conditioned storeys.
Usually, the material used for the windows have high environmental impacts. For this reason, we calculated the emissions resulting for finishing and waterproofing membrane (see paragraph 2.1 in main paper) and the structures (see in paragraph 2.2 in main paper) for the three diets and assigned the higher window surfaces to the most polluting geometric configurations for each building typology (see final parameters in Table S1).

1.1.1. Supplementary Figures and Tables

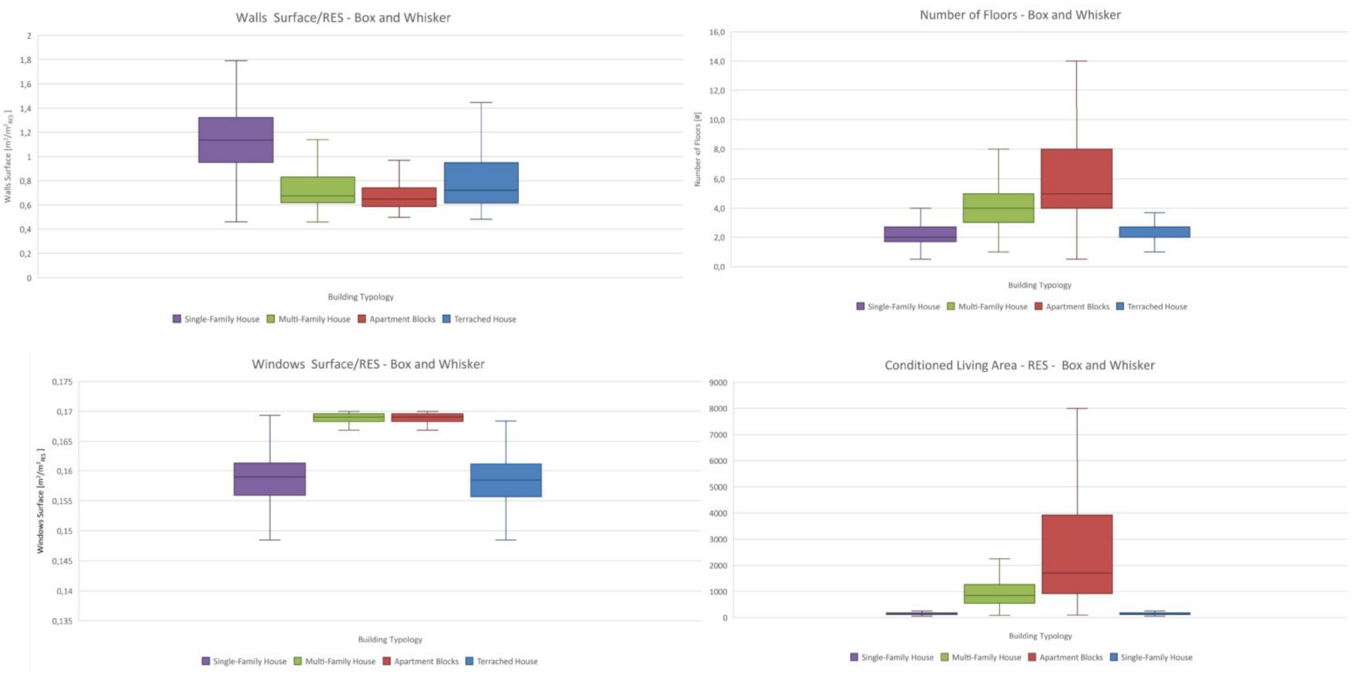

Figure S 1: Box and whisker statistical representations for the geometrical parameters extracted from the Tabula/Episcope Database. Top left: wall surface normalized according to the RES. Top right: number of conditioned storeys. Bottom left: window surface normalized according to the RES. Bottom right: conditioned living area, or RES

			SFH	MFH	AB	TH
Building Geometry from TABULA/Episcope	UP Whisker	RES [m²] (reference for normalization)	255	2250	7997	297
		Number of conditioned storeys	4	8	14	4
		Roof area = basement= area single floor [m²/ m²$_{RES}$]	0.25	0.13	0.07	0.25
		Exterior Wall area [m²/ m² $_{RES}$]	1.79	1,14	0.97	1.45
		Window area [m²/ m² $_{RES}$]	0.17	0,17	0.17	0.17
	LOW Whisker	RES [m²] (reference for normalization)	53	86	93	60
		Number of conditioned storeys	1	1	1	1
		Roof area = basement= area single floor [m²/ m²$_{RES}$]	1.00	1.00	1.00	1.00
		Exterior Wall area [m²/ m² $_{RES}$]	0.46	0.46	0.5	0.48
		Window area [m²/ m² $_{RES}$]	0.15	0.16	0.17	0.15
	MEDIAN	RES [m²] (reference for normalization)	145	842	1702	136.7
		Number of conditioned storeys	2	4	5	2
		Roof area = basement= area single floor [m²/ m²$_{RES}$]	0.50	0.25	0.20	0.50
		Exterior Wall area [m²/ m² $_{RES}$]	1.13	0.68	0.65	0.72
		Window area [m²/ m² $_{RES}$]	0.16	0.17	0.17	0.16

Table S 1 – Summary of the statistical geometrical values extracted from TABULA/Episcope database and elaborated

1.2. Structural mass incidence

1.2.1. Definition of the gravity frame systems

To define the carbon footprint of the different structural systems, a parametric model was set up to quantify the material incidence per gross floor area of a given structure over the total number of stories of the building. Four different structural configurations were defined, two foresee buildings with up to 20 stories, while the other two are designed for a maximum building height of 10 storeys. The first step of the structural design is the relevance of the technological options for each diet. Reinforced concrete as well as timber and engineered laminated bamboo were chosen for the above ground structures, while the foundation was done with reinforced concrete, and eventually deep foundation out of steel when needed. Reference numbers of conditioned storeys were the one collected and elaborated in the geometric parameter phase (see Table S1) and applied to the four structural schemes.

The structural systems were designed considering the building height as main driving parameter. It was assumed that for buildings up to 14 storeys (as in AB case) lateral stiffness is not a conditioning factor, as the design is mostly controlled by vertical strength [2]. Nonetheless, a requirement for global stability, as well as provision for adequate strength against horizontal actions, may remain relevant even for low/mid-rise buildings. Consequently, simple pinned gravity frames were assumed for designing the four alternative structural systems, as shown in Figure S 2.

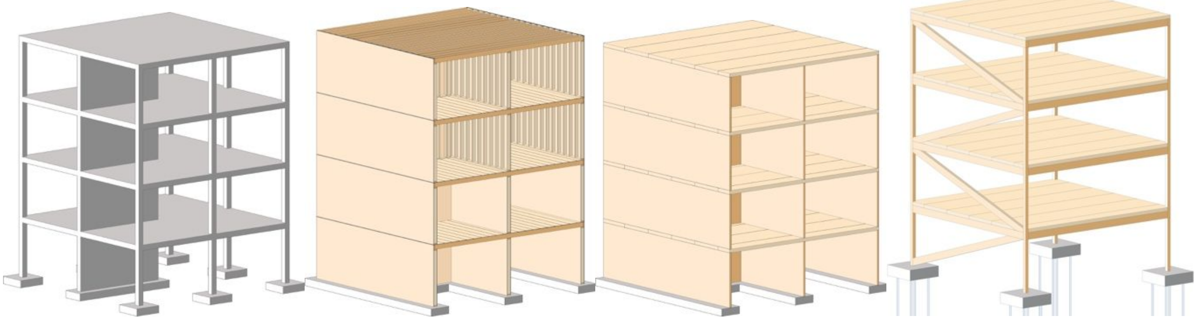

Figure S 2 - Structural schemes and related material diets. From the left: reinforce concrete (RC) cement-based diet; platform timber frame (PTF) timber-based diet; cross laminated bamboo (CLB) bamboo-based diet; post and beams (PB) timber-based and bamboo-based diets

The first scheme consists of a reinforced concrete gravity frame with shear walls (RC), and it was designed for the cement-based diet. The second scheme, designed for timber-based diet, consists of a platform timber frame (PTF) where wooden post and beams are used for walls, floors and roof with a distance of 60 cm, and completed by OSBs on both sides. The third scheme, designed for bamboo-based diet, is based on a platform frame concept as well where both walls and slabs are made out of cross-laminated bamboo panels (CLB). Last scheme, specifically designed for both timber and bamboo-based buildings with a number of storeys n_{floor}>10, consists of a glue-laminated timber frame stiffened by glulam bracing which supports either CLT or CLB panels. For option 1 (RC) the basement system is based on simple squared spread footing underneath each pillar while a wall footing supports concrete shear walls and CLB/timber frames. Additional friction piles are supposed to be anchored on concrete pad foundations for option 4 (PB).
For each scheme, the vertical and horizontal loads are resisted by two separate sub-systems: pillars/panels and beams are designed to react to vertical loads while the shearing walls or the bracing system to react to horizontal loads and are assumed to give the lateral stiffness to the whole structure.
We have focused the structural analysis on the gravity frame sub-system of post, beams and columns (RC, PTF and PB) as well as cross-laminated panel (timber or bamboo), calculated as vertical load resisting system. Contrarily, no structural model was implemented for lateral load resisting sub-systems and their mass contribution was simply assumed to have a compatible dimension with the vertical load resisting sub-system (e.g., same thickness as the rest of the structure at the same floor).

1.2.2. Parametric model

The parametric model was coded in MATLAB [3] (see Annex A for the script) and defined the minimal load-bearing areas of columns, beams, walls and slabs, to support the structural, windows, finishing and insulation loads under two combinations: service state limits and ultimate state limits. The model was based on simplified modular geometries, with a mesh 10x10m and a floor height fixed of 3.2m and variable number of storeys in a range between 1 and 20 (Figure S3). The parametric model is based on simple geometry which is assumed to be a representative portion of a whole modular structure. A small set of input parameters were used, described as follows according to Figure S 3:

- *geometric parameters*: L_x and L_y indicate respectively the primary and secondary bay spans, while H_{floor} is the inter-storey height.
- *topological parameters*: n_{floor} is the total number of floors.
- *loading parameters* (characteristic values): $q_{k,floor}$ and $g_{k,floor}$ are respectively the imposed variable floor load and the permanent floor load due to floor finishes, ceiling, services and partitions, whereas g_{env} is the line-load (i.e., in kN/m) due to the building's cladding and envelope walls.

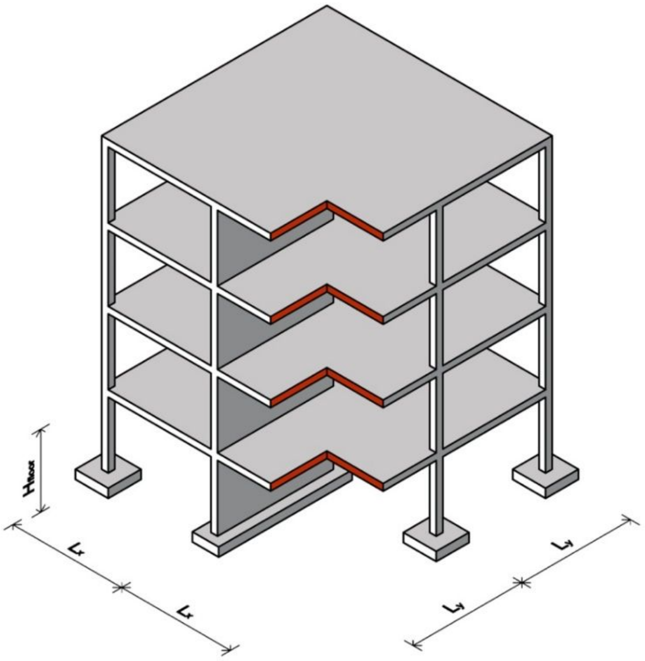

Figure S 3- Modular structural model. Lx = Ly = 5m; H floor= 3,2 m

In order to automate the task of calculating the structural masses for the entire population of reference buildings, a set of computer algorithms were specifically developed in MATLAB programming language. Given the geometric and topological input parameter values, for each structural option the sizing of each structural member was calculated assuming n_{floor} as variable between 1 and 14 (max number of storeys assumed). The structural analyses are iteratively performed as part of an optimization phase, employed to minimize the cross-section of structural members against a set of constraints. All constraints are introduced to account for a series of SIA-based design requirements at both Serviceability Limit State and Ultimate Limit State to verify the resistance and stability as well as limiting the deflection. For each combination of input parameters, the stress tension matrix was calculated, and a reverse verification model applied in order to optimize the minimal cross-sections for all members and a minimum reinforcement content for concrete elements. Specifically, the optimization method enables to find the minimum cross-sectional area of beams such that design requirements for bending resistance, shear and deflection are all satisfied, as well as to find the minimum cross-sectional area of columns against the requirements for compressive resistance and axial buckling.

For concrete vertical members a minimum thickness of 200 mm was assumed both for columns and walls. For RC, the sequential search is performed on a range of square sections at size increments of 10 mm for columns, while beams are assumed to be rectangular with a fixed height of 250 mm and a variable width. For PTF, all vertical wooden posts are assumed to have a width equal to 60 mm, a structural pitch of 600 mm and a net span of 3.2 m. Similar assumptions were made for beams, with a simply supported configuration and a span of 5 m. For CLB, a minimum thickness of 120 mm for engineered bamboo panels was assumed were used for walls, while 160 mm is the minimal thickness assumed for floors and roof.

Overall masses of steel reinforcement are estimated ex post, as a percentage of the concrete mass, specifically: 12.5‰ for columns, 10.5‰ for beams and 8.5‰ for floor slabs. The percentage values are based on practitioners' estimates. To take into account the influence of design rationalization on structural masses, the optimized cross-sections are rounded-off into groups: a uniform cross-sectional area is assumed for all columns that are vertically aligned, taken as the biggest area section within that line of columns. Similarly, two cross-section designations are considered within each floor, one for primary beams and one for secondary beams.

Foundation sizing was calculated according to SIA262, assuming a sand ordinary ground with a compressive strength $\sigma_{b,d}$=700 kN/m^2 and a maximum acceptable compression of the ground c_c=30%. A continuous 100 mm concrete was assumed to level the ground and a minimum 25 cm

of concrete used for footing, with an incremental thickness of 10 mm and a minimum reinforcement content equal to 3.0‰.
In case of PB, a group of four steel friction piles are used under each pad foundation to reduce the stress on concrete, with a minimum size of 2x2m. The length of the pales was assumed to be equal to half of building height, the nominal diameter d_p= 100 mm and the thickness of the section t_p= 10 mm

1.2.3. Output of the structural parametric models

The output of the static parametrization was the amount of materials used for each material diet structure expressed in m^3 normalized according to the gross area by varying the number of storeys (Figure S 4-8). Since for the rest of the materials we normalized for the RES resulting from the geometric configurations, we assumed that the structural normalization is equal to the normalization to the RES. Therefore, to have integrity and correspondence with the unit of measurement, we also expressed the structural incidence per m^2_{RES}.
For each geometric configuration, we extracted the value corresponding the number of storeys for each material diet to be used as the structural volumetric incidence. To obtain the mass incidence, we divided these values for the related material density.

a b

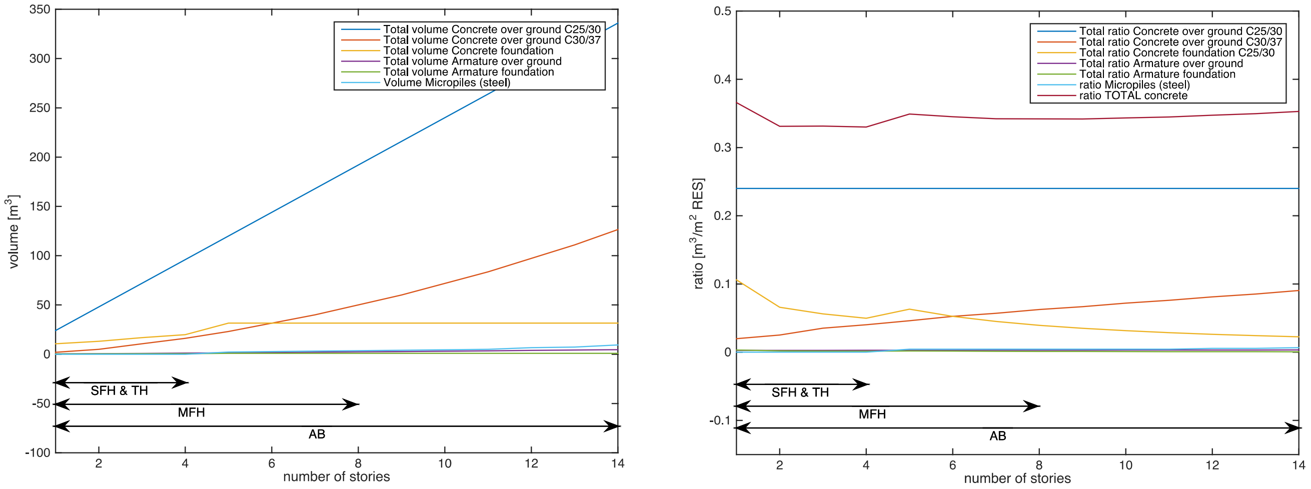

Figure S 4 – Cement-based diet, structural scheme RC – reinforced concrete. a) states for total volume; b) states for ratio volume/ m^2_{RES}

a b

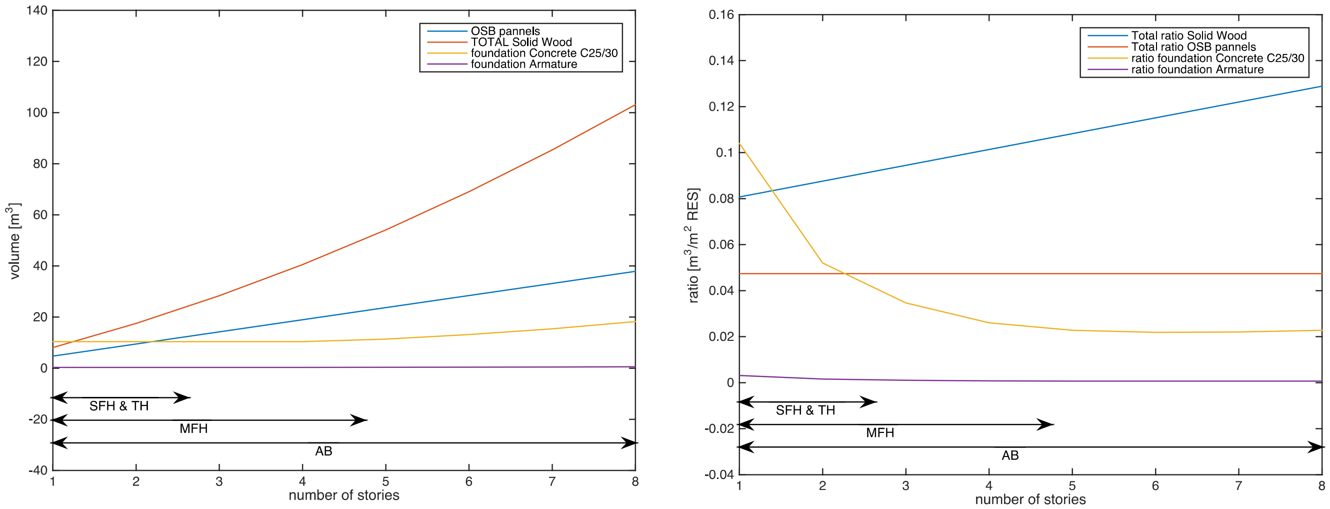

Figure S 5 – Timber-based diet, structural scheme PTF - platform timber frame (max 10 storeys). a) states for total volume; b) states for ratio volume/ m^2_{RES}

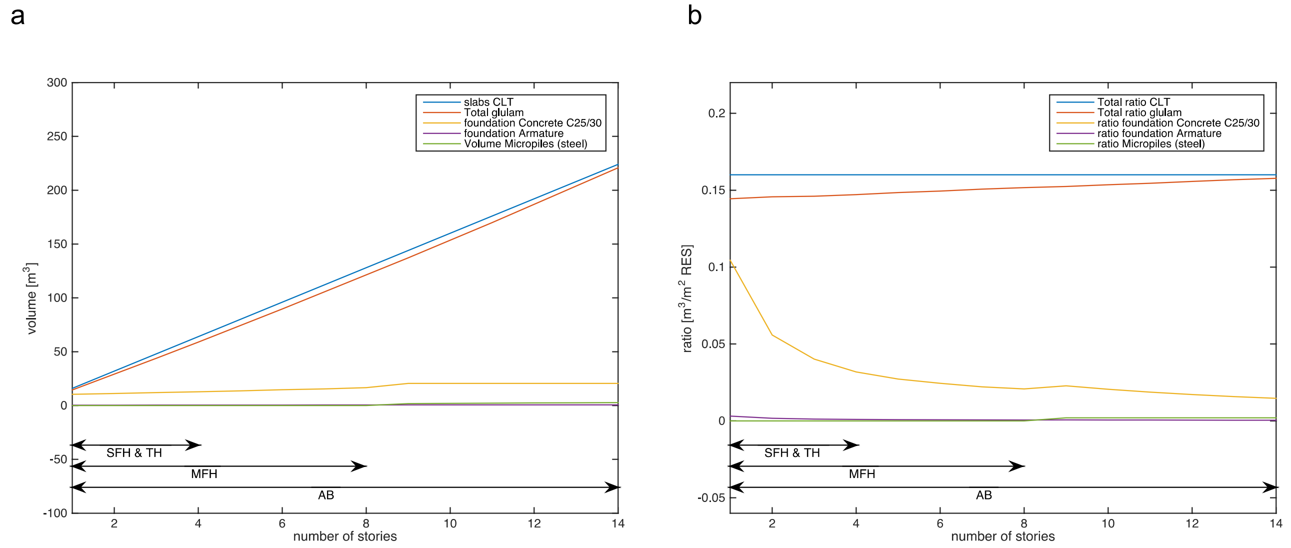

Figure S 6 – Timber-based diet, structural scheme PB – posts and beams (more that 10 storeys). a) states for total volume; b) states for ratio volume/ m^2_{RES}

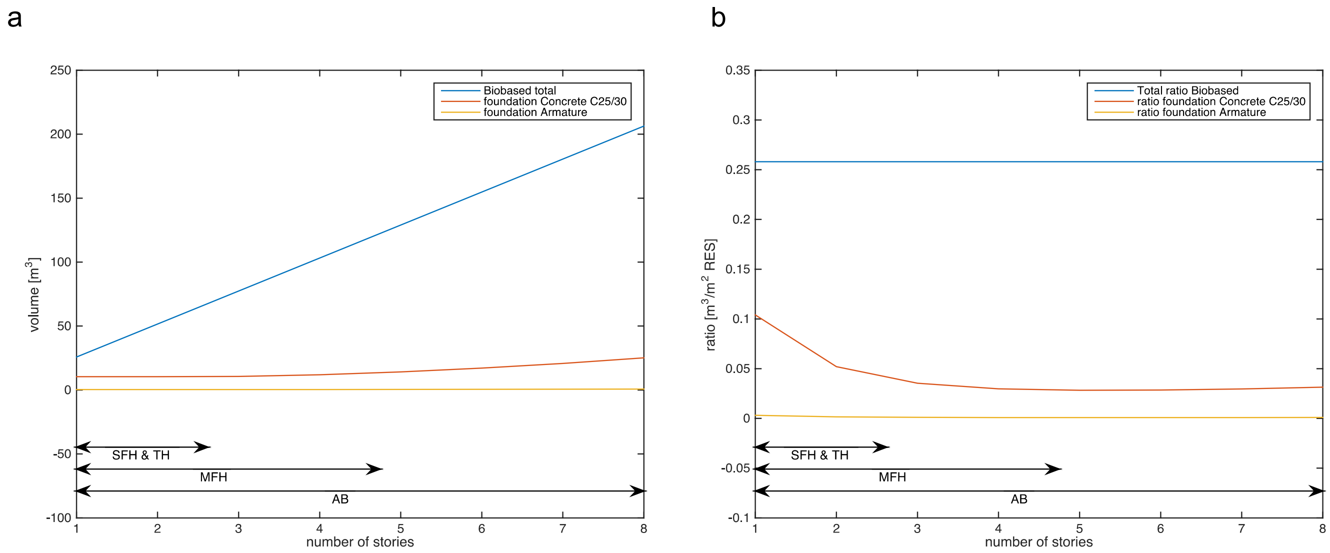

Figure S 7 – Bamboo-based diet, structural scheme CLB – cross laminated bamboo (max 10 storeys)). a) states for total volume; b) states for ratio volume/ m^2_{RES}

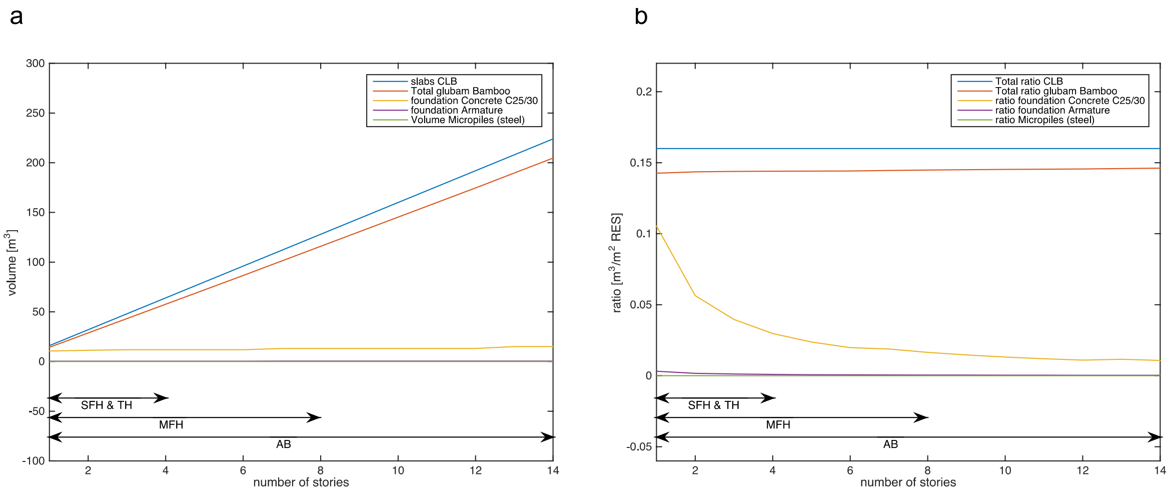

Figure S 8 – Bamboo-based diet, structural scheme BP – posts and beams (more than 10 storeys). a) states for total volume; b) states for ratio volume/ m^2_{RES}

Figure S 4.a shows the evolution of the material volumes for the cement-based diet. These are subdivided into concrete and steel and further depending on their applications. Overground structure is differentiated from the foundations and the different concrete used are kept apart, as these present a different carbon footprint. The slabs made of C25/30 increase linearly over the number of stories, as their sizing is only depending on geometric inputs and results independent by the number of floors. On the other hand, the vertical members made of C30/37 increase linearly as they result proportionally depending on the number of storeys. Foundations are made of C25/30 and their volume increases to overcome the increasing loads of the building to keep the concrete and soil compression under the material characteristic strength. At five stories, the building is too heavy, and the soil compression would provoke a failure of the compressed soil under the foundation. To prevent from this situation, micro piles were added under the pad foundations. A larger volume of footing (2x2 m) is needed to accommodate the group of micro piles under the columns and 2 m width under the walls. This explains the increased values of foundation volumes, which results constant for the following configurations, increasing only the number of micro piles. The share of steel compared to the total volume is almost insignificant, what has not to be misinterpreted with an insignificant contribution to climate change. In fact, carbon footprint of steel is almost ten times more than concrete per volume. Figure S 4.b displays the volumes of the materials normalized over the RES. The constant behavior of the slabs can be seen, where vertical elements increase almost linearly. These become rapidly dominant over the entire building materials, while the concrete foundation is absorbed with the increasing story height. The cumulative line, which is the sum of all concrete contributions, displays a constant evolution of the total concrete used in the entire building. From this last line, it can be concluded that low/mid-rise RC structures need roughly the same amount of volume per RES, no matter how tall the building is, which range a value around 0.33÷0.36 m^3/m^2.
Contrarily, the timber-based diet for building with $n_{floor} \leq 10$, Figures S 5, shows a non-linear evolution of total volume of solid wood, which corresponds to a linear increase of wood volume per RES, while for concrete in foundation an evolution similar to cement-based diet was achieved. In case of PTF, low-rise buildings account for a lower wood incidence per RES than mid-rise buildings. When the structural configuration changes to PB, see Figure S 6, a similar evolution was registered for glulam which composes the vertical and horizontal linear membranes (columns and beams), with a lower sensitivity to building height compared to PTF case, ranging a mean value around 0.15 m^3/m^2. For bamboo-based diet, see Figure S 7, in case of CLB most of the structural mass of the laminated bamboo panels is allocated to the floors, which resulted in a nearly constant incidence per RES. Finally, in case of PB, a resulting similar volume incidence to the case of timber-based diet was achieved, with a mean value of bamboo glulam of around 0.148 m^3/m^2 and a similar volume incidence of cross-laminated panels for floors (0.16 m^3/m^2).

1.2.4. Insulation line-load

Since the thickness of the insulation material, and therefore its line-load, is and output of this research, during the structural preliminary dimensioning in the envelope walls parameter, we defined as permanent load of the insulation a value corresponding to the straw, which is the median herbaceous biomass here chosen. According to the literature, the range of the wall thickness obtained when building with biobased insulation material, such as straw, varies between 40-80 cm [4–6]. To give some margin, we extended the wall thickness to 1 m. Considering a density of straw equal to 95 kg/m^3 [7], the insulation permanent line-load ($g_{env,ins}$) on the structure is equal to as 0,93 KN/m, as shown in Equation (1):

$$g_{env,ins} = \rho \cdot D \cdot H\,floor \cdot g \cdot 10^{-3} = 95\frac{kg}{m^3} \cdot 1m \cdot 3.2m \cdot 9.8\frac{m}{s^2} \cdot 10^{-3} = 2.98 \quad [kN/m] \qquad (1)$$

where:

- D = Material Thickness (m), and in this case 1 m for the straw buildings;
- ρ = density of the biobased insulation material (kg/m^3) (in this case straw is equal to 95 kg/m^3);
- g = gravity on Earth;
- 10^{-3} is the conversion factor from N to kN.

1.3. Construction materials and Net-GWP computation

Non-biobased, or "Mineral", are materials not composed by biogenic mass. In this investigation we assumed:

- glass for the windows;
- PVC, wood-aluminum and wood window frames;
- polyethylene water proofing membrane;
- steel for the reinforced concrete structure;
- gypsum plasterboard, mineral plaster, ceramic tiles and clay plaster as internal ceiling and wall finishing.

The biobased ones were divided in slow-growing, or "Timber-based", and fast-growing, or "Herbaceous", which is related to the time the plant needs to completely regrow before being clear-cut and harvested again.

Plants, whose time needed to regrowth is larger than 10 years, contributed to provide slow-growing biobased materials, e.g. timber, wood fibers, cellulose flakes, etc.

In this project, five types of forest products were adopted for different applications:

- solid wood used for structural and finishing applications. It is subdivided in softwood and hardwood;
- glued laminated timber (GLT), is well suited for structural applications;
- oriented strand board (OSB) is used for structural applications;
- cross laminated timber (CLT) is used for structural applications.

All these types differ in the fabrication process, nevertheless, are available worldwide. In this project, the regeneration period of coniferous forests for softwood supply, used in load-bearing elements and finishing, is assumed to be 90 years [8].

Plants with regrowth period lower than 10 years are categorized as fast-growing biobased materials, namely:

- bamboo for structural or finishing applications with a regeneration period of 5 years;

straw, hemp fibers and reed mats as insulation materials with a regeneration period of 1 year.

We extracted all the materials' properties either from KBOB[9] or from the scientific literature, whereas the GWP_{100} were processed by using SimaPro 8, accessing the Ecoinvent v.3 database [10] + method 2013, at 100 years (see Table S 2).

In particular we took the following processes from Ecoinvent[10] as shown in Table S 2.

As the dynamic LCA requires single GHG inputs, missing processes from existing LCA databases cannot be substituted with data from available EPDs. For this reason, the only main assumption we made is for modelling hemp processes, whereas for reed and straw secondary data from ecoinvent database were used.
Recent papers dealing with carbon footprint evaluation of agricultural practices for crops [11,12] demonstrated that the fertilization, in particular the level of nitrogen per hectare, contributes to the largest share of emissions. This is mostly dependent to the quality of the soil rather than type of crop. Thus, we decided to consider ecoinvent processes for maize seed production as representative for every missing germination processes, as for hemp species.

**Material description**	**Ecoinvent materials/process**	**Amount**	**Unit**
Insulated Triple Glazing	Glazing, triple, U<0.5 W/m2K {RER}| production | Alloc Def, U	1	m^2
Wood-Aluminum Window Frame	Window frame, wood-metal, U=1.6 W/m2K {RER}| production | Alloc Def, U	1	m^2
PVC Window Frame	Window frame, poly vinyl chloride, U=1.6 W/m2K {RER}| production | Alloc Def, U	1	m^2
Waterproof membrane Polyethylene	Fleece, polyethylene {RER}| production | Alloc Def, U	1	kg
Wood Window Frame	Window frame, wood, U=1.5 W/m2K {RER}| production | Alloc Def, U	1	m^2
Steel (reinforcement)	Reinforcing steel {GLO}| market for | Alloc Def, U	1	kg
Mineral plaster	Lime mortar {CH}| production | Alloc Rec, U	1	kg
Ceramic tiles	Ceramic tile {CH}| production | Alloc Def, U	1	kg
Cross Laminated Timber (CLT)	Glued laminated timber, for indoor use {RER}| production | Alloc Def, U	1	m^3
Glulam (GLT)	Glued laminated timber, for indoor use {RER}| production | Alloc Def, U	1	m^3
OSB	Oriented strand board {RER}| production | Alloc Def, U	1	m^3
Gypsum plasterboard	Gypsum plasterboard {CH}| production | Alloc Def, U	1	kg
Concrete C30/37	Concrete, 30-32MPa {CA-QC}| concrete production 30- 32MPa, RNA only | Alloc Def, U	1	m^3
Concrete C25/30	Concrete, 25MPa {CA-QC}| concrete production 25MPa, RNA only | Alloc Def, U	1	m^3
Concrete deep foundations	Concrete, 20MPa {CA-QC}| concrete production 20MPa, RNA only | Alloc Def, U	1	m^3
Solid wood (softwood)	Sawnwood, softwood, dried (u=20%), planed {RER}| production | Alloc Def, U	1	m^3
Clay plaster	Clay mortar {CH}| production | Alloc Def, U	1	kg
Laminated bamboo for flooring and cladding	Steel, low-alloyed, hot rolled {GLO}| market for | Alloc Rec, U	50.56991	kg
	Sawnwood, board, hardwood, raw, dried (u=20%) {GLO}| market for | Alloc Rec, U	0.088957	m3
	Wooden board factory, organic bonded boards {GLO}| market for | Alloc Rec, U	3.22E-08	p
	Sawnwood, board, softwood, raw, dried (u=20%) {GLO}| market for | Alloc Rec, U	1.237658	m3
	Electricity, medium voltage {CN}| market group for | Alloc Def, U	9.6692	kWh
	Transport, transoceanic freight ship/OCE U	14439.3	tkm
Solid wood (hardwood)	Sawnwood, hardwood, raw, dried (u=20%) {RER}| production | Alloc Def, U	1	m^3
Engineered bamboo (GLB/CLB)	Sawnwood, board, softwood, raw, dried (u=20%) {GLO}| market for | Alloc Def, U	1.297195	m^3
	Urea formaldehyde resin {GLO}| market for | Alloc Def, U	11.36229	kg
	Wooden board factory, organic bonded boards {GLO}| market for | Alloc Def, U	3.15E-08	p
	Heat, central or small-scale, other than natural gas {RoW}| heat production, hardwood chips from forest, at furnace 50kW | Alloc Def, U	2102.592	MJ
	Electricity, medium voltage {CN}| market group for | Alloc Def, U	122.1446	kWh
	Diesel, burned in building machine {GLO}| market for | Alloc Def, U	31.81442	MJ
	Transport, freight, sea, transoceanic ship {GLO}| processing | Alloc Def, U	14439.3	tkm
Hemp fibers	Maize seed, Swiss integrated production, at farm {CH}| production | Alloc Rec, U	19.23077	kg
	Tractor, 4-wheel, agricultural {CH}| production | Alloc Rec, U	1.515115	kg

	Agricultural machinery, unspecified {CH}| production | Alloc Rec, U	3.033346	kg
	Shed {CH}| construction | Alloc Rec, U	0.009113	m²
	Polyethylene, high density, granulate {RER}| production | Alloc Rec, U	0.384615	kg
	Extrusion, plastic film {RER}| production | Alloc Rec, U	0.384615	kg
	Transport, freight, lorry 7.5-16 metric ton, EURO4 {RER}| transport, freight, lorry 7.5-16 metric ton, EURO4 | Alloc Rec, U	250	tkm
	Diesel, at regional storage/RER U	203.4615	kg
	Electricity, medium voltage, production CH, at grid/CH U	33.84615	kWh
Reed mats	Reed production {CH}| production | Alloc Def, U	0.91	kg
	Steel, unalloyed {RER}| steel production, converter, unalloyed | Alloc Def, U	0.09	kg
	Transport, freight, lorry 16-32 metric ton, EURO3 {RER}| transport, freight, lorry 16-32 metric ton, EURO3 | Alloc Def, U	0.05	tkm
	Transport, freight, lorry 16-32 metric ton, EURO3 {RER}| transport, freight, lorry 16-32 metric ton, EURO3 | Alloc Def, U	0.75	tkm
	Industrial machine, heavy, unspecified {GLO}| market for | Alloc Def, U	0.0015	kg
	Electricity, medium voltage {AT}| market for | Alloc Def, U	0.01388	kWh
Straw	Straw, stand-alone production {CH}| production | Alloc Def, U	1	kg
	Chipper, stationary, electric {RER}| production | Alloc Def, U	0.0000002	p
	Electricity, medium voltage {CH}| market for | Alloc Def, U	0.00166	kWh
	Transport, freight, lorry 16-32 metric ton, EURO3 {RER}| transport, freight, lorry 16-32 metric ton, EURO3 | Alloc Def, U	0.05	tkm

Table S 2 – Ecoinvent Processes used for the GWP calculation of each material

1.4. Replacement and service life of construction materials

To address the replacement of building assemblies and components, we defined the service lives for the identified building elements. The building service life is assumed to be 60 years[13]. The following table (Table S 3) provides the median service life, based on the Swiss Federal Office for Energy ‘DUREE’ research project [14] and Heeren et al. 2015 [15]. According to these references, the structural elements service-life corresponds to the building’s one, i.e., 60 years, together with the waterproof membrane in polyethylene. All the finishing, windows and window frames have a service life of 30 years [14]. Regarding the biobased insulation, 60 years were chosen as suggested from Goswein et al. 2021 [16]. As a matter of fact, thanks to the replacement of the finishing and good installation practices, it is possible to maintain the breathability of the fibrous insulation materials while eliminating the direct contact with the water; moreover, it is possible to reach the 60 years for the service life and maximize the carbon storage in the building skins.

Element	***Service Life [years]***	***REFERENCE***
**Insulated Triple Glazing**	30	14
**Wood-Aluminum Window Frame**	30	14
**PVC Window Frame**	30	14
**Waterproof membrane Polyethylene**	60	15
**Wood Window Frame**	30	14
**Steel (reinforcement)**	60	15
**Mineral plaster**	30	14
**Ceramic tiles**	30	14
**Cross Laminated Timber (CLT)**	60	15
**Glulam (GLT)**	60	15
**OSB**	60	15
**Gypsum plasterboard**	30	14
**Concrete C30/37**	60	15
**Concrete C25/30**	60	15
**Concrete deep foundations**	60	15
**Solid wood (softwood)**	60	14

Clay plaster	30	[14]
Bamboo Cladding	30	[14]
Bamboo Flooring	30	[14]
Solid wood (hardwood)	30	[14,15]
Glue Laminated Bamboo (GLB)	60	[15]
Cross Laminated Bamboo (CLB)	60	[15]
Hemp fiber	60	[16]
Reed mats	60	[16]
Straw	60	[16]

Table S 3 – Service life of construction materials used, expressed in years.

1.5. GWP $_{net}$ calculation considering 100 years' time horizon

To quantify all CO_{2eq} emissions, we performed a dynamic LCA for all construction materials.
As illustrated in Figure S 9, different GWP calculation logics were used for the biogenic and fossil emissions. The strategies were defined according to the type of material (biobased or not) and if the element is to be replaced after 30 years. As a matter of fact, the temporal dynamics of emissions and the spatial dynamics of the building elements' replacement must be considered from the year 31 to the end of life of the building. During the building life of 60 years, GHGs are released and generate an impact on the climate. By choosing a time horizon of 100 years, one might think that this LCA study focuses on the global warming impacts over 100 years. However, the emissions occurring after 30 years, e.g., for the replacement of building elements, are considered from year 31 to year 130. Therefore, to be coherent with the 100-year time analysis, there is the need to use a dynamic approach able to assess the impacts on the same temporal frames, as explained from Levasseur and coauthors [17].
When the materials are produced at year 1 , the dynamic method can be simplified with tabulated values, such as the GWP as defined by IPCC 2013 method (GWP_{100}), and the CO_2 uptake from biogenic regeneration in the land ($GWP_{bio\ index}$) with the Guest et coauthors simplified tabulated dynamic index (also considered as semi-static) [18]. Whereas when the building elements are replaced after 30 years, both the fossil emissions ($GWP_{100,dyn}$) and the CO_2 uptake ($GWP_{bio\ index,\ dyn\ 31\text{-}60}$) are calculated with the "DynCO2" calculation tool [19].

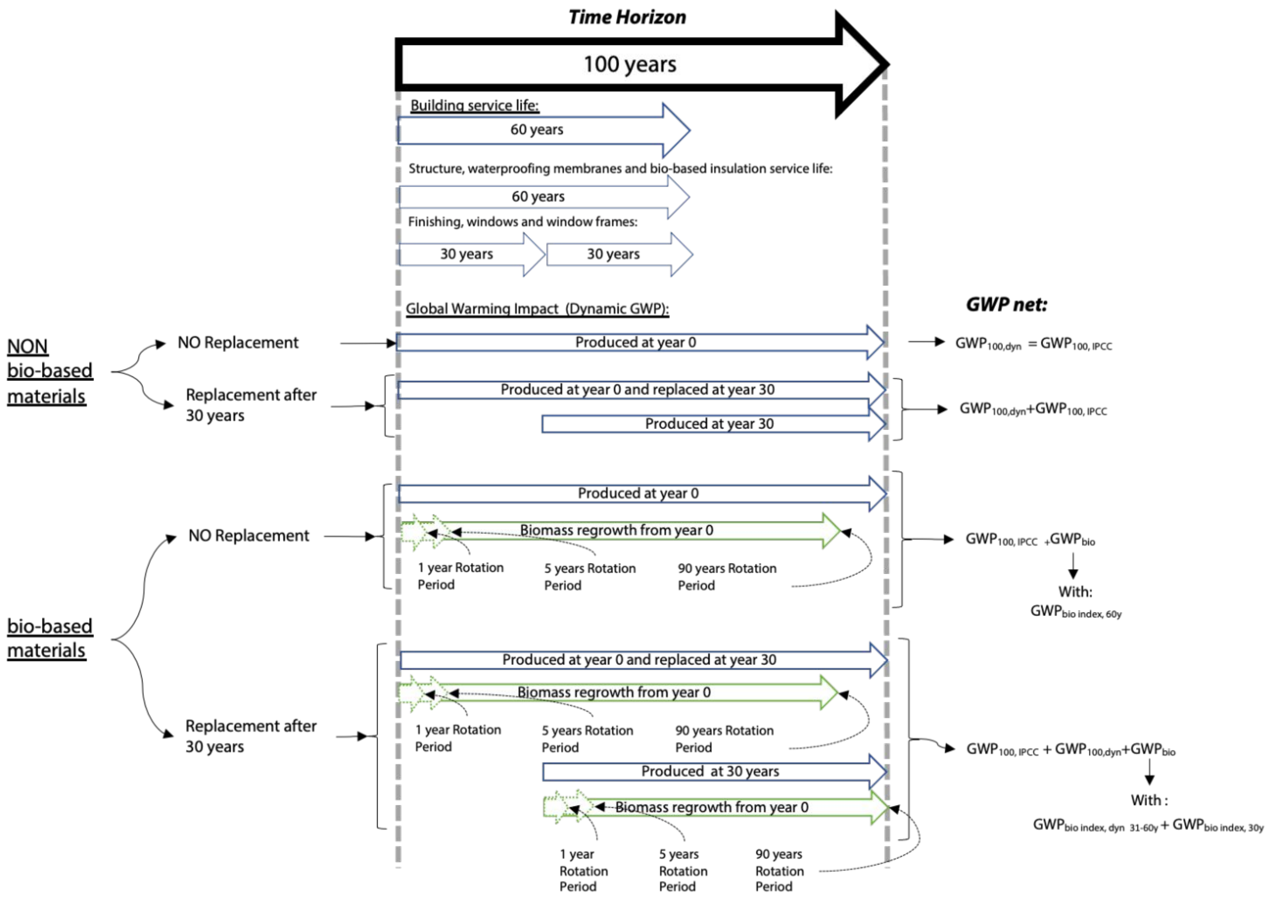

Figure S 9 – Schematization of the GWP index to use according to the type of material (biobased or not) and if the element must be replaced after 30 years.

The final Net-GWP value (GWP_{net}) is equal to Equation (2):

$$\text{GWP}_{net}\ [kg\ CO_{2,eq}/kg] = \text{GWP}_{100} + \text{GWP}_{bio} \quad (2)$$

Where:

- GWP_{100} is the either $GWP_{100,\ IPCC}$ and/or $GWP_{100,\ dyn}$ according to the material production time (see scheme in Figure S 9);
- GWP $_{bio}$ see next subparagraph 1.5.1.

The Net-GWP for every single material used in this study, which is the most important output of this materials section, has been illustrated in Table S 4, both per mass and volumetric values.

Materials	Service life	λ	T	ρ_0	CC	BC	CO_2 storage	GWP 100, IPCC	GWP 100,dyn	GWP_{bio} index, 60 y	GWP bio index, 30y	GWP_{bio} index, dyn 31-60	GWP bio	Net-value	Net-value	Ref
Family	years	[W/ m•K]	[m]	[kg/ m³]	[%]	[%]	kg CO_2/ kg	[kg CO_{2eq}/ kg]	[kg CO_{2eq}/ kg]	[kg CO_{2eq}/ $kgCO_2$]	[kg CO_{2eq}/ $kgCO_2$]	[kg CO_{2eq}/ $kgCO_2$]	[kg CO_{2eq}/ kg]	[kg CO_{2eq}/ kg]	[kg CO_{2eq}/ m³]	/
Insulated Triple Glazing	30	/	0.04	30.00	0%	0%	0.0	52.58	47.31	0.00	0.00	0.00	0.00	99.89	2996.75	9,10
Wood-Aluminum Window Frame	30	/	0.08	1042.50	0%	0%	0.0	4.78	3.65	0.00	0.00	0.00	0.00	8.43	8790.10	9,10
PVC Window Frame	30	/	0.08	1181.25	0%	0%	0.0	4.97	3.80	0.00	0.00	0.00	0.00	8.77	10357.41	9,10
Waterproof membrane Polyethylene	60	/	0.0015	1000.00	0%	0%	0.0	2.70	0.00	0.00	0.00	0.00	0.00	2.70	2699.03	9,10
Wood Window Frame	30	/	0.08	1002.50	0%	0%	0.0	2.37	1.81	0.00	0.00	0.00	0.00	4.18	4191.81	9,10
Steel (reinforcement)	60	/	/	7850.00	0%	0%	0.0	2.22	0.00	0.00	0.00	0.00	0.00	2.22	17421.83	9,10

Mineral plaster	30	0.08	0.02	1100.00	0%	0%	0.0	0.61	0.46	0.00	0.00	0.00	0.00	1.07	1174.82	9,10
Solid wood (softwood)	30	/	0.02	450.21	50%	100%	-1.8	0.27	0.21	-0.12	0.15	0.16	0.57	1.05	472.96	9,10, 20
Ceramic tiles	30	/	0.009	2000.00	0%	0%	0.0	0.49	0.38	0.00	0.00	0.00	0.00	0.87	1746.43	9,10
Cross Laminated Timber (CLT)	60	/	/	426.92	50%	98%	-1.8	0.53	0.00	-0.12	0.15	0.16	-0.22	0.31	133.07	9,10, 20, 21
Glulam (GLT)	60	/	/	426.92	50%	98%	-1.8	0.53	0.00	-0.12	0.15	0.16	-0.22	0.31	133.07	9,10, 20, 21
OSB	60	/	/	549.54	50%	98%	-1.8	0.51	0.00	-0.12	0.15	0.16	-0.22	0.29	159.15	9,10
Gypsum plasterboard	30	0.21	0.0125	850.00	0%	0%	0.0	0.22	0.17	0.00	0.00	0.00	0.00	0.39	328.80	9,10
Concrete C30/37	60	/	/	2300.00	0%	0%	0.0	0.18	0.00	0.00	0.00	0.00	0.00	0.18	415.94	9,10
Concrete C25/30	60	/	/	2300.00	0%	0%	0.0	0.16	0.00	0.00	0.00	0.00	0.00	0.16	359.03	9,10
Concrete deep foundations	60	/	/	2325.00	0%	0%	0.0	0.14	0.00	0.00	0.00	0.00	0.00	0.14	318.79	9,10
Bamboo Cladding	30	0.34	0.02	649.78	54%	97%	-1.9	0.93	0.71	-0.48	-0.22	-0.25	-0.89	0.75	488.22	22, 23, 10
Bamboo Flooring	30	/	0.015	649.78	54%	97%	-1.9	0.93	0.71	-0.48	-0.22	-0.25	-0.89	0.75	488.22	9,10, 20, 21
Solid wood (hardwood) finishing	30	0.12	0.02	640.38	50%	100%	-1.8	0.08	0.06	/	0.15	/	0.28	0.41	264.28	9,10, 20, 21
Clay plaster	30	0.47	0.02	1800.00	0%	0%	0.0	0.05	0.04	0.00	0.00	0.00	0.00	0.09	169.80	9,10
Solid wood (hardwood) structural	60	0.12	0.02	640.38	50%	100%	-1.8	0.08	0.00	-0.12	0.15	0.16	-0.22	-0.14	-91.41	9,10, 20, 21
Glue Laminated Bamboo (GLB)	60	/	/	635.83	54%	97%	-1.9	0.77	0.00	-0.48	-0.22	-0.25	-0.92	-0.15	-95.51	22, 23, 10
Cross Laminated Bamboo (CLB)	60	/	/	635.83	54%	98%	-1.9	0.77	0.00	-0.48	-0.22	-0.25	-0.93	-0.16	-101.56	22, 23, 10
Hemp fiber	60	0.049	/	85.50	45%	65%	-1.1	0.10	0.00	-0.50	-0.23	-0.26	-0.54	-0.44	-37.52	7, 24, 10
Reed mats	60	0.050	/	180.50	47%	98%	-1.7	0.38	0.00	-0.50	-0.23	-0.26	-0.85	-0.46	-83.24	7, 25, 10
Straw	60	0.052	/	95.00	40%	100%	-1.5	0.14	0.00	-0.50	-0.23	-0.26	-0.73	-0.60	-56.56	7, 24, 10

Table S 4 – Properties of construction materials used. In particular: Service life, Thermal conductivity (λ), thickness (T), dry density (ρ_0), Carbon Content (CC), Biomass Content (BC), CO_2 Storage ($CO_{2\ Storage}$), Global Worming Potential at 100 years ($GWP_{100,IPCC}$), Global Warming Potential at 100 years calculated with the dynamic LCA from year 31 to year 100 ($GWP_{100,dyn}$), Global Warming potentials bio indexes calculated with the Guest et al. method [18] with a storage periods of 60 ($GWP_{bio\ index,\ 60y}$) and 30 years ($GWP_{bio\ index,\ 30y}$), Global Warming potentials bio indexes calculated with the dynamic tool for the replaced elements from year 31 to 60 years ($GWP_{bio\ index,\ dyn\ 31-\ 60y}$), Global Warming Potential considering the biogenic portion (GWP_{bio}), Net-GWP value expressed per kg or m^3. Materials highlighted in red are High-Carbon, in yellow are Low-Carbon and in green Climate-negative.

Non-biobased materials do not contribute to carbon storage or uptake, therefore their Net-GWP values are always positive, as equivalent to GWP_{100} (IPCC or Dynamic) value. Contrary, every biobased material used in construction can account for a removal potential and, depending on their carbon fossil emissions and their storage and rotation period, their Net-GWP values can be either negative or positive.

1.5.1. GWP $_{bio}$ calculation considering 100 years' time horizon

In the literature there is no consensus on how to model biogenic carbon released and reabsorbed during biobased materials' life cycle [26]. The established approaches can be summarized as the 0/0, +1/-1, the dynamic (with carbon uptake before or after construction). Assuming a sustainable supply of biomass, in traditional Life-Cycle-Assessment [27] (LCA), the biogenic cycle is usually considered neutral as the carbon used in construction is sequestered in new plants in the natural system (0/0). In fact, if timing is excluded from the analysis, the carbon-neutrality of biobased product correspond to their climate-neutrality. This widely used assumption has been progressively criticized by some researchers showed that the carbon cycle is in fact not neutral [17,28]. The +1/-1 approaches, such as the British Publicly Available Specification – PAS 2050 [29] and the European Commission's ILCD Handbook [30], tried to address this issue by tracking the biogenic carbon flows over the building life-cycle. However, these "static" models are still not able to consider the impact of timing of the carbon emissions and its influence of the rotation period related to the biomass growth. Therefore, the dynamic approach (DLCA) was developed [17,28] with two uptake scenarios (before or after the construction) leading to radically different results [31]. The latter has the advantage to have the same time frame as fossil emissions as the identical time 0 is assumed (time of production/construction). The dynamic methods are particularly relevant for biobased products that can store carbon and delay emissions. Specifically, wooden products have a longer

rotation period related to the slow growth of forests; therefore, they cannot be considered climate-neutral when a short time horizon is chosen. Contrariwise, fast-growing biobased materials, as straw or bamboo, are able to fully regrow in a shorter rotation period, providing an effective mitigation effect on GHG emissions [32]. Cherubini and coauthors [33] pushed this concept by determining a specific time-depending characterization factors for biogenic carbon dioxide, with the inclusion of the rotation period of plants. As a continuation, Guest and coauthors [34] further expanded this method by proposing an index, the biogenic global warming potential index ($GWP_{bio\ index}$), which is able to directly compute the carbon dioxide regeneration with the biogenic CO_2 pulse emissions, both acting as a perturbation to the atmospheric CO_2 decay according to the Bern CO_2 declining curve. Indeed, this index is capable to consider the storage period of harvested biomass with different rotation periods in the anthroposphere as a negative value to be considered at the beginning of a standard LCA, both for a100 or 500-year time horizon, in a semi-static way. Biobased materials can thus help decreasing the GWP by uptaking the CO_2 and keep it stored in a construction product for a long period. More precisely, the biomass is stored in the anthroposphere as a harvested product, e.g. solid wood, while the carbon uptake happens in the biomass that is regrowing through the photosynthesis, reducing the atmospheric carbon dioxide concentration. To account for this biogenic CO_2 storage in the anthroposphere, in two cases we used the semi-static tabulated values proposed by Guest and coauthors [18] and depicted in Figure S10. They defined a GWP_{bio} index for considering the consequential GWP of storing 1 kg of biogenic CO_2 for a given storage period in a 100 years' time horizon. Thus, the method combines through a Dynamic LCA (DLCA) the annual CO_2 uptake in the land via biomass growth and the delayed biogenic CO_2 emissions through biomass incineration at end of life of a building, here assumed equal to 60 years [13]. The $GWP_{bio\ index}$ can assume a positive value if the storage period is short and rotation long, while can reach negative values, up to -1 $kgCO_{2eq}$, for long storage and very short rotation periods. Hence, to remove from the atmosphere the equal amount of carbon that is stored in biobased products, fast-growing spices need a shorter time than slow-growing ones, resulting in a more advantageous effect in lowering the radiative force remaining in the atmosphere in a short period. In this work, the storage period in the anthroposphere was assumed to be 60 years for structural elements, waterproof membrane in polyethylene and biobased insulation materials. For the finishing, windows and window frames, the storage period is of 30 years (Table S 3).

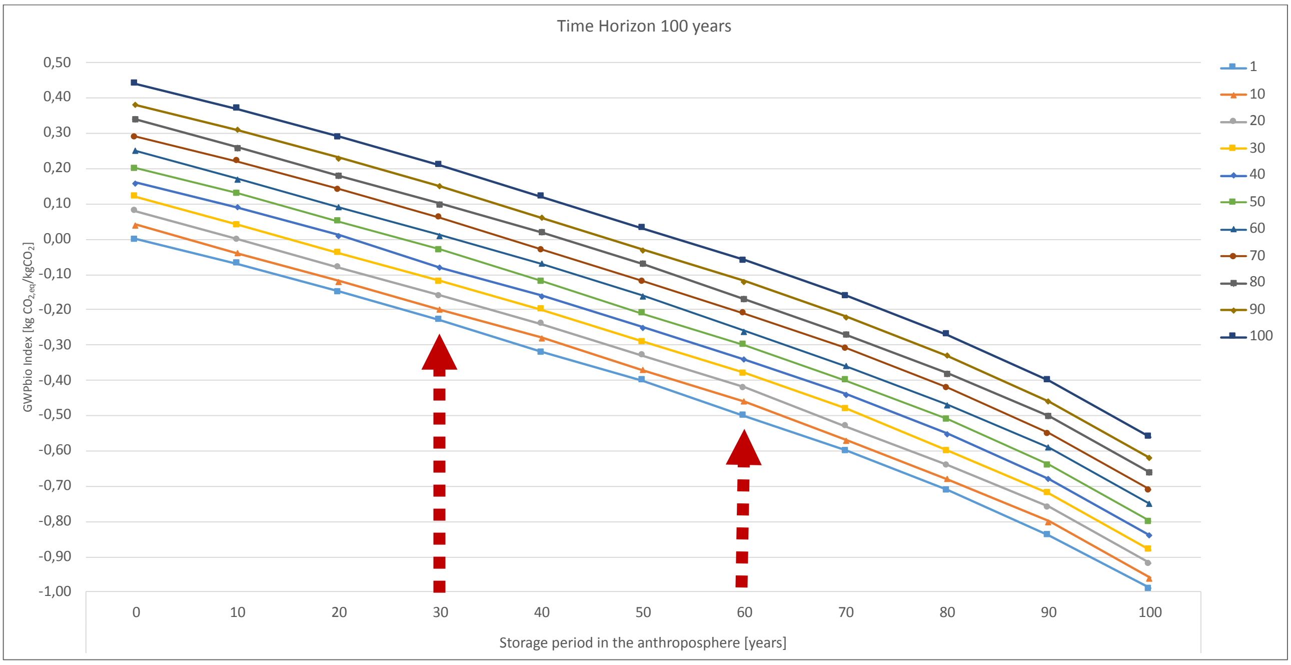

Figure S 10 – Each color refers to the rotation period of the different biomass with time horizon 100 years. The red arrows highlight the storage period take in consideration in this research, namely 30 years for replaced element and 60 years for the ones that are kept up to the end of life of the building. Source: Guest et al [18]

For the replacing elements inserted on the building after 30 years, a particular index is computed with the use of the dynamic LCA ($GWP_{bio\ index,\ dyn\ 31\text{-}60y}$), as the possibility to address the timing of

emissions and sequestration related to these boundaries with a Dynamic LCA is the most robust and transparent solution[35,36]. This dynamic $GWP_{bio\ index\ dyn\ 31\text{-}60y}$ has been calculated with the "DynCO2" calculation tool [19].
For the element inserted at year 1, by using Figure S 10, we extract the GWP_{bio} for every biobased material by entering in the graph at 60 and 30 years and extracting the relative $GWP_{bio\ index,60y}$ and $GWP_{bio\ index,30y}$ indexes for the different biomass according to their rotation period.
In particular:

- Solid wood with a rotation period of 90 years,
- Bamboo with a 5-year rotation,
- straw, reed and hemp biomass have a fast rotation period of 1 year. The $GWP_{bio\ index,30y}$ and the dynamic ones have not been considered because no fast-growing elements is replaced after 30 years.

The Table S 5 summarizes the indexes described.

Rotation period	GWP bio index, 60y	GWP bio index, 30y	GWP bio index, dyn 31-60y
1	-0.5	-0.23	-0.26
5	-0.48	-0.22	-0.246
90	-0.12	0.15	0.16

Table S 5 –$GWP_{bio\ index}$, expressed in [kg CO_{2eq}/kgCO_2].

To calculate the carbon sequestration of biobased materials, the following Equation (3) is considered, which calculate the mass of CO_2 that can be stored in the final product:

$$\mathrm{CO}_{2,storage} = \rho_0 \cdot CC \cdot BC \cdot 3.67 \qquad [kg\ CO_2/kg] \qquad (3)$$

Where:

- ρ_0 is the dry density of the material, in kg/m^3;
- CC is the carbon content of the biogenic material;
- BC the biomass content of the finished product;
- 3.67 is the molar weight ratio between CO_2 and C [37].

Since the exact moisture content of biogenic materials is often unknown, we supposed a 20% moisture content for structural materials, 15% for finishing and membrane, and 10% for isolations. Therefore, we calculated the dry volumetric mass according to the CEN/TC 124 [38], as reported in the following Equation (4):

$$\rho_0 = \rho_{\omega < 25} \cdot \frac{100 + 0.45 \cdot \omega}{100 + \omega} \left[kg \cdot m^{-3}\right] \qquad (4)$$

where:

- $\rho_{\omega < 25}$ is the wood density at moisture content lower than 25%, in kg/m^3;
- ω is the moisture content, in %.

Consequently, as reported in Equation (5), the contribution on GWP from carbon uptake can be calculated by multiplying the CO_2 storage with the $GWP_{bio\ index}$ expressed in [kg CO_{2eq} /kgCO_2] , which is a portion of the total carbon storage a material could reabsorb in the land during the storage period in 100 years of time horizon:

$$GWP_{bio} = GWP_{bio\ index} \cdot CO_{2,storage} \qquad [kg\ CO_{2eq}/kg] \qquad (5)$$

Where:

- $GWP_{bio\ index}$ is the either $GWP_{bio\ index,\ 60y}$, $GWP_{bio\ index,\ 30y}$ or $GWP_{bio\ index,\ dyn\ 31\text{-}60y}$, according to the situation (see scheme in Figure S 9)

1.6. U-value Calculation

The general formula for calculating the U-Value is (Equation (6)):

$$U\left[\frac{W}{m^2\,K}\right] = \frac{1}{R_t\left[\frac{m^2\,K}{W}\right]} \qquad (6)$$

Where:
U = Thermal Transmittance
R_t = Total Thermal Resistance of the element composed of layers, obtained according to Equation (7):

$$R_t = R_{si} + R_1 + R_2 + \ldots + R_n + R_{se} \qquad (7)$$

Where:
R_{si} = Interior Surface Thermal Resistance, here assumed as 8 W/(m^2K)
R_{se} = Exterior Surface Thermal Resistance, here assumed as 25 W/(m^2K)

R_1, R_2, R_3, R_n = Thermal Resistance of each layer, which is obtained according to Equation (8):

$$R_n\left[\frac{m^2\,K}{W}\right] = \frac{D\,[m]}{\lambda\left[\frac{W}{K\,m}\right]} \qquad (8)$$

Where:
- D = Material Thickness (m)
- λ = Thermal Conductivity of the Material (W/ K·m) (according to each material).

In particular:
- Mineral plaster (0.02 m thickness) : 0.08 W/(m K)[9]
- Solid Wood (0.02 m thickness) : 0.12 W/(m K) [9]
- Plasterboards (0.0125 m thickness) : 0.21 W/(m K) [9]
- Clay Panels (0.02 m thickness) : 0.47 W/(m K) [9]
- Bamboo cladding (0.02 m thickness) : 0.34 W/(m K) [37]
- Reed mats (varying thickness) : 0.050 W/(m K) [7]
- Hemp fibers (varying thickness) : 0.049 W/(m K) [7]
- Straw (varying thickness) : 0.052 W/(m K) [7]

1.7. Insulation line-load calculation

Once computed the different wall biobased insulation thicknesses and their related thermal performance, it is possible to calculate the corresponding line-load on the structure to control that it does not exceed the one considered during the structural dimensioning for different material diets (i.e., 2,98 kN/m, in paragraph SI 1.2.4).
According to Equation (9), we checked the load limits for each insulation and material diet as follow:

$$\mathrm{I}_{pl,i} = \rho \cdot \mathrm{D} \cdot H\,floor \cdot g \cdot 10^{-3} < \mathrm{I}_{pl} = 2.98 \quad [kN/ml] \qquad (9)$$

where:

- D = Material Thickness (m) ;
- ρ = density of the biobased insulation material (kg/m^3) (reed mats, hemp fibers or straw);
- g = gravity on Earth = $9.8\,\frac{m}{s^2}$;
- 10^{-3} is the conversion factor from N to kN.

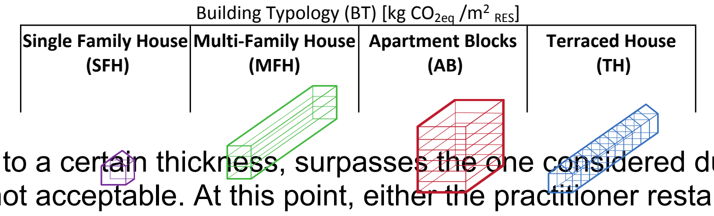

When the resulting insulation load, corresponding to a certain thickness, surpasses the one considered during the pre-dimensioning, this solution is not acceptable. At this point, either the practitioner restarts the process by increasing the insulation dead loads (with the consequent increasement of structural material quantities and emissions for the diets) or simply chose among the acceptable solutions determined for other diets or with a different insulation material that suit its architectural needs.

2. Extended Results

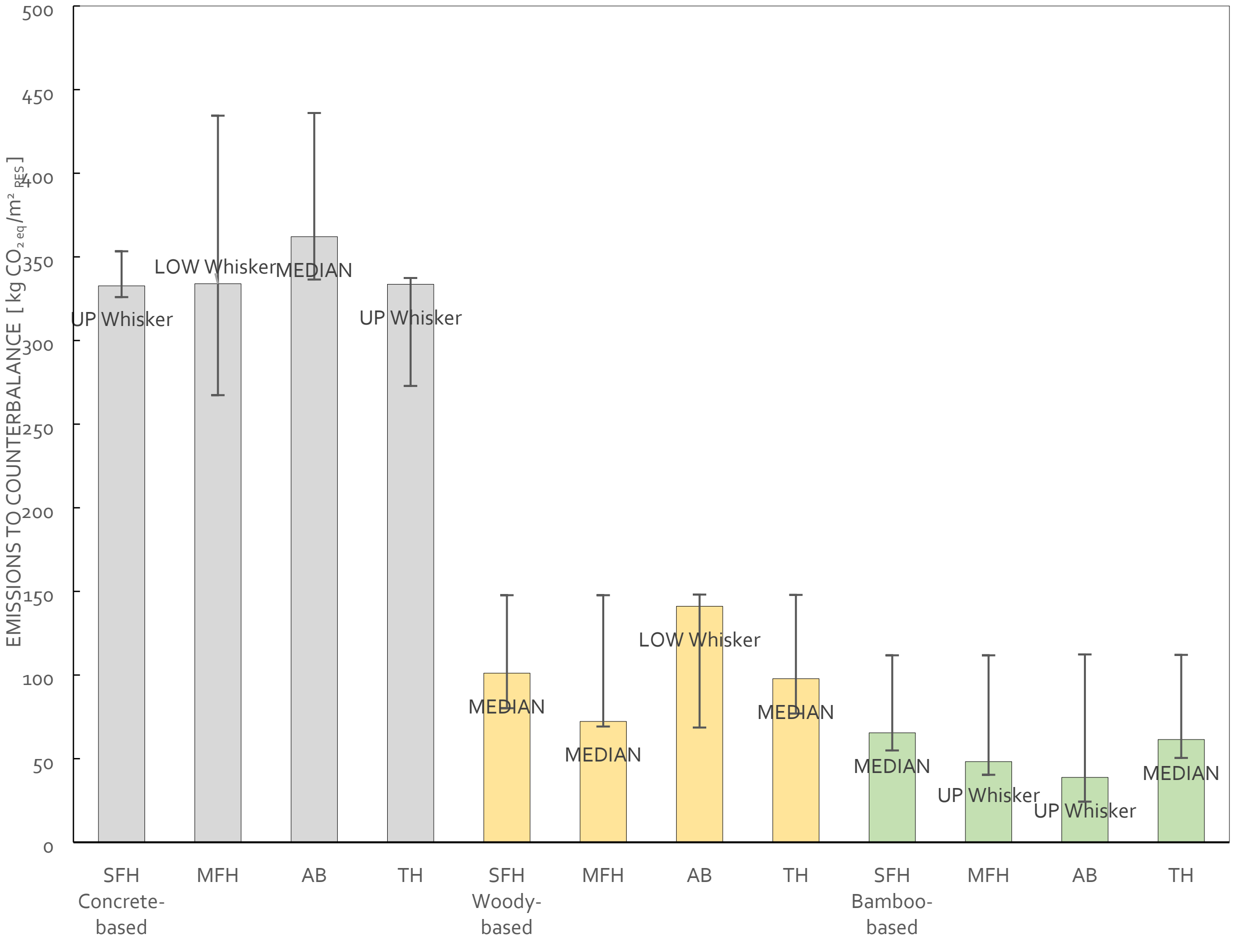

Figure S 11 – Representation of the material diets' emissions to counterbalance for the three geometrical configurations and the four BT

	Element	Material	Net-GWP [kg CO_{2eq} /kg]	CEM	TIM	BAM	CEM	TIM	BAM	CEM	TIM	BAM	CEM	TIM	BAM
UP Whisker	*Above ground Structure (AGS)*	Concrete 25/30	0.16	86.17			86.17			86.17			86.17		
		Concrete 30/37	0.18	16.74			25.99			37.59			16.74		
		Steel	2.22	48.82			52.70			57.56			48.82		
		Solid Wood (softwood)	-0.14		-10.20			-12.97						-10.20	
		Wood OSB	0.29		8.30			8.30						8.30	
		Wood CLT	0.31								24.94				
		Wood Glulam	0.31								23.11				
		Cross Laminated Bamboo	-0.16			-28.85			-28.85			-17.89			-28.85
		Glued Laminated Bamboo	-0.15									-15.37			
	Underground structure (UGS)	Concrete 25/30	0.16	17.85	9.34	10.69	14.16	8.18	11.29	8.09	5.27	3.85	17.85	9.34	10.69
		Steel	2.22	25.98	13.59	15.56	98.64	11.91	16.44	128.82	42.35	5.61	25.98	13.59	15.56
	Windows (W)	Insulated Triple Glazing	99.89	18.34	16.18	16.18	18.34	17.26	17.26	18.34	18.34	18.34	18.34	16.18	16.18
		PVC frame	8.77	14.09		0.00	14.09			14.09			14.09		
		Wood aluminum frame	8.43		10.55	0.00		11.25			11.95			10.55	
		Wood frame	4.18			5.03			5.37			5.70			5.03
	Waterproofing membrane (WM)	Polyethylene	2.70	2.02	2.02	2.02	1.01	1.01	1.01	0.58	0.58	0.58	2.02	2.02	2.02
	Finishing (Fi)	Solid wood (hardwood)	0.41		9.46			6.03			5.13			7.66	
		Solid wood (softwood)	1.05		9.46			9.46			9.46			9.46	
		Ceramic tiles	0.87	15.72			15.72			15.72			15.72		
		Mineral plaster	1.07	107.61			107.61			69.08			91.64		
		Gypsum plasterboard	0.39		11.47	0.00		8.80						10.07	
		Bamboo Flooring	0.75			7.32			7.32			7.32			7.32
		Clay plaster	0.09			9.47			7.27			6.69			8.32
		Bamboo Cladding	0.75			17.48			11.13			9.47			14.16
	EMISSIONS TO COINTERBALANCE			353.35	80.17	54.92	434.42	69.22	48.25	436.02	141.12	24.30	337.37	76.98	50.44
	Above ground Structure (AGS)	Concrete 25/30	0.16	86.17			86.17			86.17			86.17		
		Concrete 30/37	0.18	8.24			8.24			8.24			8.24		
		Steel	2.22	45.26			45.26			45.26			45.26		
		Solid Wood (softwood)	-0.14		-8.12			-8.12			-8.12			-8.12	
		Wood OSB	0.29		8.30			8.30			8.30			8.30	
		Wood CLT	0.31												
		Wood Glulam	0.31												
		Cross Laminated Bamboo	-0.16			-28.85			-28.85			-28.85			-28.85
		Glued Laminated Bamboo	-0.15												
	Underground structure (UGS)	Concrete 25/30	0.16	38.10	37.35	37.35	38.10	37.35	37.35	38.10	37.35	37.35	38.10	37.35	37.35

	Element	Material													
		Steel	2.22	55.46	54.38	54.38	55.46	54.38	54.38	55.46	54.38	54.38	55.46	54.38	54.38
	Windows (W)	Insulated Triple Glazing	99.89	17.26	18.34	18.34	17.98	18.34	18.34	18.34	18.34	18.34	17.26	18.34	18.34
		PVC frame	8.77	13.26			13.81	0.00		14.09			13.26		
		Wood aluminum frame	8.43		11.50			11.50			11.50			11.50	
		Wood frame	4.18			5.70			5.70			5.70			5.70
	Finishing (Fi)	Solid wood (hardwood)	0.41		2.43			2.43			2.64			2.54	
		Solid wood (softwood)	1.05		9.46			9.46			9.46			9.46	
		Ceramic tiles	0.87	15.72			15.72			15.72			15.72		
		Mineral plaster	1.07	45.11			45.11			46.99			46.05		
		Gypsum plasterboard	0.39		6.00			6.00			6.17			6.08	
		Bamboo Flooring	0.75			7.32			7.32			7.32			7.32
		Clay plaster	0.09			4.96			4.96			5.09			5.03
		Bamboo Cladding	0.75			4.49			4.49			4.88			4.69
	Waterproofing membrane (WM)	Polyethylene	2.70	8.10	8.10	8.10	8.10	8.10	8.10	8.10	8.10	8.10	8.10	8.10	8.10
	EMISSIONS TO COINTERBALANCE			332.67	147.73	111.79	333.94	147.73	111.79	336.46	148.11	112.32	333.61	147.92	112.06
MEDIAN	***Above ground Structure (AGS)***	Concrete 25/30	0.16	86.17			86.17			86.17			86.17		
		Concrete 30/37	0.18	10.47			16.74			19.16			10.47		
		Steel	2.22	46.20			48.82			49.84			46.20		
		Solid Wood (softwood)	-0.14		-8.82			-10.20			-10.89			-8.82	
		Wood OSB	0.29		8.30			8.30			8.30			8.30	
		Wood CLT	0.31												
		Wood Glulam	0.31												
		Cross Laminated Bamboo	-0.16			-28.85			-28.85			-28.85			-28.85
		Glued Laminated Bamboo	-0.15												
	Underground structure (UGS)	Concrete 25/30	0.16	23.67	18.68	18.68	17.85	9.34	10.69	22.65	8.17	10.15	23.67	18.68	18.68
		Steel	2.22	34.46	27.19	27.19	25.98	13.59	15.56	111.00	11.90	14.78	34.46	27.19	27.19
	Windows (W)	Insulated Triple Glazing	105.16	16.18	17.26	17.26	17.26	17.98	17.98	18.34	18.34	18.34	16.18	17.26	17.26
		PVC frame	9.94	12.43	0.00		13.26			14.09	0.00		12.43		
		Wood aluminum frame	9.56		10.82			11.27			11.50			10.82	
		Wood frame	4.74			5.37			5.59			5.70			5.37
	Waterproofing membrane (WM)	Polyethylene	2.70	4.05	4.05	4.05	2.02	2.02	2.02	1.62	1.62	1.62	4.05	4.05	4.05
	Finishing (Fi)	Solid wood (hardwood)	0.43		5.97			3.59			3.44			3.81	
		Solid wood (softwood)	0.84		9.46			9.46			9.46			9.46	
		Ceramic tiles	0.99	15.72			15.72			15.72			15.72		
		Mineral plaster	1.22	76.60			23.50			23.50			23.50		
		Gypsum plasterboard	0.43		8.22			6.90			6.78			7.07	
		Bamboo Flooring	0.97			7.32			7.32			7.32			7.32
		Clay plaster	0.11			3.40			3.40			3.40			3.40
		Bamboo Cladding	0.97			11.03			6.64			6.35			7.03
	EMISSIONS TO COINTERBALANCE			325.95	101.13	65.45	267.33	72.27	40.36	362.08	68.61	38.81	272.85	97.81	61.44

Cement-based | Timber-based | Bamboo-based

Table S 6 – Material diets' emissions to counterbalance for the three geometrical configurations and the four BT

			Climate Positive $[m^3/m^2_{RES}]$	*Climate Negative $[m^3/m^2_{RES}]$*	*MDI*	*Climate Positive $[m^3/m^2_{RES}]$*	*Climate Negative $[m^3/m^2_{RES}]$*	*MDI*	*Climate Positive $[m^3/m^2_{RES}]$*	*Climate Negative $[m^3/m^2_{RES}]$*	*MDI*
Reed mats (max)	**SFH**	UP Whisker	0.44	4.25	9.58	0.12	1.13	9.33	0.41	0.67	1.63
		LOW Whisker	0.43	4.00	9.31	0.19	1.89	10.07	0.44	1.36	3.09
		MEDIAN	0.42	3.92	9.37	0.16	1.35	8.24	0.39	0.80	2.06
	**MFH**	UP Whisker	0.46	5.22	11.37	0.11	1.02	9.26	0.39	0.59	1.53
		LOW Whisker	0.43	4.01	9.34	0.19	1.89	10.05	0.44	0.50	1.14
		MEDIAN	0.37	3.21	8.66	0.10	1.01	9.72	0.36	0.50	1.40
	**AB**	UP Whisker	0.44	5.24	11.93	0.41	1.73	4.23	0.41	0.31	0.74
		LOW Whisker	0.43	4.04	9.37	0.19	1.88	9.97	0.44	1.36	3.09
		MEDIAN	0.40	4.35	11.00	0.10	0.98	9.37	0.35	0.48	1.36
	**TH**	UP Whisker	0.43	4.05	9.44	0.12	1.09	9.28	0.40	0.62	1.55
		LOW Whisker	0.43	4.01	9.32	0.19	1.90	10.08	0.44	1.36	3.09
		MEDIAN	0.37	3.28	8.79	0.14	1.31	9.62	0.38	0.75	1.98
Hemp fiber (min)	**SFH**	UP Whisker	0.44	9.42	21.25	0.12	2.30	19.01	0.41	1.48	3.57
		LOW Whisker	0.43	8.87	20.66	0.19	4.06	21.57	0.44	2.99	6.80
		MEDIAN	0.42	8.69	20.78	0.16	2.83	17.26	0.39	1.76	4.53
	**MFH**	UP Whisker	0.46	11.58	25.22	0.11	2.03	18.49	0.39	1.30	3.34
		LOW Whisker	0.43	8.90	20.72	0.19	4.06	21.53	0.44	2.99	6.80
		MEDIAN	0.37	7.12	19.20	0.10	2.07	19.87	0.36	1.09	3.06
	**AB**	UP Whisker	0.44	11.62	26.46	0.41	3.80	9.26	0.41	0.66	1.60
		LOW Whisker	0.43	8.97	20.80	0.19	4.05	21.48	0.44	3.01	6.81
		MEDIAN	0.40	9.65	24.40	0.10	1.98	19.00	0.35	1.05	2.96
	**TH**	UP Whisker	0.43	8.99	20.93	0.12	2.21	18.92	0.40	1.36	3.40
		LOW Whisker	0.43	8.89	20.68	0.19	4.06	21.59	0.44	3.00	6.81
		MEDIAN	0.37	7.27	19.50	0.14	2.74	20.17	0.38	1.65	4.34
Straw (med)	**SFH**	UP Whisker	0.44	6.25	14.10	0.12	1.58	13.08	0.41	0.99	2.38
		LOW Whisker	0.43	5.88	13.71	0.19	2.73	14.52	0.44	1.99	4.53
		MEDIAN	0.42	5.76	13.78	0.16	1.93	11.73	0.39	1.17	3.01
	**MFH**	UP Whisker	0.46	7.68	16.73	0.11	1.41	12.83	0.39	0.87	2.23
		LOW Whisker	0.43	5.90	13.75	0.19	2.73	14.49	0.44	1.99	4.53
		MEDIAN	0.37	4.73	12.74	0.10	1.42	13.65	0.36	0.73	2.04
	**AB**	UP Whisker	0.44	7.71	17.56	0.41	2.53	6.17	0.41	0.44	1.07
		LOW Whisker	0.43	5.95	13.80	0.19	2.72	14.43	0.44	2.00	4.53
		MEDIAN	0.40	6.40	16.19	0.10	1.37	13.09	0.35	0.70	1.98
	**TH**	UP Whisker	0.43	5.96	13.89	0.12	1.52	13.01	0.40	0.91	2.27
		LOW Whisker	0.43	5.90	13.72	0.19	2.73	14.53	0.44	2.00	4.53
		MEDIAN	0.37	4.82	12.94	0.14	1.86	13.70	0.38	1.10	2.89

Table S 7 – MDI (Material Diet Index), namely ratios among Climate Negative and Climate Positive materials

			**Single Family House (SFH)** 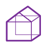			**Multi-Family House (MFH)** 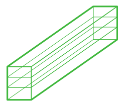			**Apartment Blocks (AB)** 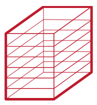			**Terraced House (TH)** 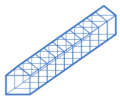		
			**CEM**	**TIM**	**BAM**	**CEM**	**TIM**	**BAM**	**CEM**	**TIM**	**BAM**	**CEM**	**TIM**	**BAM**
UP Whisker	***Structural Scheme***	***Structural Scheme***	RC	PTF	G-xlam	RC	PTF	G-xlam	RC	BF	BF	RC	PTF	G-xlam
	Above Ground Structure (AGS)	Concrete C25/30 $[kg/m^2_{RES}]$	552.00			552.00			552.00			552.00		
		Concrete C30/37 $[kg/m^2_{RES}]$	92.58			143.74			207.85			92.58		

		Steel [kg/m² $_{RES}$]	22.00			23.75			25.93			22.00		
		Solid Wood [kg/m² $_{RES}$]		71.47			90.87						71.47	
		Wood OSB [kg/m² $_{RES}$]		28.66			28.66						28.66	
		Wood CLT [kg/m² $_{RES}$]								80.00				
		Wood Glulam [kg/m² $_{RES}$]								74.12				
		Cross Laminated Bamboo [kg/m² $_{RES}$]			180.60			180.60			112.00			180.60
		Glued Laminated Bamboo [kg/m² $_{RES}$]									102.35			
	Underground structure (UGS)	Concrete C25/30 [kg/m² $_{RES}$]	114.35	59.82	68.48	90.69	52.40	72.35	51.82	33.75	24.69	114.35	59.82	68.48
		Steel [kg/m² $_{RES}$]	11.71	6.13	7.01	44.44	5.37	7.41	58.04	19.08	2.53	11.71	6.13	7.01
	Windows	Insulated Triple Glazing [kg/m² $_{RES}$]	0.18	0.16	0.16	0.18	0.17	0.17	0.18	0.18	0.18	0.18	0.16	0.16
		PVC frame [kg/m² $_{RES}$]	1.61			1.61			1.61			1.61		
		Wood aluminum frame [kg/m² $_{RES}$]		1.25			1.33			1.42			1.25	
		Wood frame [kg/m² $_{RES}$]			1.20			1.28			1.36			1.20
	Waterproofing membrane (WM)	Polyethylene [kg/m² $_{RES}$]	0.75	0.75	0.75	0.38	0.38	0.38	0.21	0.21	0.21	0.75	0.75	0.75
	Finishing (Fi)	Solid wood (hardwood) [kg/m² $_{RES}$]		22.93			14.60			12.42			18.57	
		Solid wood (softwood) [kg/m² $_{RES}$]		9.00			9.00			9.00			9.00	
		Ceramic tiles [kg/m³ $_{RES}$]	18.000			18.000			18.000			18.000		
		Mineral plaster [kg/m² $_{RES}$]	100.76			100.76			64.68			85.80		
		Gypsum plasterboard [kg/m² $_{RES}$]		29.64			22.74			20.93			26.03	
		Bamboo Flooring [kg/m² $_{RES}$]			9.75			9.75			9.75			9.75
		Clay plaster [kg/m² $_{RES}$]			100.44			77.04			70.92			88.20
		Bamboo Cladding [kg/m² $_{RES}$]			23.26			14.82			12.61			18.84
	Biobased Insulation (BI)	Reed mats (max)	766.24	173.86	119.09	942.05	150.10	104.62	945.51	306.01	52.70	731.60	166.93	109.39
		Hemp fibers (min)	805.15	182.69	125.14	989.88	157.73	109.94	993.52	321.55	55.38	768.74	175.41	114.94
		Straw (med)	593.46	134.65	92.24	729.63	116.26	81.03	732.31	237.01	40.82	566.63	129.29	84.72
	Geometrical parameters (GP)	RES [m²] (reference for normalization)		255.00			2250.00			7997.00			297.00	
		Number of conditioned storey		4.00			8.00			14.00			4.00	
		Roof area = basement= area single floor [m²/ m² $_{RES}$]		0.25			0.13			0.07			0.25	
		Exterior Wall area [m²/ m² $_{RES}$]		1.79			1.14			0.97			1.45	
		Window area [m²/ m² $_{RES}$]	0.16	0.15	0.16	0.17	0.16	0.17	0.17	0.17	0.17	0.15	0.15	0.15
LOW Whisker	***Structural Scheme***	***Structural Scheme***	RC	PTF	G-xlam	RC	PTF	G-xlam	RC	BF	BF	RC	PTF	G-xlam
	Above Ground Structure (AGS)	Concrete C25/30 [kg/m² $_{RES}$]	552.00			552.00			552.00			552.00		
		Concrete C30/37 [kg/m² $_{RES}$]	45.55			45.55			45.55			45.55		
		Steel [kg/m² $_{RES}$]	20.39			20.39			20.39			20.39		
		Solid Wood [kg/m² $_{RES}$]		56.92			56.92			56.92			56.92	
		Wood OSB [kg/m² $_{RES}$]		28.66			28.66			28.66			28.66	
		Wood CLT [kg/m² $_{RES}$]												
		Wood Glulam [kg/m² $_{RES}$]												
		Cross Laminated Bamboo [kg/m² $_{RES}$]			180.60			180.60			180.60			180.60
		Glued Laminated Bamboo [kg/m² $_{RES}$]												
	Underground structure (UGS)	Concrete C25/30 [kg/m² $_{RES}$]	244.05	239.29	239.29	244.05	239.29	239.29	244.05	239.29	239.29	244.05	239.29	239.29
		Steel [kg/m² $_{RES}$]	24.99	24.50	24.50	24.99	24.50	24.50	24.99	24.50	24.50	24.99	24.50	24.50
	Windows	Insulated Triple Glazing [kg/m² $_{RES}$]	0.17	0.18	0.18	0.18	0.18	0.18	0.18	0.18	0.18	0.17	0.18	0.18
		PVC frame [kg/m² $_{RES}$]	1.51			1.58			1.61			1.51		
		Wood aluminum frame [kg/m² $_{RES}$]		1.36			1.36			1.36			1.36	0.00
		Wood frame [kg/m² $_{RES}$]			1.36			1.36			1.36			1.36
	Waterproofing membrane (WM)	Polyethylene [kg/m² $_{RES}$]	3.00	3.00	3.00	3.00	3.00	3.00	3.00	3.00	3.00	3.00	3.00	3.00
	Finishing (Fi)	Solid wood (hardwood) [kg/m² $_{RES}$]		5.89			5.89			6.40			6.15	
		Solid wood (softwood) [kg/m² $_{RES}$]		9.00			9.00			9.00			9.00	
		Ceramic tiles [kg/m³ $_{RES}$]	18.00			18.00			18.00			18.00		

		Mineral plaster [kg/m² $_{RES}$]	42.24			42.24			44.00			43.12		
		Gypsum plasterboard [kg/m² $_{RES}$]		15.51			15.51			15.94			15.73	
		Bamboo Flooring [kg/m² $_{RES}$]			9.75			9.75			9.75			9.75
		Clay plaster [kg/m² $_{RES}$]			52.56			52.56			54.00			53.28
		Bamboo Cladding [kg/m² $_{RES}$]			5.98			5.98			6.50			6.24
	Biobased Insulation (BI)	Reed mats (max)	721.39	320.35	242.43	724.15	320.35	87.52	729.61	321.17	243.57	723.43	320.76	243.00
		Hemp fibers (min)	758.02	336.62	254.74	760.92	336.62	254.74	766.65	337.48	255.94	760.17	337.05	255.34
		Straw (med)	558.73	248.12	187.76	560.86	248.12	187.76	565.09	248.75	188.65	560.31	248.43	188.20
	Geometrical parameters (GP)	RES [m²] (reference for normalization)		53.00			86.00			93.00			60.00	
		Number of conditioned storey		1.00			1.00			1.00			1.00	
		Roof area = basement= area single floor [m²/ m² $_{RES}$]		1.00		1.00				1.00			1.00	
		Exterior Wall area [m²/ m² $_{RES}$]		0.46		0.46				0.50			0.48	
		Window area [m²/ m² $_{RES}$]	0.17	0.17	0.17	0.17	0.17	0.17	0.17	0.17	0.17	0.17	0.17	0.17
MEDIAN	***Structural Scheme***	***Structural Scheme***	RC	PTF	G-xlam	RC	PTF	G-xlam	RC	BF	BF	RC	PTF	G-xlam
	Above Ground Structure (AGS)	Concrete C25/30 [kg/m² $_{RES}$]	552.00			552.00			552.00			552.00		
		Concrete C30/37 [kg/m² $_{RES}$]	57.91			92.58			105.96			57.91		
		Steel [kg/m² $_{RES}$]	20.82			22.00			22.46			20.82		
		Solid Wood [kg/m² $_{RES}$]		61.77			71.47			76.32			61.77	
		Wood OSB [kg/m² $_{RES}$]		28.66			28.66			28.66			28.66	
		Wood CLT [kg/m² $_{RES}$]												
		Wood Glulam [kg/m² $_{RES}$]												
		Cross Laminated Bamboo [kg/m² $_{RES}$]			180.60			180.60			180.60			180.60
		Glued Laminated Bamboo [kg/m² $_{RES}$]												
	Underground structure (UGS)	Concrete C25/30 [kg/m² $_{RES}$]	151.65	119.65	119.65	114.35	59.82	68.48	145.10	52.36	65.04	151.65	119.65	119.65
		Steel [kg/m² $_{RES}$]	15.53	12.25	12.25	11.71	6.13	7.01	50.01	5.36	6.66	15.53	12.25	12.25
	Windows	Insulated Triple Glazing [kg/m² $_{RES}$]	0.16	0.17	0.17	0.17	0.18	0.18	0.18	0.18	0.18	0.16	0.17	0.17
		PVC frame [kg/m² $_{RES}$]	1.42			1.51			1.61			1.42		
		Wood aluminum frame [kg/m² $_{RES}$]		1.28			1.34			1.36			1.28	
		Wood frame [kg/m² $_{RES}$]			1.28			1.34			1.36			1.28
	Waterproofing membrane (WM)	Polyethylene [kg/m² $_{RES}$]	1.50	1.50	1.50	0.75	0.75	0.75	0.60	0.60	0.60	1.50	1.50	1.50
	Finishing (Fi)	Solid wood (hardwood) [kg/m² $_{RES}$]		14.47			8.71			8.32			9.22	
		Solid wood (softwood) [kg/m² $_{RES}$]		9.00			9.00			9.00			9.00	
		Ceramic tiles [kg/m³ $_{RES}$]	18.000			18.000			18.000			18.000		
		Mineral plaster [kg/m² $_{RES}$]	71.72			22.00			22.00			22.00		
		Gypsum plasterboard [kg/m² $_{RES}$]		21.25			17.85			17.53			18.28	
		Bamboo Flooring [kg/m² $_{RES}$]			9.75			9.75			9.75			9.75
		Clay plaster [kg/m² $_{RES}$]			36.00			36.00			36.00			36.00
		Bamboo Cladding [kg/m² $_{RES}$]			14.69			8.84			8.45			9.36
	Biobased Insulation (BI)	Reed mats (max)	706.819	230.437	141.92	579.698	156.707	87.52	785.170	148.779	84.16	591.667	212.107	133.24
		Hemp fibers (min)	742.709	230.437	149.13	609.133	164.664	91.96	825.038	156.333	88.44	621.710	222.876	140.00
		Straw (med)	547.440	169.852	109.92	448.983	121.372	67.78	608.124	115.231	65.19	458.254	164.279	103.20
	Geometrical parameters (GP)	RES [m²] (reference for normalization)		145.00			842.00			1702.00			136.70	
		Number of conditioned storey		2.00			4.00			5.00			2.00	
		Roof area = basement= area single floor [m²/ m² $_{RES}$]		0.50			0.25			0.20			0.50	
		Exterior Wall area [m²/ m² $_{RES}$]		1.13			0.68			0.65			0.72	
		Window area [m²/ m² $_{RES}$]	0.15	0.16	0.15	0.16	0.17	0.16	0.17	0.17	0.17	0.16	0.16	0.16

Table S 8 – Final geometrical building configurations resumé showing the geometrical values and the material quantity used

3. Further analysis: Contribution to global warming with a time horizon of 20 years (GWP20)

The contribution to global warming with a time horizon of 20 years (GWP_{20}) can be calculated by keeping the same formulation explained in paragraph 1.5, with a replacement of the GWP_{100} index for each BT and material diets with the GWP_{20} one, still based on IPCC 2013 assessment method. In this case the building element replacement happens beyond the 20 years' time horizon chose, therefore was not considered.
The indexes were assessed via SimaPro 8 by using processes from Ecoinvent 3 [10]. The same processes from Ecoinvent shown in Table S2 were assumed for the calculation with time horizon of 20 years. Moreover, the $GWP_{bio\ index,dyn\ 0\text{-}20y}$ index were computed accordingly within the same time frames through a Dynamic Life cycle assessment with the "DynCO2" calculation tool [19], to provide new indexes for three different rotation growth: 90 years, 5 years, and 1 year. The achieved values are respectively: -0.12, -0.92, and -1.00. Therefore, the higher - in absolute value - the index is and the more effective the CO_2 stored in the vegetal mass is in CO_2 removal. Consequently, if on one side the fossil based $GWP_{20,IPCC}$ values per functional unit (FU) are larger than the ones calculated at 100 years, on the other side the carbon removal potential for shorter time horizon results in larger absolute values as well, due to the longer integration period over which the remaining fossil CO_2 in the atmosphere is responsible for a greater influence on radiative forcing. As a matter of fact, from Figure 4 in the main paper, it is possible to appreciate that we are already climate-negative by just using fast-growing biomass as structural materials.
In Table S 9, the new resulting Net-GWP for the 20 years' time horizon are reported.

Materials	λ	T	ρ_0	CC	BC	CO_2 Storage	GWP 20, IPCC	GWP bio index, dyn 0-20y	GWP_{bio}	Net-GWP value	Ref
Family	[W/mK]	[m]	[kg/m³]	[%]	[%]	$kgCO_2$/kg	[kg CO_{2eq}/kg]	[kg CO_{2eq}/$kgCO_2$]	[kg CO_{2eq}/kg]	[kg CO_{2eq}/kg]	9,10
Insulated Triple Glazing	/	0.040	30	0%	0%	0.0	57.00	0.00	0.00	57.00	9,10
Wood-Aluminium Window Frame	/	0.080	1042.5	0%	0%	0.0	5.60	0.00	0.00	5.60	9,10
PVC Window Frame	/	0.080	1181.25	0%	0%	0.0	3.27	0.00	0.00	3.27	9,10
Waterproof membrane Polyethylene	/	0.0015	1000	0%	0%	0.0	3.62	0.00	0.00	3.62	9,10
Wood Window Frame	/	0.080	1002.5	0%	0%	0.0	2.72	0.00	0.00	2.72	9,10
Steel (reinforcement)	/	/	7850	0%	0%	0.0	2.48	0.00	0.00	2.48	9,10
Mineral plaster	0.080	0.020	1100	0%	0%	0.0	0.61	0.00	0.00	0.61	9,10
Ceramic tiles	/	0.009	2000	0%	0%	0.0	0.55	0.00	0.00	0.55	9,10
Cross Laminated Timber (CLT)	/	/	427	50%	98%	-1.8	0.56	-0.12	-0.22	0.33	9,10, 20, 21
Glulam (GLT)	/	/	427	50%	98%	-1.8	0.56	-0.12	-0.22	0.33	9,10, 20, 21
OSB	/	/	550	50%	98%	-1.8	0.58	-0.12	-0.22	0.35	9,10
Gypsum plasterboard	0.210	0.0125	850	0%	0%	0.0	0.22	0.00	0.00	0.22	9,10
Concrete C30/37	/	/	2300	0%	0%	0.0	0.16	0.00	0.00	0.16	9,10
Concrete C25/30	/	/	2300	0%	0%	0.0	0.14	0.00	0.00	0.14	9,10
Concrete deep foundations	/	/	2325	0%	0%	0.0	0.12	0.00	0.00	0.12	9,10
Solid wood (softwood)	/	0.020	450	50%	100%	-1.8	0.27	-0.12	-0.23	0.04	9,10, 20
Clay plaster	0.470	0.020	1800	0%	0%	0.0	0.03	0.00	0.00	0.03	9,10
Bamboo Cladding	0.340	0.0200	650	54%	97%	-1.9	1.05	-0.92	-1.76	-0.71	22, 23, 10
Bamboo Flooring	/	0.015	650	54%	97%	-1.9	1.05	-0.92	-1.76	-0.71	9,10, 20, 21
Solid wood (hardwood)	0.120	0.020	640	50%	100%	-1.8	0.05	-0.12	-0.23	-0.18	9,10, 20, 21
Glue Laminated Bamboo (GLB)	/	/	636	54%	97%	-1.9	0.86	-0.92	-1.76	-0.90	22, 23, 10

**Cross Laminated Bamboo (CLB)**	/	/	636	54%	98%	-1.9	0.86	-0.92	-1.78	-0.92	22, 23, 10
**Hemp fibers**	0.049	/	86	45%	65%	-1.1	0.07	-1.00	-1.07	-1.00	7, 24, 10
**Reed mats**	0.0505	/	180.5	47%	98%	-1.7	0.39	-1.00	-1.69	-1.30	7, 25, 10
**Straw**	0.0525	/	95	40%	100%	-1.5	0.11	-1.00	-1.47	-1.36	7, 24, 10

Table S 9 – Properties of construction materials used with a time horizon of 20 years. In particular: Thermal conductivity (λ), thickness, dry density (ρ_0), Carbon Content (CC), Biomass Content (BC), CO_2 Storage ($CO_{2\ Storage}$), Global Worming Potential at 20 years ($GWP_{20,IPCC}$), Global Worming Potential bio indexes calculated with the dynamic tool for the replaced elements after 20 years ($GWP_{bio\ index,\ dyn\ 0\text{-}20y}$), Global Worming Potential considering the biogenic portion (GWP_{Bio}), Net-GWP value expressed per kg or m^3. Materials highlighted in red are High-Carbon, in yellow are Low-Carbon and in green Climate-negative.

4. References

(1) Intelligent Energy Europe. European Projects TABULA & EPISCOPE **2016,** https://episcope.eu/welcome/ (accessed May 12, 2020).

(2) Khan, F. R. Evolution of Structual Systems for High Rise Buildings in Steel and Concrete. In *Regional Conference on Tall Buildings*; Bratislava, Czechoslocakia, **1973**; pp 13–14.

(3) MathWorks. MATLAB. **2012**, Inc., Natick, Massachusetts, United States

(4) Platt, S.; Maskell, D.; Walker, P.; Laborel-Préneron, A. Manufacture and Characterisation of Prototype Straw Bale Insulation Products. *Construction and Building Materials* **2020**, *262*, 120035. https://doi.org/10.1016/j.conbuildmat.2020.120035.

(5) Marques, B.; Tadeu, A.; Almeida, J.; António, J.; de Brito, J. Characterisation of Sustainable Building Walls Made from Rice Straw Bales. *Journal of Building Engineering* **2020**, *28* (October 2019). https://doi.org/10.1016/j.jobe.2019.101041.

(6) Ashour, T.; Georg, H.; Wu, W. Performance of Straw Bale Wall: A Case of Study. *Energy and Buildings* **2011**, *43* (8), 1960–1967. https://doi.org/10.1016/j.enbuild.2011.04.001.

(7) Schiavoni, S.; D'Alessandro, F.; Bianchi, F.; Asdrubali, F. Insulation Materials for the Building Sector: A Review and Comparative Analysis. *Renewable and Sustainable Energy Reviews* **2016**, *62*, 988–1011. https://doi.org/10.1016/j.rser.2016.05.045.

(8) Masera, O. R.; Garza-Caligaris, J. F.; Kanninen, M.; Karjalainen, T.; Liski, J.; Nabuurs, G. J.; Pussinen, A.; De Jong, B. H. J.; Mohren, G. M. J. Modeling Carbon Sequestration in Afforestation, Agroforestry and Forest Management Projects: The CO2FIX V.2 Approach. *Ecological Modelling* **2003**, *164* (2–3), 177–199. https://doi.org/10.1016/S0304-3800(02)00419-2.

(9) Koordinationskonferenz der Bau-und Liegenschaftsorgane der Öffentlichen Bauherren KBOB **2012**. Ökobilanzdaten im Baubereich 2009/1. Bern. KBOB

(3) Haefliger, I.-F.; John, V.; Lasvaux, V.; Hoxha,E., Passer, A. ; Habert, G. Towards a reliable comparison between environmental and economic cost of Swiss dwellings: A model with building materials' service life uncertainty. **2016**. Available from: https://www.researchgate.net/publication/304038702_Towards_a_reliable_comparison_between_environmental_and_economic_cost_of_Swiss_dwellings_A_model_with_building_materials'_service_life_uncertainty [accessed Feb 11 2022].

(10) Wernet, G.; Bauer, C.; Steubing, B.; Reinhard, J.; Moreno-Ruiz, E.; Weidema, B. The Ecoinvent Database Version 3 (Part I): Overview and Methodology. The International Journal of Life Cycle Assessment. **2016**.

(11) Campiglia, E.; Gobbi, L.; Marucci, A.; Rapa, M.; Ruggieri, R.; Vinci, G. Hemp Seed Production: Environmental Impacts of Cannabis Sativa L. Agronomic Practices by Life Cycle Assessment (LCA) and Carbon Footprint Methodologies. *Sustainability (Switzerland)* **2020**, *12* (16). https://doi.org/10.3390/su12166570.

(12) Zampori, L.; Dotelli, G.; Vernelli, V. Life Cycle Assessment of Hemp Cultivation and Use of Hemp-Based Thermal Insulator Materials in Buildings. *Environmental Science and Technology* **2013**. https://doi.org/10.1021/es401326a.

(13) De Wolf, C.; Pomponi, F.; Moncaster, A. Measuring Embodied Carbon Dioxide Equivalent of Buildings: A Review and Critique of Current Industry Practice. *Energy and Buildings* **2017**, *140*, 68–80. https://doi.org/10.1016/j.enbuild.2017.01.075.

(14) Sébastien, L.; Goulouti, K.; Favre, D.; Giorgi, M.; Padey, P.; Volland, B.; Farsi, M.; Habert,

G.; Galimshina, A. Analysis of Lifetimes of Building Elements in the Literature and in Renovation Practices and Sensitivity Analyses on Building LCA & LCC. *Erneuern! Sanierungsstrategien für den Gebäudepark* **2020**, No. October.
(15) Heeren, N.; Mutel, C. L.; Steubing, B.; Ostermeyer, Y.; Wallbaum, H.; Hellweg, S. Environmental Impact of Buildings - What Matters? *Environmental Science and Technology* **2015**, *49* (16), 9832–9841. https://doi.org/10.1021/acs.est.5b01735.
(16) Göswein, V.; Silvestre, J. D.; Sousa Monteiro, C.; Habert, G.; Freire, F.; Pittau, F. Influence of Material Choice, Renovation Rate, and Electricity Grid to Achieve a Paris Agreement-Compatible Building Stock: A Portuguese Case Study. *Building and Environment* **2021**, *195* (March). https://doi.org/10.1016/j.buildenv.2021.107773.
(17) Levasseur, A.; Lesange, P.; Margini, M.; Deschenes, L.; Samson, R. Considering Time in LCA: Dynamic LCA and Its Application to Global Warming Impact Assessments. *Environmetal, Science & Technology* **2010**, *44*. https://doi.org/10.1021/es9030003.
(18) Guest, G.; Cherubini, F.; Strømman, A. H. Global Warming Potential of Carbon Dioxide Emissions from Biomass Stored in the Anthroposphere and Used for Bioenergy at End of Life. *Journal of Industrial Ecology* **2013**, *17* (1), 20–30. https://doi.org/10.1111/j.1530-9290.2012.00507.x.
(19) Levasseur, A. New Generation Carbon Footprinting, CIRAIG **2010**, https://ciraig.org/index.php/project/dynco2-dynamic-carbon-footprinter/ (accessed Jul 15, 2021).
(20) Thomas, S. C.; Martin, A. R. Carbon Content of Tree Tissues: A Synthesis. *Forests* **2012**, *3* (2), 332–352. https://doi.org/10.3390/f3020332.
(21) Yusof, N. M.; Tahir, P. M.; Lee, H.; Khan, M. A.; Mohammad, R.; James, S. Mechanical and Physical Properties of Cross-Laminated Timber Made from Acacia Mangium Wood as Function of Adhesive Types. https://doi.org/10.1186/s10086-019-1799-z.
(22) Lugt, P. Van Der; Vogtlander, J. *K _\ < Em ` Ifed \ EkXc @ DgXZk f ] @ e [ Ljki ` Xc 9XdYff Gif [LZkj*; 2015. https://doi.org/10.13140/RG.2.2.20797.46560.
(23) Pongon, R. S.; Aranico, E. C.; Dagoc, F. L. S.; Jr, R. F. A. Carbon Stock Assessment of Bamboo Plantations In Northern Mindanao, Philippines. *Journal of Biodiversity and Environmental Sciences* **2016**, *9* (6), 97–112.
(24) Pittau, F.; Krause, F.; Lumia, G.; Habert, G. Fast-Growing Bio-Based Materials as an Opportunity for Storing Carbon in Exterior Walls. *Building and Environment* **2018**, *129* (August 2017), 117–129. https://doi.org/10.1016/j.buildenv.2017.12.006.
(25) Bumane, S.; Poisa, L.; Cubars, E.; Platace, R. The Analysis of Carbon Content in Different Energy Crops. *Nordic View to Sustainable Rural Development* **2015**, 156–160.
(26) Hoxha, E.; Passer, A.; Saade, M. R. M.; Trigaux, D.; Shuttleworth, A.; Pittau, F.; Allacker, K.; Habert, G. Biogenic Carbon in Buildings: A Critical Overview of LCA Methods. *Buildings and Cities* **2020**, *1* (1), 504–524. https://doi.org/10.5334/bc.46.
(27) ISO/TS 14067. *Greenhouse Gases — Carbon Footprint of Products — Requirements and Guidelines for Quantification*; **2018**.
(28) Levasseur, A.; Lesage, P.; Margni, M.; Brandão, M.; Samson, R. Assessing Temporary Carbon Sequestration and Storage Projects through Land Use, Land-Use Change and Forestry: Comparison of Dynamic Life Cycle Assessment with Ton-Year Approaches. *Climatic Change* **2012**, *115* (3–4), 759–776. https://doi.org/10.1007/s10584-012-0473-x.
(29) PAS 2050:2011. Specification for the Assessment of the Life Cycle Greenhouse Gas Emissions of Goods and Services. **2011**, 45.
(30) Wolf, M.-A.; Pant, R.; Chomkhamsri, K.; Sala, S.; Pennington, D. *The International Reference Life Cycle Data System (ILCD) Handbook*; 2012. https://doi.org/10.2788/85727.
(31) Peñaloza, D.; Erlandsson, M.; Falk, A. Exploring the Climate Impact Effects of Increased Use of Bio-Based Materials in Buildings. *Construction and Building Materials* **2016**, *125*, 219–226. https://doi.org/10.1016/J.CONBUILDMAT.2016.08.041.
(32) Pittau, F.; Krause, F.; Lumia, G.; Habert, G. Fast-Growing Bio-Based Materials as an Opportunity for Storing Carbon in Exterior Walls. *Building and Environment* **2018**, *129* (August 2017), 117–129. https://doi.org/10.1016/j.buildenv.2017.12.006.
(33) Cherubini, F.; Peters, G. P.; Berntsen, T.; Strømman, A. H.; Hertwich, E. CO2 Emissions from Biomass Combustion for Bioenergy: Atmospheric Decay and Contribution to Global

Warming. *GCB Bioenergy* **2011**, *3* (5), 413–426. https://doi.org/10.1111/j.1757-1707.2011.01102.x.
(34) Guest, G.; Cherubini, F.; Strømman, A. H. Global Warming Potential of Carbon Dioxide Emissions from Biomass Stored in the Anthroposphere and Used for Bioenergy at End of Life. *Journal of Industrial Ecology* **2013**, *17* (1), 20–30. https://doi.org/10.1111/j.1530-9290.2012.00507.x.
(35) Hoxha, E.; Passer, A.; Saade, M. R. M.; Trigaux, D.; Shuttleworth, A.; Pittau, F.; Allacker, K.; Habert, G. Biogenic Carbon in Buildings: A Critical Overview of LCA Methods. *Buildings and Cities* **2020**, *1* (1), 504–524. https://doi.org/10.5334/bc.46.
(36) Levasseur, A.; Lesage, P.; Margni, M.; Samson, R. Biogenic Carbon and Temporary Storage Addressed with Dynamic Life Cycle Assessment. *Journal of Industrial Ecology* **2013**, *17* (1), 117–128. https://doi.org/10.1111/j.1530-9290.2012.00503.x.
(37) Vogtländer, J. G.; van der Lugt, P. *The Environmental Impact of Industrial Bamboo Products: Life Cycle Assessment and Carbon Sequestration*; **2015**. https://doi.org/10.13140/RG.2.2.20797.46560.
(38) Le Guen, A.; Ravasse, F. CEN/TC 124. **2012**, *CEN/TC 124* (1130), 0–7.

5. Annex A

MATLAB Script

```
% Description:      This Script analyses the required materials volumes in a given structure to withstand vertical loads.
%                   It is subdivided in 4 sub-scripts, one for each different structural scheme and different material.
%                   Horizontal and vertical elements are proven, as well as the foundation of the generated building.
%                   The code iterative generates a building with a given number of stories
%                   and proofs at each step the statically required dimensions of the structural elements.
%                   By adding these together at each iteration step, plots are generated showing the materials volumes
%                   required at each story height and for each different structural material scheme.
%% ------------- BEGIN CODE --------------
%% SCHEME 1 : In-situ cast concrete columns and wall supporting a reinforcing concrete plate
maxstories = 14;
volumeslab_concrete_tot = zeros(maxstories,1);
volumeslab_armature_tot = zeros(maxstories,1);
k_c_found = zeros(maxstories,1);
A_co = zeros(maxstories,1);
V_co = zeros(maxstories,1);
volumecolumns_concrete = zeros(maxstories,1);
volumewalls_concrete = zeros(maxstories,1);
volumecolumns_armature = zeros(maxstories,1);
volumewalls_armature = zeros(maxstories,1);
volumevertical_concrete = zeros(maxstories,1);
volumevertical_armature = zeros(maxstories,1);
volumecolumns_concrete_tot = zeros(maxstories,1);
volumewalls_concrete_tot = zeros(maxstories,1);
volumecolumns_armature_tot = zeros(maxstories,1);
volumewalls_armature_tot = zeros(maxstories,1);
volumevertical_concrete_tot = zeros(maxstories,1);
volumevertical_armature_tot = zeros(maxstories,1);
volumefoundation_concrete = zeros(maxstories,1);
volumefoundation_armature = zeros(maxstories,1);
nr_micropiles_tot = zeros(maxstories,1);
volumemicropiles = zeros(maxstories,1);
volume_armature_overground_tot = zeros(maxstories,1);
usageratio_overground_C2530 = zeros(maxstories,1);
usageratio_overground_C3037 = zeros(maxstories,1);
usageratio_foundation = zeros(maxstories,1);
usageratio_armature_overground_tot = zeros(maxstories,1);
usageratio_armature_underground_tot = zeros(maxstories,1);
usageratio_micropiles = zeros(maxstories,1);
usageratio_concrete_tot = zeros(maxstories,1);
usageratio_impact_concrete = zeros(maxstories,1);
usageratio_impact_steel = zeros(maxstories,1);
usageratio_impact_tot = zeros(maxstories,1);
for n_st_o=1:maxstories
%% Building Geometry
H_st=2.7;%Intern Height of story
L=10;
B=10;
H=n_st_o*(H_st+0.5);%30cm of suspended ceiling and 40cm/2 of Floor Beams height
```

```
s1=5;% s1 => s2 , as the static verifications are apported on the length of s1
s2=5;%spacing of intern walls
usage_resid=0;
usage_office=1;

%Display Variables
geometryvar_names = {'n_st_o' 'stories';
    'H_st' 'm';
    'L' 'm';
    'B' 'm';
    'H' 'm';
    's1' 'm';
    's2' 'm';
    'usage_resid' '(1=100%)';
    'usage_office' '(1=100%)'};
geometryvar_values = [n_st_o,H_st,L,B,H,s1,s2,...
    usage_resid,usage_office];
disp(char(' '))
disp(char(' '))
disp(char(' '))
disp(char({'Building Geometry';''}))

for i = 1:size(geometryvar_names,1)
    disp([char(geometryvar_names(i,1)),' = ', num2str(geometryvar_values(i)),'  ',
char(geometryvar_names(i,2))])
end
%% Slab
%Material Properties
sortofmaterialslab='concrete';
strengthclassslab='C25/30';
if strcmp(strengthclassslab,'C25/30')
    f_c_d=16.5;%connect this value to the material with if function.
else%C30/37
    f_c_d=20;
end
f_s_d=435;

%Bending Resistence verification
%Profile
h_sl=0.01;%ev. minimal thickness of ceiling
P_sl=0.01;%armature content for 4 layers of ?10@150 and 3 strongband over rows of columns of
1mx?16@150mm
p_sl_o=P_sl*1/4;%upper layer around 1/4 of total armature (4 layers and 3 strongbands)
p_sl_u=P_sl*1/5;%lower layer equal upper -> plastification possible
a_s_o=h_sl*p_sl_o*10^6;%in mm^2/m'
a_s_u=h_sl*p_sl_u*10^6;%in mm^2/m'

%Loads
g_0k_sl=25;
g_Ak_sl=1;%Assumption of 100kg/m^2
q_Nk_S=2.5;
q_Nk_resid=2;
q_Nk_office=3;
q_k_sl=h_sl*g_0k_sl+g_Ak_sl+max(q_Nk_S,q_Nk_resid*usage_resid+q_Nk_office*usage_office);
%kN/m'
```

```
q_d_sl=1.35*(h_sl*g_0k_sl+g_Ak_sl)+1.5*max(q_Nk_S,q_Nk_resid*usage_resid+q_Nk_office*usage_office);%kN/m'
%Strongbands (sb)
%a_sb=1;%1m width strongband
%q_k_sl_sb=q_k_sl/2*s1/(2*a_sb);
%q_d_sl_sb=q_d_sl/2*s1/(2*a_sb);
%Total
%q_k_sl_tot=q_k_sl+q_k_sl_sb;
%q_d_sl_tot=q_d_sl+q_d_sl_sb;
m_d_sl_o=q_d_sl*s1^2/12;%upper layer
m_d_sl_u=q_d_sl*s1^2/24;%lower layer

%Bending resistence (SIA 265,(14))
m_rd_sl_o=a_s_o*f_s_d*(0.9*h_sl*1000-a_s_o*f_s_d/(2*f_c_d*1000))/10^6;%in kNm/m'
m_rd_sl_u=a_s_u*f_s_d*(0.9*h_sl*1000-a_s_u*f_s_d/(2*f_c_d*1000))/10^6;%in kNm/m'

%Display Variables
%slabvar_names = {'f_c_d' 'N/mm^2';'f_s_d' 'N/mm^2';'h_sl' 'm';'P_sl' '-';'p_sl_o' '-';'p_sl_u' '-';'a_s_o' 'mm^2/m';'a_s_u' 'mm^2/m';'g_0k_sl' 'kN/m^3';'g_Ak_sl' 'kN/m^2';'q_Nk_S' 'kN/mm^2';'q_Nk_resid' 'kN/m^2';'q_Nk_office' 'kN/m^2';'q_k_sl' 'kN/m^2';'q_d_sl' 'kN/m^2';'a_sb' 'm';'q_k_sl_sb' 'kN/m^2';'q_d_sl_sb' 'kN/m^2';'q_k_sl_tot' 'kN/m^2';'q_d_sl_tot' 'kN/m^2';'m_d_sl_o' 'kNm/m   (must be < m_rd_sl_o)';'m_d_sl_u' 'kNm/m   (must be < m_rd_sl_u)';'m_rd_sl_o' 'kNm/m';'m_rd_sl_u' 'kNm/m'};
%slabvar_values =
[f_c_d,f_s_d,h_sl,P_sl,p_sl_o,p_sl_u,a_s_o,a_s_u,g_0k_sl,g_Ak_sl,q_Nk_S,q_Nk_resid,q_Nk_office,q_k_sl,q_d_sl,a_sb,q_k_sl_sb,q_d_sl_sb,q_k_sl_tot,q_d_sl_tot,m_d_sl_o,m_d_sl_u,m_rd_sl_o,m_rd_sl_u];

%disp(char(' '))
%disp(char(' '))
%disp('Slab verification')
%disp(['Material:',' ',char(sortofmaterialslab)])
%disp(['Strength Class:',' ',char(strengthclassslab)])
%disp(char({'';'';'Start Bending Values Slabs';''}))

%for i = 1:size(slabvar_names,1)
   %disp([char(slabvar_names(i,1)),' = ', num2str(slabvar_values(i)),'  ', char(slabvar_names(i,2))])
%end
%disp(char(' '))
%disp([char('h_sl'),' = ', num2str(h_sl),'  ', char('m')])
%disp([char('Armature content'),' = ', num2str(P_sl),'  ', char('-')])
%disp(char(' '))

while (m_d_sl_o > m_rd_sl_o) || (m_d_sl_u > m_rd_sl_u)
   %Bending Resistence verification
   %Profile
   h_sl=h_sl + 0.01;%ev. minimal thickness of ceiling
   a_s_o=h_sl*p_sl_o*10^6;%in mm^2/m'
   a_s_u=h_sl*p_sl_u*10^6;%in mm^2/m'

   %Loads
   g_0k_sl=25;
   g_Ak_sl=1;%Assumption of 100kg/m^2
   q_Nk_S=2.5;
   q_Nk_resid=2;
   q_Nk_office=3;
```

```
q_k_sl=h_sl*g_0k_sl+g_Ak_sl+max(q_Nk_S,q_Nk_resid*usage_resid+q_Nk_office*usage_office);
%kN/m'

q_d_sl=1.35*(h_sl*g_0k_sl+g_Ak_sl)+1.5*max(q_Nk_S,q_Nk_resid*usage_resid+q_Nk_office*usa
ge_office);%kN/m'
    %Strongbands (sb)
    %a_sb=1;%1m width strongband
    %q_k_sl_sb=q_k_sl/2*s1/(2*a_sb);
    %q_d_sl_sb=q_d_sl/2*s1/(2*a_sb);
    %Total
    %q_k_sl_tot=q_k_sl+q_k_sl_sb;
    %q_d_sl_tot=q_d_sl+q_d_sl_sb;
    m_d_sl_o=q_d_sl*s1^2/12;%upper layer
    m_d_sl_u=q_d_sl*s1^2/24;%lower layer

    %Bending resistence (SIA 265,(14))
    m_rd_sl_o=a_s_o*f_s_d*(0.9*h_sl*1000-a_s_o*f_s_d/(2*f_c_d*1000))/10^6;%in kNm/m'
    m_rd_sl_u=a_s_u*f_s_d*(0.9*h_sl*1000-a_s_u*f_s_d/(2*f_c_d*1000))/10^6;%in kNm/m'
end

%Display Variables
%slabvar_names = {'f_c_d' 'N/mm^2';'f_s_d' 'N/mm^2';'h_sl' 'm';'P_sl' '-';'p_sl_o' '-';'p_sl_u' '-
';'a_s_o' 'mm^2/m';'a_s_u' 'mm^2/m';'g_0k_sl' 'kN/m^3';'g_Ak_sl' 'kN/m^2';'q_Nk_S'
'kN/mm^2';'q_Nk_resid' 'kN/m^2';'q_Nk_office' 'kN/m^2';'q_k_sl' 'kN/m^2';'q_d_sl' 'kN/m^2';'a_sb'
'm';'q_k_sl_sb' 'kN/m^2';'q_d_sl_sb' 'kN/m^2';'q_k_sl_tot' 'kN/m^2';'q_d_sl_tot' 'kN/m^2';'m_d_sl_o'
'kNm/m   (must be < m_rd_sl_o)';'m_d_sl_u' 'kNm/m   (must be < m_rd_sl_u)';'m_rd_sl_o'
'kNm/m';'m_rd_sl_u' 'kNm/m'};
%slabvar_values =
[f_c_d,f_s_d,h_sl,P_sl,p_sl_o,p_sl_u,a_s_o,a_s_u,g_0k_sl,g_Ak_sl,q_Nk_S,q_Nk_resid,q_Nk_offi
ce,q_k_sl,q_d_sl,a_sb,q_k_sl_sb,q_d_sl_sb,q_k_sl_tot,q_d_sl_tot,m_d_sl_o,m_d_sl_u,m_rd_sl_o,
m_rd_sl_u];

%disp(char({'';'';'Final Bending Values Slabs';''}))

%for i = 1:size(slabvar_names,1)
    %disp([char(slabvar_names(i,1)),' = ', num2str(slabvar_values(i)),'  ', char(slabvar_names(i,2))])
%end
%disp(char(' '))
%disp([char('h_sl'),' = ', num2str(h_sl),'  ', char('m')])
%disp([char('Armature content'),' = ', num2str(P_sl),'  ', char('-')])
%disp(char(' '))


%Deflection verification
%h_sl taken from bending resistence verification,ev. further reduced if deflection is determinating
%Material Properties
if strcmp(strengthclassslab,'C25/30')
    E_c_m=32000;%connect this value to the material with if function.
    f_ctm=2.6;
else%C30/37
    E_c_m=33600;
    f_ctm=2.9;
end

%Profile
```

```
W_sl=h_sl^2/6*1;%in m^3/m
k_t=1/(1+0.5*h_sl/3);
f_ctd=k_t*f_ctm;
m_r_crack_sl=W_sl*f_ctd*1000;%in kNm/m'

I_sl=h_sl^3/12*1;%in m^4/m
phi_creep=2;%assumed
if m_r_crack_sl > m_d_sl_u
   f_creep=1+phi_creep;%uncracked concrete
else
   f_creep=(1-20*p_sl_u)/(10*p_sl_o^0.7)*(0.75+0.1*phi_creep)*(1/0.9)^3;%cracked concrete
end

%Loads (SIA 260,A,tab.2)
psi_1=0.5;%frequent
psi_2=0.3;%quasi-permanent
%rare
q_k_sl_rare=h_sl*g_0k_sl+g_Ak_sl+...
   max(q_Nk_S,q_Nk_resid*usage_resid+q_Nk_office*usage_office);%only one payload acting
%frequent
q_k_sl_fr=h_sl*g_0k_sl+g_Ak_sl+...
   psi_1*max(q_Nk_S,q_Nk_resid*usage_resid+q_Nk_office*usage_office);%only one payload
acting
%quasi-permanent
q_k_sl_qp=h_sl*g_0k_sl+g_Ak_sl+...
   psi_2*max(q_Nk_S,q_Nk_resid*usage_resid+q_Nk_office*usage_office);%only one payload
acting

%Deflections (SIA 260,A,tab.3)
%rare
w_sl_rare=5/384*q_k_sl_rare*s1^4/(E_c_m*I_sl)*f_creep*1.5;%2-field carrier and 2D deflection
w_sl_lim_rare=s1/0.500;
%frequent
w_sl_fr=5/384*q_k_sl_fr*s1^4/(E_c_m*I_sl)*f_creep*1.5;%2-field carrier and 2D deflection
w_sl_lim_fr=s1/0.350;
%quasi-permanent
w_sl_qp=5/384*q_k_sl_qp*s1^4/(E_c_m*I_sl)*f_creep*1.5;%2-field carrier and 2D deflection
w_sl_lim_qp=s1/0.300;

%Display Variables
%slabvar_names2 = {'E_c_m' 'kN/mm^2';'f_ctm' 'N/mm^2';'h_sl' 'm';'W_sl' 'm^3/m`';'k_t' '-';'f_ctd'
'N/mm^2';'m_r_crack_sl' 'kNm/m`';'I_sl' 'm^4/m`';'phi_creep' '-';'f_creep' '-   (1+phi for uncracked
concrete)';'psi_1' '-';'psi_2' '-';'q_k_sl_rare' 'kN/m^2';'q_k_sl_fr' 'kN/m^2';'q_k_sl_qp'
'kN/m^2';'w_sl_rare' 'mm   (must be < w_sl_lim_rare)';'w_sl_lim_rare' 'mm';'w_sl_fr' 'mm   (must be
< w_sl_lim_fr)';'w_sl_lim_fr' 'mm';'w_sl_qp' 'mm   (must be < w_sl_lim_qp)';'w_sl_lim_qp' 'mm'};
%slabvar_values2 =
[E_c_m,f_ctm,h_sl,W_sl,k_t,f_ctd,m_r_crack_sl,I_sl,phi_creep,f_creep,psi_1,psi_2,q_k_sl_rare,q_
k_sl_fr,q_k_sl_qp,w_sl_rare,w_sl_lim_rare,w_sl_fr,w_sl_lim_fr,w_sl_qp,w_sl_lim_qp];

%disp(char({'';'';'Start Deflection Values Slabs';''}))

%for i = 1:size(slabvar_names2,1)
   %disp([char(slabvar_names2(i,1)),' = ', num2str(slabvar_values2(i)),'  ',
char(slabvar_names2(i,2))])
%end
%disp(char(' '))
```

```
%disp([char('h_sl'),' = ', num2str(h_sl),'  ', char('m')])
%disp([char('Armature content'),' = ', num2str(P_sl),'  ', char('-')])
%disp(char(' '))


while (w_sl_rare > w_sl_lim_rare)||(w_sl_fr > w_sl_lim_fr)||(w_sl_qp > w_sl_lim_qp)
   %Profile
   h_sl = h_sl + 0.01;
   W_sl=h_sl^2/6*1;%in m^3/m
   k_t=1/(1+0.5*h_sl/3);
   f_ctd=k_t*f_ctm;
   m_r_crack_sl=W_sl*f_ctd*1000;%in kNm/m'

   I_sl=h_sl^3/12*1;%in m^4/m
   phi_creep=2;%assumed
   if m_r_crack_sl > m_d_sl_u
      f_creep=1+phi_creep;%uncracked concrete
   else
      f_creep=(1-20*p_sl_u)/(10*p_sl_o^0.7)*(0.75+0.1*phi_creep)*(1/0.9)^3;%cracked concrete
   end

   %Loads (SIA 260,A,tab.2)
   psi_1=0.5;%frequent
   psi_2=0.3;%quasi-permanent
   %rare
   q_k_sl_rare=h_sl*g_0k_sl+g_Ak_sl+...
      max(q_Nk_S,q_Nk_resid*usage_resid+q_Nk_office*usage_office);%only one payload acting
   %frequent
   q_k_sl_fr=h_sl*g_0k_sl+g_Ak_sl+...
      psi_1*max(q_Nk_S,q_Nk_resid*usage_resid+q_Nk_office*usage_office);%only one payload
acting
   %quasi-permanent
   q_k_sl_qp=h_sl*g_0k_sl+g_Ak_sl+...
      psi_2*max(q_Nk_S,q_Nk_resid*usage_resid+q_Nk_office*usage_office);%only one payload
acting

   %Deflections (SIA 260,A,tab.3)
   %rare
   w_sl_rare=5/384*q_k_sl_rare*s1^4/(E_c_m*I_sl)*f_creep*1.5;%2-field carrier and 2D deflection
   w_sl_lim_rare=s1/0.500;
   %frequent
   w_sl_fr=5/384*q_k_sl_fr*s1^4/(E_c_m*I_sl)*f_creep*1.5;%2-field carrier and 2D deflection
   w_sl_lim_fr=s1/0.350;
   %quasi-permanent
   w_sl_qp=5/384*q_k_sl_qp*s1^4/(E_c_m*I_sl)*f_creep*1.5;%2-field carrier and 2D deflection
   w_sl_lim_qp=s1/0.300;
end

%Display Variables
%slabvar_names2 = {'E_c_m' 'kN/mm^2';'f_ctm' 'N/mm^2';'h_sl' 'm';'W_sl' 'm^3/m`';'k_t' '-';'f_ctd'
'N/mm^2';'m_r_crack_sl' 'kNm/m`';'I_sl' 'm^4/m`';'phi_creep' '-';'f_creep' '-   (1+phi for uncracked
concrete)';'psi_1' '-';'psi_2' '-';'q_k_sl_rare' 'kN/m^2';'q_k_sl_fr' 'kN/m^2';'q_k_sl_qp'
'kN/m^2';'w_sl_rare' 'mm   (must be < w_sl_lim_rare)';'w_sl_lim_rare' 'mm';'w_sl_fr' 'mm   (must be
< w_sl_lim_fr)';'w_sl_lim_fr' 'mm';'w_sl_qp' 'mm   (must be < w_sl_lim_qp)';'w_sl_lim_qp' 'mm'};
```

```
%slabvar_values2 =
[E_c_m,f_ctm,h_sl,W_sl,k_t,f_ctd,m_r_crack_sl,I_sl,phi_creep,f_creep,psi_1,psi_2,q_k_sl_rare,q_
k_sl_fr,q_k_sl_qp,w_sl_rare,w_sl_lim_rare,w_sl_fr,w_sl_lim_fr,w_sl_qp,w_sl_lim_qp];


%disp(char({'';'';'Final Deflection Values Slabs';''}))

%for i = 1:size(slabvar_names2,1)
   %disp([char(slabvar_names2(i,1)),' = ', num2str(slabvar_values2(i)),'  ',
char(slabvar_names2(i,2))])
%end
%disp(char(' '))
%disp([char('h_sl'),' = ', num2str(h_sl),'  ', char('m')])
%disp([char('Armature content'),' = ', num2str(P_sl),'  ', char('-')])
%disp(char(' '))

%Volumes
%1 Story
volumeslab_concrete=h_sl*B*L;
volumeslab_armature=volumeslab_concrete*P_sl;
%n Stories
volumeslab_concrete_tot(n_st_o)=volumeslab_concrete*n_st_o;
volumeslab_armature_tot(n_st_o)=volumeslab_armature*n_st_o;


% %Display Volumes
% volumeslabvar_names = {'Volume slabs' 'm^3   each story';
%     'Volume armature' 'm^3   each story';
%     'Total Volume slabs' 'm^3   over whole building';
%     'Total Volume armature' 'm^3   over whole building'};
% volumeslabvar_values = [volumeslab_concrete,volumeslab_armature,...
%     volumeslab_concrete_tot(n_st_o),volumeslab_armature_tot(n_st_o)];
%
%disp(char({'';'Total Volumes Slabs';''}))

%for i = 1:size(volumeslabvar_names,1)
    %disp([char(volumeslabvar_names(i,1)),' = ', num2str(volumeslabvar_values(i)),'  ',
char(volumeslabvar_names(i,2))])
%end
%disp(char(' '))
%% Columns
%Material Properties
sortofmaterialcolumn='concrete';
strengthclasscolumn='C30/37';
if strcmp(strengthclasscolumn,'C30/37')
    f_c_d=20;%connect this value to the material with if function.
else%C25/30
    f_c_d=16.5;
end

%Profile
P_co=0.01;%armature content
h_co=0.01;%ev. minimal profile height
b_co=h_co;%quadratic profils
L_co=H_st+h_sl/2+0.3;%30cm of suspended ceiling
A_co(n_st_o)=b_co*h_co;
```

```
V_co(n_st_o)=A_co(n_st_o)*L_co;

%Loads
g_0k_co=25;%25kN/m^3
g_Ak_co=1;%Assumption 100kg/m^2 walls aruond columns
N_k_co=(n_st_o-1)*(s1*s2*(h_sl*g_0k_sl+g_Ak_sl)+...
   (s1+s2)*L_co*g_Ak_co)+sum(V_co(1:n_st_o))*g_0k_co+...%Assumptions for an intern column
with intern Walls over it.
   s1*s2*(q_Nk_S+(n_st_o-1)*(q_Nk_resid*usage_resid+q_Nk_office*usage_office));
N_d_co=1.35*((n_st_o-1)*(s1*s2*(h_sl*g_0k_sl+g_Ak_sl)+...
   (s1+s2)*L_co*g_Ak_co)+sum(V_co(1:n_st_o))*g_0k_co)+...%Assumptions for an intern column
with intern Walls over it.
   1.5*(s1*s2*(q_Nk_S+(n_st_o-1)*(q_Nk_resid*usage_resid+q_Nk_office*usage_office)));

%Compression
sigma_c_d=N_d_co/A_co(n_st_o)/1000;%brought to N/mm^2

%Display Variables
%columnsvar_names = {'f_c_d' 'N/mm^2';'h_co' 'm';'b_co' 'm';'L_co' 'm';'A_co' 'm^2';'g_0k_co'
'kN/m^3';'g_Ak_co' 'kN/m^2';'N_k_co' 'kN';'N_d_co' 'kN';'sigma_c_d' 'N/mm^2   (must be <
f_c_0_d)';'f_c_d' 'N/mm^2'};
%columnsvar_values =
[f_c_d,h_co,b_co,L_co,A_co(n_st_o),g_0k_co,g_Ak_co,N_k_co,N_d_co,sigma_c_d,f_c_d];

%disp(char(' '))
%disp(char(' '))
%disp('Columns verification')
%disp(['Material:',' ',char(sortofmaterialcolumn)])
%disp(['Strength Class:',' ',char(strengthclasscolumn)])
%disp(char({'';'';'Start Values Columns';''}))

%for i = 1:size(columnsvar_names,1)
   %disp([char(columnsvar_names(i,1)),' = ', num2str(columnsvar_values(i)),'  ',
char(columnsvar_names(i,2))])
%end
%disp(char(' '))
%disp([char('h_co'),' = ', num2str(h_co),'  ', char('m')])
%disp([char('b_co'),' = ', num2str(b_co),'  ', char('m')])
%disp([char('Armature content'),' = ', num2str(P_co),'  ', char('-')])
%disp(char(' '))

while sigma_c_d > f_c_d
   %Profile
   h_co=h_co + 0.05;
   b_co=h_co;%quadratic profils
   L_co=H_st+h_sl/2+0.3;%30cm of suspended ceiling
   A_co(n_st_o)=b_co*h_co;
   V_co(n_st_o)=A_co(n_st_o)*L_co;

   %Loads
   g_0k_co=25;%5kN/m^3
   g_Ak_co=1;%Assumption 100kg/m^2 walls aruond columns
   N_k_co=(n_st_o-1)*(s1*s2*(h_sl*g_0k_sl+g_Ak_sl)+...
      (s1+s2)*L_co*g_Ak_co)+sum(V_co(1:n_st_o))*g_0k_co+...%Assumptions for an intern
column with intern Walls over it.
      s1*s2*(q_Nk_S+(n_st_o-1)*(q_Nk_resid*usage_resid+q_Nk_office*usage_office));
```

```
   N_d_co=1.35*((n_st_o-1)*(s1*s2*(h_sl*g_0k_sl+g_Ak_sl)+...
      (s1+s2)*L_co*g_Ak_co)+sum(V_co(1:n_st_o))*g_0k_co)+...%Assumptions for an intern
column with intern Walls over it.
      1.5*(s1*s2*(q_Nk_S+(n_st_o-1)*(q_Nk_resid*usage_resid+q_Nk_office*usage_office)));

   %Compression
   sigma_c_d=N_d_co/A_co(n_st_o)/1000;%brought to N/mm^2
end

%Display Variables
%columnsvar_names = {'f_c_d' 'N/mm^2';'h_co' 'm';'b_co' 'm';'L_co' 'm';'A_co' 'm^2';'g_0k_co'
'kN/m^3';'g_Ak_co' 'kN/m^2';'N_k_co' 'kN';'N_d_co' 'kN';'sigma_c_d' 'N/mm^2   (must be <
f_c_0_d)';'f_c_d' 'N/mm^2'};
%columnsvar_values =
[f_c_d,h_co,b_co,L_co,A_co(n_st_o),g_0k_co,g_Ak_co,N_k_co,N_d_co,sigma_c_d,f_c_d];

%disp(char({'';'';'Final Values Columns';''}))

%for i = 1:size(columnsvar_names,1)
   %disp([char(columnsvar_names(i,1)),' = ', num2str(columnsvar_values(i)),'  ',
char(columnsvar_names(i,2))])
%end
%disp(char(' '))
%disp([char('h_co'),' = ', num2str(h_co),'  ', char('m')])
%disp([char('b_co'),' = ', num2str(b_co),'  ', char('m')])
%disp([char('Armature content'),' = ', num2str(P_co),'  ', char('-')])
%disp(char(' '))

%Volumes
%1 Story
nrcolumns=7;
nrwalls=1;
%n Stories
Nrcolumns=nrcolumns*n_st_o;
Nrwalls=nrwalls*n_st_o;
%1 Story
volumecolumns_concrete(n_st_o)=b_co*h_co*L_co*nrcolumns;
volumewalls_concrete(n_st_o)=5*h_co*L_co*nrwalls;%5m long walls, with the same width as the
columns
volumecolumns_armature(n_st_o)=volumecolumns_concrete(n_st_o)*P_co;
volumewalls_armature(n_st_o)=volumewalls_concrete(n_st_o)*P_co;
volumevertical_concrete(n_st_o)=volumecolumns_concrete(n_st_o)+volumewalls_concrete(n_st_
o);
volumevertical_armature(n_st_o)=volumecolumns_armature(n_st_o)+volumewalls_armature(n_st_
o);
%n Stories
volumecolumns_concrete_tot(n_st_o)=sum(volumecolumns_concrete(1:n_st_o));
volumewalls_concrete_tot(n_st_o)=sum(volumewalls_concrete(1:n_st_o));
volumecolumns_armature_tot(n_st_o)=sum(volumecolumns_armature(1:n_st_o));
volumewalls_armature_tot(n_st_o)=sum(volumewalls_armature(1:n_st_o));
volumevertical_concrete_tot(n_st_o)=sum(volumevertical_concrete(1:n_st_o));
volumevertical_armature_tot(n_st_o)=sum(volumevertical_armature(1:n_st_o));

% %Display Volumes
% volumecolumnsvar_names = {'Nr. columns' 'each story';
%    'Nr. walls' 'each story';
```

```
%     'Nr. columns' 'over whole building';
%     'Nr. walls' 'over whole building';
%     'Volume concrete' 'm^3    this story';
%     'Volume armature' 'm^3    this story';
%     'Total Volume concrete' 'm^3    over whole building';
%     'Total Volume armature' 'm^3    over whole building'};
% volumecolumnsvar_values = [nrcolumns,nrwalls,Nrcolumns,Nrwalls,...
%     volumevertical_concrete(n_st_o),volumevertical_armature(n_st_o),...
%      volumevertical_concrete_tot(n_st_o),volumevertical_armature_tot(n_st_o)];

%disp(char({'';'Total Nr. and Volume of Columns';''}))

%for i = 1:size(volumecolumnsvar_names,1)
   %disp([char(volumecolumnsvar_names(i,1)),' = ', num2str(volumecolumnsvar_values(i)),'  ',
char(volumecolumnsvar_names(i,2))])
%end
%disp(char(' '))
%% Foundation
%Material Properties
%Ground
sigma_bd=700;%in kN/m^2;for C_c = 30%, compression ground
%Concrete
sortofmaterialfoundation='concrete';
strengthclassfoundation='C25/30';
if strcmp(strengthclassfoundation,'C25/30')
   f_cd=16.5;%connect this value to the material with if function.
else%C30/37
   f_cd=20;
end

%Profile
%b_co and h_co of cross-section known
P_found=0.03;%armature content
t_plate_concrete=0.1;%10cm of concrete for closing of flat foundation for isolation
t_found=t_plate_concrete;%total thickness under columns
s_found=h_co+2*t_found;%Force propagation 1:1
f_found=s_found;%width of local foundation, f_found > s_found

%Compression
%Ground
sigma_b_d=N_d_co/(f_found*f_found);%in kN/m^2, concentrated foundation for fxf
%sigma_bd must be greater than sigma_b_d
%this should get the help of the plate compression, else the foundation has
%to get ways to big

%Concrete
%sigma_c_d=N_d_co/A_co/1000; must be smaller sigma_c_Rd
%SIA262,4.2.1.10 with 1:2 compression propagation resistence
k_c_found(n_st_o)=1/sqrt((h_co*b_co)/((h_co+t_found)*(b_co+t_found)));
if k_c_found(n_st_o) > 3
   disp(char(''))
   disp(char('k_c_found > 3 (SIA 262,(32))'));%if k_c_found > 3, the compression of the concrete is
too high -> either bigger foundation or more columns, for less compression
   break;
end
sigma_c_Rd=k_c_found(n_st_o)*f_cd;%k_c_found contains distribution of force
```

```
%Display Variables
%foundationvar_names = {'sigma_bd' 'kN/m^2';'f_cd' 'N/mm^2'; 'b_co' 'm'; 'h_co' 'm';'A_co'
'm^2';'t_plate_concrete' 'm';'t_found' 'm';'s_found' 'm';'f_found' 'm';'N_d_co' 'kN'; 'sigma_b_d' 'kN/m`
(must be < sigma_bd)'; 'sigma_bd' 'kN/m`';'k_c_found' '-'; 'sigma_c_d' 'N/mm^2   (must be <
sigma_c_Rd)';'sigma_c_Rd' 'N/mm^2'};
%foundationvar_values =
[sigma_bd,f_cd,b_co,h_co,A_co(n_st_o),t_plate_concrete,t_found,s_found,f_found,N_d_co,sigma
_b_d,sigma_bd,k_c_found,sigma_c_d,sigma_c_Rd];

%disp(char(' '))
%disp(char(' '))
%disp('Foundation verification')
%disp(['Material:',' ','Concrete'])
%disp(['Strength Class:',' ',char(strengthclassfoundation)])
%disp(char({'';'';'Start Values Foundation';''}))

%for i = 1:size(foundationvar_names,1)
   %disp([char(foundationvar_names(i,1)),' = ', num2str(foundationvar_values(i)),'  ',
char(foundationvar_names(i,2))])
%end
%disp(char(' '))
%disp([char('h_co'),' = ', num2str(h_co),'  ', char('m')])
%disp([char('b_co'),' = ', num2str(b_co),'  ', char('m')])
%disp([char('t_plate_concrete'),' = ', num2str(t_plate_concrete),'  ', char('m')])
%disp([char('t_found'),' = ', num2str(t_found),'  ', char('m')])
%disp([char('s_found'),' = ', num2str(s_found),'  ', char('m')])
%disp([char('f_found'),' = ', num2str(f_found),'  ', char('m')])
%disp([char('Armature content'),' = ', num2str(P_found),'  ', char('-')])
%disp(char(' '))

%Micropiles eventually
l_micropiles=0.5*H;%approximation of half of building height
d_micropiles=0.100;
t_micropiles=0.010;
nr_micropiles=0;
N_Rd_micropiles=l_micropiles*d_micropiles*pi*sigma_bd*nr_micropiles;

while sigma_c_d > sigma_c_Rd || sigma_b_d > sigma_bd
   if (t_found < 0.6) && (f_found < 2.0)
      %Profile
      %b_co and h_co of cross-section known
      t_found=t_found + 0.01;%total thickness under columns
      s_found=h_co+2*t_found;%Force propagation 1:1
      f_found=s_found;%width of local foundation, f_found > s_found

      %Compression
      %Ground
      sigma_b_d=N_d_co/(f_found*f_found);%in kN/m^2, concentrated foundation for fxf
      %sigma_bd must be greater than sigma_b_d
      %this should get the help of the plate compression, else the foundation has
      %to get ways to big

      %Concrete
      %sigma_c_d=N_d_co/A_co/1000; must be smaller sigma_c_Rd
      %SIA262,4.2.1.10 with 1:2 compression propagation resistence
```

```
        k_c_found(n_st_o)=1/sqrt((h_co*b_co)/((h_co+t_found)*(b_co+t_found)));
        if k_c_found(n_st_o) > 3
            disp(char(''))
            disp(char('k_c_found > 3 (SIA 262,(32))'));%if k_c_found > 3, the compression of the concrete is too high -> either bigger foundation or more columns, for less compression
            break;
        end
        sigma_c_Rd=k_c_found(n_st_o)*f_cd;%k_c_found contains distribution of force
    else
        %Profile
        %b_co and h_co of cross-section known
        t_found=min(0.6,(2-h_co)/2);%t_founs so, that s_found doesn't get bigger than 2m
        s_found=h_co+2*t_found;%Force propagation 1:1
        f_found=2;%2m x 2m foundation foot enabling enough place for micropiles

        %Compression
        %Ground
        nr_micropiles=max(nr_micropiles+1,4);%4 minimal amount of micropiles because of the high costs of work
        N_Rd_micropiles=l_micropiles*d_micropiles*pi*sigma_bd*0.1*nr_micropiles;%tau = 10% of sigma
        N_d_b_d=max(0,N_d_co - N_Rd_micropiles);
        sigma_b_d=N_d_b_d/(f_found*f_found);%in kN/m^2, concentrated foundation for fxf
        %sigma_bd must be greater than sigma_b_d
        %this should get the help of the plate compression, else the foundation has
        %to get ways to big

        %Concrete
        %sigma_c_d=N_d_co/A_co/1000; must be smaller sigma_c_Rd
        %SIA262,4.2.1.10 with 1:2 compression propagation resistence
        k_c_found(n_st_o)=1/sqrt((h_co*b_co)/((h_co+t_found)*(b_co+t_found)));
        if k_c_found(n_st_o) > 3
            disp(char(''))
            disp(char('k_c_found > 3 (SIA 262,(32))'));%if k_c_found > 3, the compression of the concrete is too high -> either bigger foundation or more columns, for less compression
            break;
        end
        sigma_c_Rd=k_c_found(n_st_o)*f_cd;%k_c_found contains distribution of force
    end
end
nr_micropiles_tot(n_st_o)=nr_micropiles*9;%7columns and 1 wall like 9 columns

%Display Variables
%foundationvar_names = {'sigma_bd' 'kN/m^2';'f_cd' 'N/mm^2'; 'b_co' 'm'; 'h_co' 'm';'A_co' 'm^2';'t_plate_concrete' 'm';'t_found' 'm';'s_found' 'm';'f_found' 'm';'N_d_co' 'kN'; 'sigma_b_d' 'kN/m` (must be < sigma_bd)'; 'sigma_bd' 'kN/m`';'k_c_found' '-'; 'sigma_c_d' 'N/mm^2   (must be < sigma_c_Rd)';'sigma_c_Rd' 'N/mm^2'};
%foundationvar_values = [sigma_bd,f_cd,b_co,h_co,A_co(n_st_o),t_plate_concrete,t_found,s_found,f_found,N_d_co,sigma_b_d,sigma_bd,k_c_found,sigma_c_d,sigma_c_Rd];

%disp(char({'';'';'Final Values Foundation';''}))

%for i = 1:size(foundationvar_names,1)
    %disp([char(foundationvar_names(i,1)),' = ', num2str(foundationvar_values(i)),'  ', char(foundationvar_names(i,2))])
```

```
%end
%disp(char(' '))
%disp([char('h_co'),' = ', num2str(h_co),'  ', char('m')])
%disp([char('t_plate_concrete'),' = ', num2str(t_plate_concrete),'  ', char('m')])
%disp([char('t_found'),' = ', num2str(t_found),'  ', char('m')])
%disp([char('s_found'),' = ', num2str(s_found),'  ', char('m')])
%disp([char('f_found'),' = ', num2str(f_found),'  ', char('m')])
%disp([char('Armature content'),' = ', num2str(P_found),'  ', char('-')])
%disp(char(' '))


%Volumes
volumeplate=t_plate_concrete*(B+2*(s_found/2-h_co/2))*(L+2*(s_found/2-h_co/2));
volumeconcentratedfoundation=(t_found-t_plate_concrete)*f_found*f_found*nrcolumns;%under
columns
volumeribbonfoundation=(t_found-t_plate_concrete)*f_found*5;%ribbon foundation under wall
volumefoundation_concrete(n_st_o)=volumeplate + volumeconcentratedfoundation +
volumeribbonfoundation;
volumefoundation_armature(n_st_o)=volumefoundation_concrete(n_st_o)*P_found;
volumemicropiles(n_st_o)=l_micropiles*(d_micropiles^2-
t_micropiles^2)*pi/4*nr_micropiles_tot(n_st_o);

%Display Volumes
%disp([char('Volume plate'),' = ', num2str(volumeplate),'  ', char('m^3')])
%disp([char('Volume concentrated foundation'),' = ', num2str(volumeconcentratedfoundation),'  ',
char('m^3')])
%disp([char('Volume ribbon foundation'),' = ', num2str(volumeribbonfoundation),'  ', char('m^3')])
%disp([char('Tot. Volume foundation'),' = ', num2str(volumefoundation_concrete(n_st_o)),'  ',
char('m^3')])
%disp([char('Tot. Volume armature'),' = ', num2str(volumefoundation_armature(n_st_o)),'  ',
char('m^3')])
%disp(char(' '))
%% Summary
%Display Variables
disp(char(' '))
disp(char(' '))
disp(char('Summary of Values'))
disp(char(' '))
disp(char('SCHEME 1 : In-situ cast concrete columns and wall supporting a reinforcing concrete
plate'))
disp([char('Nr. stories'),' = ', num2str(n_st_o)])
disp(char(' '))
disp(char('Slab'))
disp([char('Material'),': ', num2str(sortofmaterialslab),', ', num2str(strengthclassslab)])
slab_names = {'h_sl' 'm';'Armature content' '-';'Total Volume concrete' 'm^3    in whole
building';'Total Volume armature' 'm^3    in whole building'};
slab_values = [h_sl,P_sl,volumeslab_concrete_tot(n_st_o),volumeslab_armature_tot(n_st_o)];
% for i = 1:size(slab_values,2)
%     disp([char(slab_names(i,1)),' = ', num2str(slab_values(i)),'  ', char(slab_names(i,2))])
% end
for i = 1:size(slab_values,2)%only values output
    disp(num2str(slab_values(i)))
end
disp(char(' '))
disp(char('Columns'))
disp([char('Material'),': ', num2str(sortofmaterialcolumn),', ', num2str(strengthclasscolumn)])
```

```
columns_names = {'b_co' 'm';'h_co' 'm';'Armature content' '-';'Total Volume concrete' 'm^3    in
whole building';'Total Volume armature' 'm^3    in whole building'};
columns_values =
[b_co,h_co,P_co,volumevertical_concrete_tot(n_st_o),volumevertical_armature_tot(n_st_o)];
% for i = 1:size(columns_values,2)
%     disp([char(columns_names(i,1)),' = ', num2str(columns_values(i)),'  ',
char(columns_names(i,2))])
% end
for i = 1:size(columns_values,2)%only values output
   disp(num2str(columns_values(i)))
end
disp(char(' '))
disp(char('Foundation'))
disp([char('Material'),': ', 'Concrete' ,', ', num2str(strengthclassfoundation)])
foundation_names = {'t_plate_concrete' 'm';'t_found' 'm';'s_found' 'm';'f_found' 'm'; 'Armature
content' '-'; 'Volume plate' 'm^3';'Volume concentrated foundation' 'm^3';'Volume ribbon foundation'
'm^3';'Tot. Volume foundation' 'm^3';'Tot. Volume armature' 'm^3'; 'Tot Number Micropiles (steel)'
'm^3';'Tot. Volume Micropiles (steel)' 'm^3'};
foundation_values =
[t_plate_concrete,t_found,s_found,f_found,P_found,volumeplate,volumeconcentratedfoundation,vo
lumeribbonfoundation,volumefoundation_concrete(n_st_o),volumefoundation_armature(n_st_o),nr
_micropiles_tot(n_st_o),volumemicropiles(n_st_o)];
% for i = 1:size(foundation_values,2)
%     disp([char(foundation_names(i,1)),' = ', num2str(foundation_values(i)),'  ',
char(foundation_names(i,2))])
% end
for i = 1:size(foundation_values,2)%only values output
   disp(num2str(foundation_values(i)))
end
disp(char(' '))
disp(char(' '))
disp(char('TOTAL Materials'))
volume_armature_overground_tot(n_st_o)=volumeslab_armature_tot(n_st_o)+volumevertical_arm
ature_tot(n_st_o);
TOTAL_names = {'Tot. Volume Concrete over ground C25/30' 'm^3';'Tot. Volume Concrete over
ground C30/37' 'm^3';'Tot. Volume Concrete foundation C25/30' 'm^3';'Tot. Volume Armature over
ground' 'm^3';'Tot. Volume Armature foundation' 'm^3';'Tot. Volume Micropiles (steel)' 'm^3'};
TOTAL_values =
[volumeslab_concrete_tot(n_st_o),volumevertical_concrete_tot(n_st_o),volumefoundation_concret
e(n_st_o),volume_armature_overground_tot(n_st_o),volumefoundation_armature(n_st_o),volume
micropiles(n_st_o)];
% for i = 1:size(TOTAL_values,2)
%     disp([char(TOTAL_names(i,1)),' = ', num2str(TOTAL_values(i)),'  ', char(TOTAL_names(i,2))])
% end
for i = 1:size(TOTAL_values,2)%only values output
   disp(num2str(TOTAL_values(i)))
end
disp(char(' '))
usageratio_overground_C2530(n_st_o) = volumeslab_concrete_tot(n_st_o)/(B*L*n_st_o);
usageratio_overground_C3037(n_st_o) = volumevertical_concrete_tot(n_st_o)/(B*L*n_st_o);
usageratio_foundation(n_st_o) = volumefoundation_concrete(n_st_o)/(B*L*n_st_o);
usageratio_armature_overground_tot(n_st_o)=volume_armature_overground_tot(n_st_o)/(B*L*n_s
t_o);
usageratio_armature_underground_tot(n_st_o)=volumefoundation_armature(n_st_o)/(B*L*n_st_o);
usageratio_micropiles(n_st_o)=volumemicropiles(n_st_o)/(B*L*n_st_o);
```

```
usageratio_concrete_tot(n_st_o)=usageratio_overground_C2530(n_st_o)+usageratio_overground
_C3037(n_st_o)+usageratio_foundation(n_st_o);
usage_names = {'Usage ratio Concrete over ground C25/30' 'm^3/m^2 RES';'Usage ratio over
ground Concrete C30/37' 'm^3/m^2 RES';'Usage ratio Concrete foundation C25/30' 'm^3/m^2
RES';'Usage ratio Armature over ground' 'm^3/m^2 RES';'Usage ratio Armature foundation'
'm^3/m^2 RES';'Usage ratio Micropiles (steel)' 'm^3/m^2 RES'};
usage_values =
[usageratio_overground_C2530(n_st_o),usageratio_overground_C3037(n_st_o),usageratio_found
ation(n_st_o),usageratio_armature_overground_tot(n_st_o),usageratio_armature_underground_tot
(n_st_o),usageratio_micropiles(n_st_o)];
% for i = 1:size(usage_values,2)
%     disp([char(usage_names(i,1)),' = ', num2str(usage_values(i)),'  ', char(usage_names(i,2))])
% end
for i = 1:size(usage_values,2)%only values output
   disp(num2str(usage_values(i)))
end

usageratio_impact_concrete(n_st_o)=227.70*usageratio_overground_C3037(n_st_o)+170.2*(usag
eratio_overground_C2530(n_st_o)+usageratio_foundation(n_st_o));
usageratio_impact_steel(n_st_o)=5353.7*(usageratio_armature_overground_tot(n_st_o)+usagerati
o_armature_underground_tot(n_st_o)+usageratio_micropiles(n_st_o));
usageratio_impact_tot(n_st_o)=usageratio_impact_concrete(n_st_o)+usageratio_impact_steel(n_s
t_o);
end
figure(11);
plot(1:maxstories,volumeslab_concrete_tot)
hold on;
plot(1:maxstories,volumeslab_armature_tot)
plot(1:maxstories,volumevertical_concrete_tot)
plot(1:maxstories,volumevertical_armature_tot)
plot(1:maxstories,volumefoundation_concrete)
plot(1:maxstories,volumefoundation_armature)
plot(1:maxstories,volumemicropiles)
legend('slabs concrete','slabs armature','columns concrete',...
   'columns armature','foundation concrete','foundation armature','Volume Micropiles (steel)')
xlabel('number of stories')
ylabel('volume [m^3]')
xlim([1 14])
ylim([-100 350])
annotation('doublearrow',[0.13,0.31],[0.24,0.24])
annotation('textbox',[0.17,0.24,0.1,0],'string','SFH & TH')
annotation('doublearrow',[0.13,0.545],[0.2,0.2])
annotation('textbox',[0.29,0.2,0.05,0],'string','MFH')
annotation('doublearrow',[0.13,0.905],[0.16,0.16])
annotation('textbox',[0.47,0.16,0.04,0],'string','AB')
hold off;
saveas(gcf,'images/Matlab plots/11_cem_totvolumes_all','epsc')
figure(12);
plot(1:maxstories,volumeslab_concrete_tot)
hold on;
plot(1:maxstories,volumevertical_concrete_tot)
plot(1:maxstories,volumefoundation_concrete)
plot(1:maxstories,volume_armature_overground_tot)
plot(1:maxstories,volumefoundation_armature)
plot(1:maxstories,volumemicropiles)
```

```
legend('Total volume Concrete over ground C25/30','Total volume Concrete over ground C30/37','Total volume Concrete foundation','Total volume Armature over ground','Total volume Armature foundation','Volume Micropiles (steel)')
xlabel('number of stories')
ylabel('volume [m^3]')
xlim([1 14])
ylim([-100 350])
annotation('doublearrow',[0.13,0.31],[0.24,0.24])
annotation('textbox',[0.17,0.24,0.1,0],'string','SFH & TH')
annotation('doublearrow',[0.13,0.545],[0.2,0.2])
annotation('textbox',[0.29,0.2,0.05,0],'string','MFH')
annotation('doublearrow',[0.13,0.905],[0.16,0.16])
annotation('textbox',[0.47,0.16,0.04,0],'string','AB')
hold off;
saveas(gcf,'images/Matlab plots/12_ cem _totvolumes_summary','epsc')
figure(13);
plot(1:maxstories,usageratio_overground_C2530)
hold on;
plot(1:maxstories,usageratio_overground_C3037)
plot(1:maxstories,usageratio_foundation)
plot(1:maxstories,usageratio_armature_overground_tot)
plot(1:maxstories,usageratio_armature_underground_tot)
plot(1:maxstories,usageratio_micropiles)
plot(1:maxstories,usageratio_concrete_tot)
legend('Total ratio Concrete over ground C25/30','Total ratio Concrete over ground C30/37','Total ratio Concrete foundation C25/30','Total ratio Armature over ground','Total ratio Armature foundation','ratio Micropiles (steel)','ratio TOTAL concrete')
xlabel('number of stories')
ylabel('ratio [m^3/m^2 RES]')
xlim([1 14])
ylim([-0.15 0.5])
annotation('doublearrow',[0.13,0.31],[0.24,0.24])
annotation('textbox',[0.17,0.24,0.1,0],'string','SFH & TH')
annotation('doublearrow',[0.13,0.545],[0.2,0.2])
annotation('textbox',[0.29,0.2,0.05,0],'string','MFH')
annotation('doublearrow',[0.13,0.905],[0.16,0.16])
annotation('textbox',[0.47,0.16,0.04,0],'string','AB')
hold off;
saveas(gcf,'images/Matlab plots/13_ cem _RES','epsc')
figure(14);
plot(1:maxstories,usageratio_impact_concrete)
hold on;
plot(1:maxstories,usageratio_impact_steel)
plot(1:maxstories,usageratio_impact_tot)
legend('ratio concrete impact','ratio steel impact','ratio total building impact')
xlabel('number of stories')
ylabel('ratio impact [kg CO_2 / m^2 RES]')
xlim([1 14])
ylim([-10 150])
annotation('doublearrow',[0.13,0.31],[0.24,0.24])
annotation('textbox',[0.17,0.24,0.1,0],'string','SFH & TH')
annotation('doublearrow',[0.13,0.545],[0.2,0.2])
annotation('textbox',[0.29,0.2,0.05,0],'string','MFH')
annotation('doublearrow',[0.13,0.905],[0.16,0.16])
annotation('textbox',[0.47,0.16,0.04,0],'string','AB')
hold off;
```

```
saveas(gcf,'images/Matlab plots/14_ cem _CO2impact','epsc')
%% SCHEMA 2 : Platform timber frame prefab load-bearing walls and beams
maxstories = 8;
k_c_found = zeros(maxstories,1);
volumebeams_tot = zeros(maxstories,1);
volumepavement = zeros(maxstories,1);
volumehorizontal = zeros(maxstories,1);
volumecolumns = zeros(maxstories,1);
volumesupports = zeros(maxstories,1);
volumevertical = zeros(maxstories,1);
volumepannels = zeros(maxstories,1);
volume_solidwood_tot = zeros(maxstories,1);
volumefoundation_concrete = zeros(maxstories,1);
volumefoundation_armature = zeros(maxstories,1);
usageratio_pannels = zeros(maxstories,1);
usageratio_solidwood_tot = zeros(maxstories,1);
usageratio_foundation_concrete = zeros(maxstories,1);
usageratio_foundation_armature = zeros(maxstories,1);
usageratio_impact_solid = zeros(maxstories,1);
usageratio_impact_OSB = zeros(maxstories,1);
usageratio_impact_concrete = zeros(maxstories,1);
usageratio_impact_steel = zeros(maxstories,1);
usageratio_impact_tot = zeros(maxstories,1);
for n_st_o=1:maxstories
%% Building Geometry
H_st=2.7;%Intern Height of story
L=10;
B=10;
H=n_st_o*(H_st+0.5);%30cm of suspended ceiling and 40cm/2 of Floor Beams height
s1=5;% s1 => s2 , as the static verifications are apported on the length of s1
s2=0.625;%spacing of little columns
usage_resid=1;
usage_office=0;

%Display Variables
geometryvar_names = {'n_st_o' 'stories';
    'H_st' 'm';
    'L' 'm';
    'B' 'm';
    'H' 'm';
    's1' 'm';
    's2' 'm';
    'usage_resid' '(1=100%)';
    'usage_office' '(1=100%)'};
geometryvar_values = [n_st_o,H_st,L,B,H,s1,s2,...
    usage_resid,usage_office];

disp(char(' '))
disp(char(' '))
disp(char(' '))
disp(char({'Building Geometry';''}))

for i = 1:size(geometryvar_names,1)
    disp([char(geometryvar_names(i,1)),' = ', num2str(geometryvar_values(i)),'  ',
char(geometryvar_names(i,2))])
end
```

```
%% Beams
%Material Properties
sortofmaterialbeam='solid';%solid or glulam wood, concrete in a second moment
strengthclassbeam='C24';
if strcmp(sortofmaterialbeam,'solid')%Solid C24
   f_m_d=14;%connect this value to the material with if function.
else%Glulam GL24h
   f_m_d=16;
end

%Bending Resistence verification
%Profile
b_be=0.06;
h_be=0.01;%ev. minimal thickness of ceiling
A_be=b_be*h_be;
W_be=b_be*h_be^2/6;

%Loads
g_0k_be=5;
g_Ak_be=1;%Assumption of 100kg/m^2
q_Nk_S=2.5;
q_Nk_resid=2;
q_Nk_office=3;
q_k_be_s1=A_be*g_0k_be+s2*(g_Ak_be+max(q_Nk_S,q_Nk_resid*usage_resid+q_Nk_office*usa
ge_office));
q_k_be_s2=A_be*g_0k_be+s1*(g_Ak_be+max(q_Nk_S,q_Nk_resid*usage_resid+q_Nk_office*usa
ge_office));
q_d_be_s1=1.35*(A_be*g_0k_be+s2*g_Ak_be)+1.5*s2*max(q_Nk_S,q_Nk_resid*usage_resid+q_
Nk_office*usage_office);
q_d_be_s2=1.35*(A_be*g_0k_be+s1*g_Ak_be)+1.5*s1*max(q_Nk_S,q_Nk_resid*usage_resid+q_
Nk_office*usage_office);
m_d_be_s1=q_d_be_s1*s1^2/8;
m_d_be_s2=q_d_be_s2*s2^2/8;

%Bending resistence (SIA 265,(14))
sigma_m_d_be_s1=m_d_be_s1/W_be/1000;%brought to N/mm^2
sigma_m_d_be_s2=m_d_be_s2/W_be/1000;%brought to N/mm^2

%Display Variables
%beamsvar_names = {'f_m_d' 'N/mm^2';'b_be' 'm';'h_be' 'm';'A_be' 'm^2';'W_be' 'm^3';'g_0k_be'
'kN/mm^3';'g_Ak_be' 'kN/mm^2'; 'q_Nk_S' 'kN/mm^2';'q_Nk_resid' 'kN/mm^2';'q_Nk_office'
'kN/mm^2';'q_k_be_s1' 'kN/m';'q_k_be_s2' 'kN/m';'q_d_be_s1' 'kN/m';'q_d_be_s2'
'kN/m';'m_d_be_s1' 'kNm';'m_d_be_s2' 'kNm';'sigma_m_d_be_s1' 'N/mm^2   (must be < f_m_d)';
'sigma_m_d_be_s2' 'N/mm^2   (must be < f_m_d)';'f_m_d' 'N/mm^2'};
%beamsvar_values =
[f_m_d,b_be,h_be,A_be,W_be,g_0k_be,g_Ak_be,q_Nk_S,q_Nk_resid,q_Nk_office,q_k_be_s1,q_k
_be_s2,q_d_be_s1,q_d_be_s2,m_d_be_s1,m_d_be_s2,sigma_m_d_be_s1,sigma_m_d_be_s2,f_
m_d];

%disp(char(' '))
%disp(char(' '))
%disp('Beams verification')
%disp(['Material:',' ',char(sortofmaterialbeam)])
%disp(['Strength Class:',' ',char(strengthclassbeam)])
%disp(char({'';'';'Start Bending Values Beams';''}))
```

```
%for i = 1:size(beamsvar_names,1)
   %disp([char(beamsvar_names(i,1)),' = ', num2str(beamsvar_values(i)),'  ',
char(beamsvar_names(i,2))])
%end
%disp(char(' '))
%disp([char('b_be'),' = ', num2str(b_be),'  ', char('m')])
%disp([char('h_be'),' = ', num2str(h_be),'  ', char('m')])
%disp(char(' '))

while sigma_m_d_be_s1 > f_m_d
    h_be = h_be + 0.01;
    A_be=b_be*h_be;
    W_be=b_be*h_be^2/6;

    %Loads     (own Weight could be neglected for static)
    g_0k_be=5;
    g_Ak_be=1;%Assumption of 100kg/m^2
    q_Nk_S=2.5;
    q_Nk_resid=2;
    q_Nk_office=3;

q_k_be_s1=A_be*g_0k_be+s2*(g_Ak_be+max(q_Nk_S,q_Nk_resid*usage_resid+q_Nk_office*usa
ge_office));

q_k_be_s2=A_be*g_0k_be+s1*(g_Ak_be+max(q_Nk_S,q_Nk_resid*usage_resid+q_Nk_office*usa
ge_office));

q_d_be_s1=1.35*(A_be*g_0k_be+s2*g_Ak_be)+1.5*s2*max(q_Nk_S,q_Nk_resid*usage_resid+q_
Nk_office*usage_office);

q_d_be_s2=1.35*(A_be*g_0k_be+s1*g_Ak_be)+1.5*s1*max(q_Nk_S,q_Nk_resid*usage_resid+q_
Nk_office*usage_office);
    m_d_be_s1=q_d_be_s1*s1^2/8;
    m_d_be_s2=q_d_be_s2*s2^2/8;

    %Bending resistence (SIA 265,(14))
    sigma_m_d_be_s1=m_d_be_s1/W_be/1000;%brought to N/mm^2
    sigma_m_d_be_s2=m_d_be_s2/W_be/1000;%brought to N/mm^2
end

%Display Variables
%beamsvar_names = {'f_m_d' 'N/mm^2';'b_be' 'm';'h_be' 'm';'A_be' 'm^2';'W_be' 'm^3';'g_0k_be'
'kN/mm^3';'g_Ak_be' 'kN/mm^2'; 'q_Nk_S' 'kN/mm^2';'q_Nk_resid' 'kN/mm^2';'q_Nk_office'
'kN/mm^2';'q_k_be_s1' 'kN/m';'q_k_be_s2' 'kN/m';'q_d_be_s1' 'kN/m';'q_d_be_s2'
'kN/m';'m_d_be_s1' 'kNm';'m_d_be_s2' 'kNm';'sigma_m_d_be_s1' 'N/mm^2   (must be < f_m_d)';
'sigma_m_d_be_s2' 'N/mm^2   (must be < f_m_d)';'f_m_d' 'N/mm^2'};
%beamsvar_values =
[f_m_d,b_be,h_be,A_be,W_be,g_0k_be,g_Ak_be,q_Nk_S,q_Nk_resid,q_Nk_office,q_k_be_s1,q_k
_be_s2,q_d_be_s1,q_d_be_s2,m_d_be_s1,m_d_be_s2,sigma_m_d_be_s1,sigma_m_d_be_s2,f_
m_d];

%disp(char({'';'';'Final Bending Values Beams';''}))

%for i = 1:size(beamsvar_names,1)
   %disp([char(beamsvar_names(i,1)),' = ', num2str(beamsvar_values(i)),'  ',
char(beamsvar_names(i,2))])
```

```
%end
%disp(char(' '))
%disp([char('b_be'),' = ', num2str(b_be),'  ', char('m')])
%disp([char('h_be'),' = ', num2str(h_be),'  ', char('m')])
%disp(char(' '))


%Deflection verification
%h_be taken from bending resistence verification,ev. further reduced if deflection is determinating
%Material Properties
if strcmp(sortofmaterialbeam,'solid')%Solid C24
   E_0_m=11000;%connect this value to the material with if function.
   phi=0.6;%humidity class 1
   E_0_phi=E_0_m/(1+phi);
else%Glulam GL24h (exact the same as solid wood, but this is a preparation for concrete
programming)
   E_0_m=11000;
   phi=0.6;%humidity class 1
   E_0_phi=E_0_m/(1+phi);
end

%Profile
A_be=b_be*h_be;
I_be=b_be*h_be^3/12;%in m^4

%Loads (SIA 260,A,tab.2)
psi_1=0.5;%frequent
psi_2=0.3;%quasi-permanent
%rare
q_k_be_s1_rare=A_be*g_0k_be+s2*(g_Ak_be+...
   max(q_Nk_S,q_Nk_resid*usage_resid+q_Nk_office*usage_office));%only one payload acting
q_k_be_s2_rare=A_be*g_0k_be+s1*(g_Ak_be+...
   max(q_Nk_S,q_Nk_resid*usage_resid+q_Nk_office*usage_office));
%frequent
q_k_be_s1_fr=A_be*g_0k_be+s2*(g_Ak_be+...
   psi_1*max(q_Nk_S,q_Nk_resid*usage_resid+q_Nk_office*usage_office));%only one payload
acting
q_k_be_s2_fr=A_be*g_0k_be+s1*(g_Ak_be+...
   psi_1*max(q_Nk_S,q_Nk_resid*usage_resid+q_Nk_office*usage_office));
%quasi-permanent
q_k_be_s1_qp=A_be*g_0k_be+s2*(g_Ak_be+...
   psi_2*max(q_Nk_S,q_Nk_resid*usage_resid+q_Nk_office*usage_office));%only one payload
acting
q_k_be_s2_qp=A_be*g_0k_be+s1*(g_Ak_be+...
   psi_2*max(q_Nk_S,q_Nk_resid*usage_resid+q_Nk_office*usage_office));

%Deflections (SIA 260,A,tab.3)
%rare
w_be_s1_rare=5/384*q_k_be_s1_rare*s1^4/(E_0_phi*I_be);
w_be_s2_rare=5/384*q_k_be_s2_rare*s2^4/(E_0_phi*I_be);
w_be_s1_lim_rare=s1/0.500;
w_be_s2_lim_rare=s2/0.500;
%frequent
w_be_s1_fr=5/384*q_k_be_s1_fr*s1^4/(E_0_phi*I_be);
w_be_s2_fr=5/384*q_k_be_s2_fr*s2^4/(E_0_phi*I_be);
w_be_s1_lim_fr=s1/0.350;
```

```
w_be_s2_lim_fr=s2/0.350;
%quasi-permanent
w_be_s1_qp=5/384*q_k_be_s1_qp*s1^4/(E_0_phi*I_be);
w_be_s2_qp=5/384*q_k_be_s2_qp*s2^4/(E_0_phi*I_be);
w_be_s1_lim_qp=s1/0.300;
w_be_s2_lim_qp=s2/0.300;

%Display Variables
%beamsvar_names2 = {'E_0_m' 'N/mm^2';'phi' '-';'E_0_phi' 'N/mm^2';'b_be' 'm';'h_be' 'm';'I_be'
'm^4';'g_0k_be' 'N/mm^2';'g_Ak_be' 'N/mm^2';'q_Nk_S' 'N/mm^2';'q_Nk_resid'
'N/mm^2';'q_Nk_office' 'N/mm^2';'psi_1' '-';'psi_2' '-';'q_k_be_s1_rare' 'kN/m';'q_k_be_s2_rare'
'kN/m';'q_k_be_s1_fr' 'kN/m';'q_k_be_s2_fr' 'kN/m';'q_k_be_s1_qp' 'kN/m';'q_k_be_s2_qp'
'kN/m';'w_be_s1_rare' 'mm   (must be < w_be_s1_lim_rare)';'w_be_s2_rare' 'mm   (must be <
w_be_s2_lim_rare)';'w_be_s1_lim_rare' 'mm';'w_be_s2_lim_rare' 'mm';'w_be_s1_fr' 'mm   (must be
< w_be_s1_lim_fr)';'w_be_s2_fr' 'mm   (must be < w_be_s2_lim_fr)';'w_be_s1_lim_fr'
'mm';'w_be_s2_lim_fr' 'mm';'w_be_s1_qp' 'mm   (must be < w_be_s1_lim_qp)';'w_be_s2_qp' 'mm
(must be < w_be_s1_lim_qp)';'w_be_s1_lim_qp' 'mm';'w_be_s2_lim_qp' 'mm'};
%beamsvar_values2 =
[E_0_m,phi,E_0_phi,b_be,h_be,I_be,g_0k_be,g_Ak_be,q_Nk_S,q_Nk_resid,q_Nk_office,psi_1,psi
_2,q_k_be_s1_rare,q_k_be_s2_rare,q_k_be_s1_fr,q_k_be_s2_fr,q_k_be_s1_qp,q_k_be_s2_qp,w
_be_s1_rare,w_be_s2_rare,w_be_s1_lim_rare,w_be_s2_lim_rare,w_be_s1_fr,w_be_s2_fr,w_be_s
1_lim_fr,w_be_s2_lim_fr,w_be_s1_qp,w_be_s2_qp,w_be_s1_lim_qp,w_be_s2_lim_qp];

%disp(char({'';'';'Start Deflection Values Beams';''}))

%for i = 1:size(beamsvar_names2,1)
   %disp([char(beamsvar_names2(i,1)),' = ', num2str(beamsvar_values2(i)),'  ',
char(beamsvar_names2(i,2))])
%end
%disp(char(' '))
%disp([char('b_be'),' = ', num2str(b_be),'  ', char('m')])
%disp([char('h_be'),' = ', num2str(h_be),'  ', char('m')])
%disp(char(' '))

while (w_be_s1_rare > w_be_s1_lim_rare)||...
      (w_be_s1_fr > w_be_s1_lim_fr)||(w_be_s1_qp > w_be_s1_lim_qp)
   %Profile
   h_be = h_be + 0.01;
   A_be=b_be*h_be;
   I_be=b_be*h_be^3/12;%in m^4

   %Loads (SIA 260,A,tab.2)
   psi_1=0.5;%frequent
   psi_2=0.3;%quasi-permanent
   %rare
   q_k_be_s1_rare=A_be*g_0k_be+s2*(g_Ak_be+...
      max(q_Nk_S,q_Nk_resid*usage_resid+q_Nk_office*usage_office));%only one payload acting
   q_k_be_s2_rare=A_be*g_0k_be+s1*(g_Ak_be+...
      max(q_Nk_S,q_Nk_resid*usage_resid+q_Nk_office*usage_office));
   %frequent
   q_k_be_s1_fr=A_be*g_0k_be+s2*(g_Ak_be+...
      psi_1*max(q_Nk_S,q_Nk_resid*usage_resid+q_Nk_office*usage_office));%only one payload
acting
   q_k_be_s2_fr=A_be*g_0k_be+s1*(g_Ak_be+...
      psi_1*max(q_Nk_S,q_Nk_resid*usage_resid+q_Nk_office*usage_office));
   %quasi-permanent
```

```
    q_k_be_s1_qp=A_be*g_0k_be+s2*(g_Ak_be+...
        psi_2*max(q_Nk_S,q_Nk_resid*usage_resid+q_Nk_office*usage_office));%only one payload
acting
    q_k_be_s2_qp=A_be*g_0k_be+s1*(g_Ak_be+...
        psi_2*max(q_Nk_S,q_Nk_resid*usage_resid+q_Nk_office*usage_office));

    %Deflections (SIA 260,A,tab.3)
    %rare
    w_be_s1_rare=5/384*q_k_be_s1_rare*s1^4/(E_0_phi*I_be);
    w_be_s2_rare=5/384*q_k_be_s2_rare*s2^4/(E_0_phi*I_be);
    w_be_s1_lim_rare=s1/0.500;
    w_be_s2_lim_rare=s2/0.500;
    %frequent
    w_be_s1_fr=5/384*q_k_be_s1_fr*s1^4/(E_0_phi*I_be);
    w_be_s2_fr=5/384*q_k_be_s2_fr*s2^4/(E_0_phi*I_be);
    w_be_s1_lim_fr=s1/0.350;
    w_be_s2_lim_fr=s2/0.350;
    %quasi-permanent
    w_be_s1_qp=5/384*q_k_be_s1_qp*s1^4/(E_0_phi*I_be);
    w_be_s2_qp=5/384*q_k_be_s2_qp*s2^4/(E_0_phi*I_be);
    w_be_s1_lim_qp=s1/0.300;
    w_be_s2_lim_qp=s2/0.300;
end

%Display Variables
%beamsvar_names2 = {'E_0_m' 'N/mm^2';'phi' '-';'E_0_phi' 'N/mm^2';'b_be' 'm';'h_be' 'm';'I_be'
'm^4';'g_0k_be' 'N/mm^2';'g_Ak_be' 'N/mm^2';'q_Nk_S' 'N/mm^2';'q_Nk_resid'
'N/mm^2';'q_Nk_office' 'N/mm^2';'psi_1' '-';'psi_2' '-';'q_k_be_s1_rare' 'kN/m';'q_k_be_s2_rare'
'kN/m';'q_k_be_s1_fr' 'kN/m';'q_k_be_s2_fr' 'kN/m';'q_k_be_s1_qp' 'kN/m';'q_k_be_s2_qp'
'kN/m';'w_be_s1_rare' 'mm   (must be < w_be_s1_lim_rare)';'w_be_s2_rare' 'mm   (must be <
w_be_s2_lim_rare)';'w_be_s1_lim_rare' 'mm';'w_be_s2_lim_rare' 'mm';'w_be_s1_fr' 'mm   (must be
< w_be_s1_lim_fr)';'w_be_s2_fr' 'mm   (must be < w_be_s2_lim_fr)';'w_be_s1_lim_fr'
'mm';'w_be_s2_lim_fr' 'mm';'w_be_s1_qp' 'mm   (must be < w_be_s1_lim_qp)';'w_be_s2_qp' 'mm
(must be < w_be_s1_lim_qp)';'w_be_s1_lim_qp' 'mm';'w_be_s2_lim_qp' 'mm'};
%beamsvar_values2 =
[E_0_m,phi,E_0_phi,b_be,h_be,I_be,g_0k_be,g_Ak_be,q_Nk_S,q_Nk_resid,q_Nk_office,psi_1,psi
_2,q_k_be_s1_rare,q_k_be_s2_rare,q_k_be_s1_fr,q_k_be_s2_fr,q_k_be_s1_qp,q_k_be_s2_qp,w
_be_s1_rare,w_be_s2_rare,w_be_s1_lim_rare,w_be_s2_lim_rare,w_be_s1_fr,w_be_s2_fr,w_be_s
1_lim_fr,w_be_s2_lim_fr,w_be_s1_qp,w_be_s2_qp,w_be_s1_lim_qp,w_be_s2_lim_qp];

%disp(char({'';'';'Final Deflection Values Beams';''}))

%for i = 1:size(beamsvar_names2,1)
    %disp([char(beamsvar_names2(i,1)),' = ', num2str(beamsvar_values2(i)),'  ',
char(beamsvar_names2(i,2))])
%end
%disp(char(' '))
%disp([char('b_be'),' = ', num2str(b_be),'  ', char('m')])
%disp([char('h_be'),' = ', num2str(h_be),'  ', char('m')])
%disp(char(' '))

%Volumes
%1 Story
nrbeams_s1=min(L,B)/s1*(max(L,B)/s2+1);
%if s2 too small, beams only in one directions and hence the beams of length s2 should be
ignored
```

```
nrbeams_s2=max(L,B)/s2*(min(L,B)/s1+1);%2 outside for attachment insulation and 1 for every
middle support to close spaces between walls.
%n Stories
Nrbeams_s1=nrbeams_s1*n_st_o;
Nrbeams_s2=nrbeams_s2*n_st_o;

volumebeams_s1=b_be*h_be*Nrbeams_s1*s1;
volumebeams_s2=b_be*h_be*Nrbeams_s2*s2;
volumebeams_tot(n_st_o)=volumebeams_s1+volumebeams_s2;
volumepavement(n_st_o)=B*L*0.02*n_st_o;%2cm of pavement
volumehorizontal(n_st_o)=volumebeams_tot(n_st_o)+volumepavement(n_st_o);

%Display Volumes
%volumebeamsvar_names = {'nrbeams_s1' 'each story';'nrbeams_s2' 'each story';'Nrbeams_s1'
'over whole building';'Nrbeams_s2' 'over whole building';'Volume beams s1' 'm^3';'Volume beams
s2' 'm^3';'Total Volume beams' 'm^3'};
%volumebeamsvar_values =
[nrbeams_s1,nrbeams_s2,Nrbeams_s1,Nrbeams_s2,volumebeams_s1,volumebeams_s2,volumeb
eams_tot(n_st_o)];

%disp(char({'';'Total Nr. and Volume Beams';''}))

%for i = 1:size(volumebeamsvar_names,1)
    %disp([char(volumebeamsvar_names(i,1)),' = ', num2str(volumebeamsvar_values(i)),'  ',
char(volumebeamsvar_names(i,2))])
%end
%disp(char(' '))
%% Columns
%Material Properties
sortofmaterialcolumn='solid';%solid or glulam wood,concrete in a second moment
strengthclasscolumn='C24';
if strcmp(sortofmaterialcolumn,'solid')%Solid C24
    f_c_0_d=12;%connect this value to the material with if function.
else%Glulam GL24h
    f_c_0_d=14.5;
end

%Profile
b_co=0.06;%ev. dynamic profile dependent from maxstories
h_co=0.01;%ev. minimal profile height
L_co=3.2;%3.2m approximately of static length of columns
A_co=b_co*h_co;
V_co=A_co*L_co;
I_y_co=b_co*h_co^3/12;
I_z_co=h_co*b_co^3/12;
i_y_co=sqrt(I_y_co/A_co);
i_z_co=sqrt(I_z_co/A_co);

%Loads (own Weight could be neglected for static)
g_0k_co=5;%5kN/m^3
g_Ak_co=1;%Assumption 100kg/m^2
N_k_co_s1=(n_st_o-1)*((s1+s2)*A_be*g_0k_be+s1*s2*g_Ak_be+...
    V_co*g_0k_co+(s1+s2)*L_co*g_Ak_co)+...%Assumptions for an intern column with intern Walls
over it.
    s1*s2*(q_Nk_S+(n_st_o-1)*(q_Nk_resid*usage_resid+q_Nk_office*usage_office));
N_k_co_s2=N_k_co_s1;
```

```
N_d_co_s1=1.35*((n_st_o-1)*((s1+s2)*A_be*g_0k_be+s1*s2*g_Ak_be+...
    V_co*g_0k_co+(s1+s2)*L_co*g_Ak_co))+...%Assumptions for an intern column with intern Walls over it.
    1.5*(s1*s2*(q_Nk_S+(n_st_o-1)*(q_Nk_resid*usage_resid+q_Nk_office*usage_office)));
N_d_co_s2=N_d_co_s1;

%Compression & Buckling (SIA 265,(10)/(29))
bucklingfactor_y=1.0;%Pin supported
bucklingfactor_z=0.0;%Buckling restrained by OSB pannels along the wall
L_k_y_co=bucklingfactor_y*L_co;
L_k_z_co=bucklingfactor_z*L_co;
lambda_y=L_k_y_co/i_y_co;
lambda_z=L_k_z_co/i_z_co;
lambda=max(lambda_y,lambda_z);
if strcmp(sortofmaterialcolumn,'solid')
    lambda_rel=lambda/(18*pi);
    betha_c=0.2;
else
    lambda_rel=lambda/(20*pi);
    betha_c=0.1;
end
if lambda_rel < 0.3%control of minimal Slenderness
    buckling='lambda_rel < 0.3 -> no buckling';
else
    buckling='buckling to proof ';
end
k=0.5*(1+betha_c*(lambda_rel-0.3)+lambda_rel^2);
k_c=1/(k+sqrt(k^2-lambda_rel^2));
if k_c > 1
    disp(char(''))
    disp(char('k_c > 1 -> no buckling'));
    break;
end
sigma_c_0_k=N_k_co_s1/A_co/1000;%brought to N/mm^2
sigma_c_0_d=N_d_co_s1/A_co/1000;%brought to N/mm^2

%Display Variables
%columnsvar_names = {'f_c_0_d' 'N/mm^2';'b_co' 'm';'h_co' 'm';'L_co' 'm';'A_co' 'm^2';'V_co' 'm^3';'I_y_co' 'm^4';'I_z_co' 'm^4';'i_y_co' 'm';'i_z_co' 'm';'g_0k_co' 'kN/m^3';'g_Ak_co' 'kN/m^2';'N_k_co_s1' 'kN';'N_k_co_s2' 'kN';'N_d_co_s1' 'kN';'N_d_co_s2' 'kN';'bucklingfactor_y' '-';'bucklingfactor_z' '-';'L_k_y_co' 'm';'L_k_z_co' 'm';'lambda_y' '-';'lambda_z' '-';'lambda' '-';'lambda_rel' '-';'betha_c=0.2' '-';'k' '-';'k_c' '-';'sigma_c_0_d' 'N/mm^2   (must be < f_c_0_d)';'sigma_c_0_k' 'N/mm^2   (must be < k_c*f_c_0_d)';'f_c_0_d' 'N/mm^2';'k_c*f_c_0_d' 'N/mm^2'};
%columnsvar_values = [f_c_0_d,b_co,h_co,L_co,A_co,V_co,I_y_co,I_z_co,i_y_co,i_z_co,g_0k_co,g_Ak_co,N_k_co_s1,N_k_co_s2,N_d_co_s1,N_d_co_s2,bucklingfactor_y,bucklingfactor_z,L_k_y_co,L_k_z_co,lambda_y,lambda_z,lambda,lambda_rel,betha_c,k,k_c,sigma_c_0_d,sigma_c_0_k,f_c_0_d,k_c*f_c_0_d];

%disp(char(' '))
%disp(char(' '))
%disp('Columns verification')
%disp(['Material:',' ',char(sortofmaterialcolumn)])
%disp(['Strength Class:',' ',char(strengthclasscolumn)])
%disp(char({'';'';'Start Values Columns';''}))
```

```
%for i = 1:size(columnsvar_names,1)
   %disp([char(columnsvar_names(i,1)),' = ', num2str(columnsvar_values(i)),'  ',
char(columnsvar_names(i,2))])
%end
%disp(char(' '))
%disp(char(buckling))
%disp(char(' '))
%disp([char('b_co'),' = ', num2str(b_co),'  ', char('m')])
%disp([char('h_co'),' = ', num2str(h_co),'  ', char('m')])
%disp(char(' '))

while sigma_c_0_d > k_c*f_c_0_d
        h_co = h_co + 0.05;
        L_co=H_st+h_be/2+0.3;
        A_co=b_co*h_co;
        V_co=A_co*L_co;
        I_y_co=b_co*h_co^3/12;
        I_z_co=h_co*b_co^3/12;
        i_y_co=sqrt(I_y_co/A_co);
        i_z_co=sqrt(I_z_co/A_co);

        %Loads (own Weight could be neglected for static)
        g_0k_co=5;
        g_Ak_co=1;%Assumption 100kg/m^2
        N_k_co_s1=(n_st_o-1)*((s1+s2)*A_be*g_0k_be+s1*s2*g_Ak_be+...
            V_co*g_0k_co+(s1+s2)*L_co*g_Ak_co)+...%Assumptions for an intern column with intern
Walls over it.
            s1*s2*(q_Nk_S+(n_st_o-1)*(q_Nk_resid*usage_resid+q_Nk_office*usage_office));
        N_k_co_s2=N_k_co_s1;
        N_d_co_s1=1.35*((n_st_o-1)*((s1+s2)*A_be*g_0k_be+s1*s2*g_Ak_be+...
            V_co*g_0k_co+(s1+s2)*L_co*g_Ak_co))+...%Assumptions for an intern column with intern
Walls over it.
            1.5*(s1*s2*(q_Nk_S+(n_st_o-1)*(q_Nk_resid*usage_resid+q_Nk_office*usage_office)));
        N_d_co_s2=N_d_co_s1;

        %Compression & Buckling (SIA 265,(10)/(29))
        bucklingfactor_y=1.0;%Pin supported
        bucklingfactor_z=0.0;%Buckling restrained by OSB pannels along the wall
        L_k_y_co=bucklingfactor_y*L_co;
        L_k_z_co=bucklingfactor_z*L_co;
        lambda_y=L_k_y_co/i_y_co;
        lambda_z=L_k_z_co/i_z_co;
        lambda=max(lambda_y,lambda_z);
        if strcmp(sortofmaterialcolumn,'solid')
            lambda_rel=lambda/(18*pi);
            betha_c=0.2;
        else
            lambda_rel=lambda/(20*pi);
            betha_c=0.1;
        end
        if lambda_rel < 0.3%control of minimal Slenderness
            buckling='lambda_rel < 0.3 -> no buckling';
        else
            buckling='buckling to proof ';
        end
        k=0.5*(1+betha_c*(lambda_rel-0.3)+lambda_rel^2);
```

```
        k_c=1/(k+sqrt(k^2-lambda_rel^2));
        if k_c > 1
            disp(char(''))
            disp(char('k_c > 1 -> no buckling'));
            break;
        end
        sigma_c_0_k=N_k_co_s1/A_co/1000;%brought to N/mm^2
        sigma_c_0_d=N_d_co_s1/A_co/1000;%brought to N/mm^2
end

%Display Variables
%columnsvar_names = {'f_c_0_d' 'N/mm^2';'b_co' 'm';'h_co' 'm';'L_co' 'm';'A_co' 'm^2';'V_co'
'm^3';'I_y_co' 'm^4';'I_z_co' 'm^4';'i_y_co' 'm';'i_z_co' 'm';'g_0k_co' 'kN/m^3';'g_Ak_co'
'kN/m^2';'N_k_co_s1' 'kN';'N_k_co_s2' 'kN';'N_d_co_s1' 'kN';'N_d_co_s2' 'kN';'bucklingfactor_y' '-
';'bucklingfactor_z' '-';'L_k_y_co' 'm';'L_k_z_co' 'm';'lambda_y' '-';'lambda_z' '-';'lambda' '-
';'lambda_rel' '-';'betha_c=0.2' '-';'k' '-';'k_c' '-';'sigma_c_0_d' 'N/mm^2   (must be <
f_c_0_d)';'sigma_c_0_k' 'N/mm^2   (must be < k_c*f_c_0_d)';'f_c_0_d' 'N/mm^2';'k_c*f_c_0_d'
'N/mm^2'};
%columnsvar_values =
[f_c_0_d,b_co,h_co,L_co,A_co,V_co,I_y_co,I_z_co,i_y_co,i_z_co,g_0k_co,g_Ak_co,N_k_co_s1,N
_k_co_s2,N_d_co_s1,N_d_co_s2,bucklingfactor_y,bucklingfactor_z,L_k_y_co,L_k_z_co,lambda_y
,lambda_z,lambda,lambda_rel,betha_c,k,k_c,sigma_c_0_d,sigma_c_0_k,f_c_0_d,k_c*f_c_0_d];

%disp(char({'';'';'Final Values Columns';''}))

%for i = 1:size(columnsvar_names,1)
    %disp([char(columnsvar_names(i,1)),' = ', num2str(columnsvar_values(i)),'  ',
char(columnsvar_names(i,2))])
%end
%disp(char(' '))
%disp(char(buckling))
%disp(char(' '))
%disp([char('b_co'),' = ', num2str(b_co),'  ', char('m')])
%disp([char('h_co'),' = ', num2str(h_co),'  ', char('m')])
%disp(char(' '))

%Volumes
%Extern walls and structural intern walls too.
%Intern walls assumed to be spaced with s1
%1 Story
nrcolumns=(min(L,B)/s1+1)*(max(L,B)/s2+1)+5/s2;%3 main walls and one 5m-long intern wall
%n Stories
Nrcolumns=nrcolumns*n_st_o;

volumecolumns(n_st_o)=b_co*h_co*H_st*Nrcolumns;%geometrical height column is intern storey
height
volumesupports(n_st_o)=2*0.06*h_co*(max(B,L)*(min(B,L)/s1+1)+min(B,L)/2)*n_st_o;%2 rows of
supports over whole lenght of 3 main walls and of half of the width intern wall
volumevertical(n_st_o)=volumecolumns(n_st_o)+volumesupports(n_st_o);
volumepannels(n_st_o)=2*0.024*(H_st+2*0.06)*(max(B,L)*(min(B,L)/s1+1)+min(B,L)/2)*n_st_o;%2
shear pannels of thickness 24mm on whole lenght of walls

%Display Volumes
%volumecolumnsvar_names = {'nrcolumns' 'each story';'Nr. columns' 'over whole building';'Total
Volume columns' 'm^3'};
%volumecolumnsvar_values = [nrcolumns,Nrcolumns,volumecolumns(n_st_o)];
```

```
%disp(char({'';'Total Nr. and Volume of Columns';''}))

%for i = 1:size(volumecolumnsvar_names,1)
   %disp([char(volumecolumnsvar_names(i,1)),' = ', num2str(volumecolumnsvar_values(i)),'  ',
char(volumecolumnsvar_names(i,2))])
%end
%disp(char(' '))
%% Foundation
%Material Properties
%Ground
sigma_bd=300;%in kN/m^2;
%Concrete
sortofmaterialfoundation='concrete';
strengthclassfoundation='C25/30';
if strcmp(strengthclassfoundation,'C25/30')
   f_cd=16.5;%connect this value to the material with if function.
else%C30/37
   f_cd=20;
end

%Profile
%b_co and h_co of cross-section known
P_found=0.03;%armature content
t_plate=0.1;%10cm of concrete for closing of flat foundation for isolation
t_found=t_plate;%total thickness under columns
s_found=h_co+2*t_found;%Force propagation 1:1
f_found=s_found;%width of local foundation, f_found > s_found

%Compression
%Ground
sigma_b_d=N_d_co_s1/(f_found*1);%in kN/m^2, ribbon foundation for 1m length
%sigma_bd ? sigma_b_d

%Concrete
%sigma_c_0_d=N_d_co_s1/A_co/1000; ? sigma_c_Rd
%SIA262,4.2.1.10 with 1:2 compression propagation resistence
k_c_found(n_st_o)=1/sqrt((h_co*b_co)/((h_co+t_found)*(b_co+t_found)));
if k_c_found(n_st_o) > 3
   disp(char(''))
   disp(char('k_c_found > 3 (SIA 262,(32))'));%if k_c_found > 3, the compression of the concrete is
too high -> either bigger foundation or more columns, for less compression
   break;
end
sigma_c_Rd=k_c_found(n_st_o)*f_cd;%k_c_found contains distribution of force

%Display Variables
% foundationvar_names = {'sigma_bd' 'kN/m^2';'f_cd' 'N/mm^2';'b_co' 'm';'h_co' 'm';'A_co'
'm^2';'t_plate' 'm';'t_found' 'm';'s_found' 'm';'f_found' 'm';'N_d_co_s1' 'kN';'sigma_b_d' 'kN/m`   (must
be < sigma_bd)';'sigma_bd' 'kN/m`';'k_c_found' '-';'sigma_c_0_d' 'N/mm^2   (must be <
sigma_c_Rd)';'sigma_c_Rd' 'N/mm^2'};
% foundationvar_values =
[sigma_bd,f_cd,b_co,h_co,A_co,t_plate,t_found,s_found,f_found,N_d_co_s1,sigma_b_d,sigma_bd
,k_c_found,sigma_c_0_d,sigma_c_Rd];
%
% disp(char(' '))
```

```
% disp(char(' '))
% disp('Foundation verification')
% disp(['Material:',' ','Concrete'])
% disp(['Strength Class:',' ',char(strengthclassfoundation)])
% disp(char({'';'';'Start Values Foundation';''}))
%
% for i = 1:size(foundationvar_names,1)
%     disp([char(foundationvar_names(i,1)),' = ', num2str(foundationvar_values(i)),'  ',
char(foundationvar_names(i,2))])
% end
% disp(char(' '))
% disp([char('h_co'),' = ', num2str(h_co),'  ', char('m')])
% disp([char('t_plate'),' = ', num2str(t_plate),'  ', char('m')])
% disp([char('t_found'),' = ', num2str(t_found),'  ', char('m')])
% disp([char('s_found'),' = ', num2str(s_found),'  ', char('m')])
% disp([char('f_found'),' = ', num2str(f_found),'  ', char('m')])
% disp(char(' '))

while sigma_c_0_d > sigma_c_Rd || sigma_b_d > sigma_bd
   %Profile
   %b_co and h_co of cross-section known
   t_found=t_found + 0.01;%total thickness under columns
   s_found=h_co+2*t_found;%Force propagation 1:1
   f_found=s_found;%width of local foundation, f_found > s_found

   %Compression
   %Ground
   sigma_b_d=N_d_co_s1/(f_found*1);%in kN/m^2, ribbon foundation for 1m length
   %sigma_bd ? sigma_b_d

   %Concrete
   %sigma_c_0_d=N_d_co_s1/A_co/1000; ? sigma_c_Rd
   %SIA262,4.2.1.10 with 1:2 compression propagation resistence
   k_c_found(n_st_o)=1/sqrt((h_co*b_co)/((h_co+t_found)*(b_co+t_found)));
   if k_c_found(n_st_o) > 3
      disp(char(''))
      disp(char('k_c_found > 3 (SIA 262,(32))'));%if k_c_found > 3, the compression of the concrete
is too high -> either bigger foundation or more columns, for less compression
      break;
   end
   sigma_c_Rd=k_c_found(n_st_o)*f_cd;%k_c_found contains distribution of force
end

%Display Variables
% foundationvar_names = {'sigma_bd' 'kN/m^2';'f_cd' 'N/mm^2';'b_co' 'm';'h_co' 'm';'A_co'
'm^2';'t_plate' 'm';'t_found' 'm';'s_found' 'm';'f_found' 'm';'N_d_co_s1' 'kN';'sigma_b_d' 'kN/m`   (must
be < sigma_bd)';'sigma_bd' 'kN/m`';'k_c_found' '-';'sigma_c_0_d' 'N/mm^2   (must be <
sigma_c_Rd)';'sigma_c_Rd' 'N/mm^2'};
% foundationvar_values =
[sigma_bd,f_cd,b_co,h_co,A_co,t_plate,t_found,s_found,f_found,N_d_co_s1,sigma_b_d,sigma_bd
,k_c_found,sigma_c_0_d,sigma_c_Rd];
%
% disp(char({'';'';'Final Values Foundation';''}))
%
% for i = 1:size(foundationvar_names,1)
```

```
%     disp([char(foundationvar_names(i,1)),' = ', num2str(foundationvar_values(i)),'  ',
char(foundationvar_names(i,2))])
% end
% disp(char(' '))
% disp([char('h_co'),' = ', num2str(h_co),'  ', char('m')])
% disp([char('t_plate'),' = ', num2str(t_plate),'  ', char('m')])
% disp([char('t_found'),' = ', num2str(t_found),'  ', char('m')])
% disp([char('s_found'),' = ', num2str(s_found),'  ', char('m')])
% disp([char('f_found'),' = ', num2str(f_found),'  ', char('m')])
% disp(char(' '))


%Volumes
volumeplate=t_plate*(B+2*(s_found/2-h_co/2))*(L+2*(s_found/2-h_co/2));
volumeribbon=(t_found-t_plate)*f_found*(2*min(B,L)+max(B,L)*(min(B,L)/s1+1));
volumefoundation_concrete(n_st_o)=volumeplate + volumeribbon;
volumefoundation_armature(n_st_o)=volumefoundation_concrete(n_st_o)*P_found;

% Display Volumes
% disp([char('Volume plate'),' = ', num2str(volumeplate),'  ', char('m^3')])
% disp([char('Volume ribbon'),' = ', num2str(volumeribbon),'  ', char('m^3')])
% disp([char('Tot. Volume foundation'),' = ', num2str(volumefoundation_concrete(n_st_o)),'  ',
char('m^3')])
% disp([char('Tot. Volume armature'),' = ', num2str(volumefoundation_armature(n_st_o)),'  ',
char('m^3')])
% disp(char(' '))
%% Summary
disp(char(' '))
disp(char(' '))
disp(char('Summary of Values'))
disp(char(' '))
disp(char('SCHEMA 2 : Platform timber frame prefab load-bearing walls and beams'))
disp([char('Nr. stories'),' = ', num2str(n_st_o)])
disp(char(' '))
disp(char('Beams'))
disp([char('Material'),': ', num2str(sortofmaterialbeam),', ', num2str(strengthclassbeam)])
beams_names = {'b_be' 'm';'h_be' 'm';'Total Volume' 'm^3'};
beams_values = [b_be,h_be,volumehorizontal(n_st_o)];
% for i = 1:size(beams_values,2)
%     disp([char(beams_names(i,1)),' = ', num2str(beams_values(i)),'  ', char(beams_names(i,2))])
% end
for i = 1:size(beams_values,2)%only values output
   disp(num2str(beams_values(i)))
end
disp(char(' '))
disp(char('Columns'))
disp([char('Material'),': ', num2str(sortofmaterialcolumn),', ', num2str(strengthclasscolumn)])
columns_names = {'b_co' 'm';'h_co' 'm';'Total Volume' 'm^3'};
columns_values = [b_co,h_co,volumevertical(n_st_o)];
% for i = 1:size(columns_values,2)
%     disp([char(columns_names(i,1)),' = ', num2str(columns_values(i)),'  ',
char(columns_names(i,2))])
% end
for i = 1:size(columns_values,2)%only values output
   disp(num2str(columns_values(i)))
end
```

```
disp([char('Material'),': ', 'OSB'])
disp(num2str(volumepannels(n_st_o)))
disp(char(' '))
disp(char('Foundation'))
disp([char('Material'),': ', 'Concrete' ,', ', num2str(strengthclassfoundation)])
foundation_names = {'t_plate' 'm';'t_found' 'm';'s_found' 'm';'f_found' 'm'; 'Armature content' '-';
'Volume plate' 'm^3';'Volume ribbon foundation' 'm^3';'Tot. Volume foundation' 'm^3';'Tot. Volume
armature' 'm^3'};
foundation_values =
[t_plate,t_found,s_found,f_found,P_found,volumeplate,volumeribbon,volumefoundation_concrete(n
_st_o),volumefoundation_armature(n_st_o)];
% for i = 1:size(foundation_values,2)
%     disp([char(foundation_names(i,1)),' = ', num2str(foundation_values(i)),'  ',
char(foundation_names(i,2))])
% end
for i = 1:size(foundation_values,2)%only values output
   disp(num2str(foundation_values(i)))
end
disp(char(' '))
disp(char(' '))
disp(char(' '))
disp(char(' '))
disp(char('TOTAL Materials'))
volume_solidwood_tot(n_st_o)=volumehorizontal(n_st_o)+volumevertical(n_st_o);
TOTAL_names = {'Tot. Volume Solid Wood' 'm^3';'Tot. Volume OSB' 'm^3';'Tot. Volume Concrete
C25/30' 'm^3';'Tot. Volume Armature' 'm^3'};
TOTAL_values =
[volume_solidwood_tot(n_st_o),volumepannels(n_st_o),volumefoundation_concrete(n_st_o),volum
efoundation_armature(n_st_o)];
% for i = 1:size(TOTAL_values,2)
%     disp([char(TOTAL_names(i,1)),' = ', num2str(TOTAL_values(i)),'  ', char(TOTAL_names(i,2))])
% end
for i = 1:size(TOTAL_values,2)%only values output
   disp(num2str(TOTAL_values(i)))
end
disp(char(' '))
disp(char(' '))
usageratio_solidwood_tot(n_st_o)=volume_solidwood_tot(n_st_o)/(B*L*n_st_o);
usageratio_pannels(n_st_o)=volumepannels(n_st_o)/(B*L*n_st_o);
usageratio_foundation_concrete(n_st_o)=volumefoundation_concrete(n_st_o)/(B*L*n_st_o);
usageratio_foundation_armature(n_st_o)=volumefoundation_armature(n_st_o)/(B*L*n_st_o);
usage_names = {'Usage ratio Solid Wood' 'm^3/m^2 RES';'Usage ratio OSB' 'm^3/m^2
RES';'Usage ratio Concrete C25/30' 'm^3/m^2 RES';'Usage Armature' 'm^3/m^2 RES'};
usage_values =
[usageratio_solidwood_tot(n_st_o),usageratio_pannels(n_st_o),usageratio_foundation_concrete(n
_st_o),usageratio_foundation_armature(n_st_o)];
% for i = 1:size(usage_values,2)
%     disp([char(usage_names(i,1)),' = ', num2str(usage_values(i)),'  ', char(usage_names(i,2))])
% end
for i = 1:size(usage_values,2)%only values output
   disp(num2str(usage_values(i)))
end

usageratio_impact_solid(n_st_o)=-46.8*usageratio_solidwood_tot(n_st_o);
usageratio_impact_OSB(n_st_o)=262.67*usageratio_pannels(n_st_o);
usageratio_impact_concrete(n_st_o)=227.70*usageratio_foundation_concrete(n_st_o);
```

```
usageratio_impact_steel(n_st_o)=5353.7*usageratio_foundation_armature(n_st_o);
usageratio_impact_tot(n_st_o)=usageratio_impact_solid(n_st_o)+usageratio_impact_OSB(n_st_o)
+usageratio_impact_concrete(n_st_o)+usageratio_impact_steel(n_st_o);
end
figure(21);
plot(1:maxstories,volumebeams_tot)
hold on;
plot(1:maxstories,volumepavement)
plot(1:maxstories,volumehorizontal)
plot(1:maxstories,volumecolumns)
plot(1:maxstories,volumesupports)
plot(1:maxstories,volumevertical)
plot(1:maxstories,volumepannels)
plot(1:maxstories,volume_solidwood_tot)
plot(1:maxstories,volumefoundation_concrete)
plot(1:maxstories,volumefoundation_armature)
legend('beams','pavement','Solid Wood horizontal','columns','wall supports','Solid Wood
vertical','OSB pannels','TOTAL Solid Wood','foundation Concrete C25/30','foundation Armature')
xlabel('number of stories')
ylabel('volume [m^3]')
xlim([1 8])
ylim([-40 140])
annotation('doublearrow',[0.13,0.31],[0.24,0.24])
annotation('textbox',[0.17,0.24,0.1,0],'string','SFH & TH')
annotation('doublearrow',[0.13,0.545],[0.2,0.2])
annotation('textbox',[0.29,0.2,0.05,0],'string','MFH')
annotation('doublearrow',[0.13,0.905],[0.16,0.16])
annotation('textbox',[0.47,0.16,0.04,0],'string','AB')
hold off;
saveas(gcf,'images/Matlab plots/21_ tim_totvolumes_all','epsc')
figure(22);
plot(1:maxstories,volumepannels)
hold on;
plot(1:maxstories,volume_solidwood_tot)
plot(1:maxstories,volumefoundation_concrete)
plot(1:maxstories,volumefoundation_armature)
legend('OSB pannels','TOTAL Solid Wood','foundation Concrete C25/30','foundation Armature')
xlabel('number of stories')
ylabel('volume [m^3]')
xlim([1 8])
ylim([-40 140])
annotation('doublearrow',[0.13,0.31],[0.24,0.24])
annotation('textbox',[0.17,0.24,0.1,0],'string','SFH & TH')
annotation('doublearrow',[0.13,0.545],[0.2,0.2])
annotation('textbox',[0.29,0.2,0.05,0],'string','MFH')
annotation('doublearrow',[0.13,0.905],[0.16,0.16])
annotation('textbox',[0.47,0.16,0.04,0],'string','AB')
hold off;
saveas(gcf,'images/Matlab plots/22_tim_totvolumes_summary','epsc')
figure(23);
plot(1:maxstories,usageratio_solidwood_tot)
hold on;
plot(1:maxstories,usageratio_pannels)
plot(1:maxstories,usageratio_foundation_concrete)
plot(1:maxstories,usageratio_foundation_armature)
```

```
legend('Total ratio Solid Wood','Total ratio OSB pannels','ratio foundation Concrete C25/30','ratio foundation Armature')
xlabel('number of stories')
ylabel('ratio [m^3/m^2 RES]')
xlim([1 8])
ylim([-0.04 .16])
annotation('doublearrow',[0.13,0.31],[0.24,0.24])
annotation('textbox',[0.17,0.24,0.1,0],'string','SFH & TH')
annotation('doublearrow',[0.13,0.545],[0.2,0.2])
annotation('textbox',[0.29,0.2,0.05,0],'string','MFH')
annotation('doublearrow',[0.13,0.905],[0.16,0.16])
annotation('textbox',[0.47,0.16,0.04,0],'string','AB')
hold off;
saveas(gcf,'images/Matlab plots/23_ tim _RES','epsc')
figure(24);
plot(1:maxstories,usageratio_impact_solid)
hold on;
plot(1:maxstories,usageratio_impact_OSB)
plot(1:maxstories,usageratio_impact_concrete)
plot(1:maxstories,usageratio_impact_steel)
plot(1:maxstories,usageratio_impact_tot)
legend('ratio solid impact','ratio OSB impacts','ratio concrete impact','ratio steel impact','ratio total building impact')
xlabel('number of stories')
ylabel('ratio impact [kg CO_2 / m^2 RES]')
xlim([1 8])
ylim([-30 50])
annotation('doublearrow',[0.13,0.31],[0.24,0.24])
annotation('textbox',[0.17,0.24,0.1,0],'string','SFH & TH')
annotation('doublearrow',[0.13,0.545],[0.2,0.2])
annotation('textbox',[0.29,0.2,0.05,0],'string','MFH')
annotation('doublearrow',[0.13,0.905],[0.16,0.16])
annotation('textbox',[0.47,0.16,0.04,0],'string','AB')
hold off;
saveas(gcf,'images/Matlab plots/24_ tim_CO2impact','epsc')
%% SCHEME 3 : Cross-laminated bamboo (CLB) load-bearing walls and floor panels
maxstories = 8;
k_c_found = zeros(maxstories,1);
volumeslabs = zeros(maxstories,1);
volumewalls = zeros(maxstories,1);
volumefoundation_concrete = zeros(maxstories,1);
volumefoundation_armature = zeros(maxstories,1);
volume_Biobased_tot = zeros(maxstories,1);
usageratio_Biobased_tot = zeros(maxstories,1);
usageratio_foundation_concrete = zeros(maxstories,1);
usageratio_foundation_armature = zeros(maxstories,1);
usageratio_impact_gxlam = zeros(maxstories,1);
usageratio_impact_concrete = zeros(maxstories,1);
usageratio_impact_steel = zeros(maxstories,1);
usageratio_impact_tot = zeros(maxstories,1);
for n_st_o=1:maxstories
%% Building Geometry
H_st=2.7;%Intern Height of story
L=10;
B=10;
H=n_st_o*(H_st+0.5);%30cm of suspended ceiling and 40cm/2 of Floor Beams height
```

```
s1=5;% s1 => s2 , as the static verifications are apported on the length of s1
s2=5;%spacing of intern walls
usage_resid=0;
usage_office=1;

%Display Variables
geometryvar_names = {'n_st_o' 'stories';
    'H_st' 'm';
    'L' 'm';
    'B' 'm';
    'H' 'm';
    's1' 'm';
    's2' 'm';
    'usage_resid' '(1=100%)';
    'usage_office' '(1=100%)'};
geometryvar_values = [n_st_o,H_st,L,B,H,s1,s2,...
    usage_resid,usage_office];
disp(char(' '))
disp(char(' '))
disp(char(' '))
disp(char({'Building Geometry';''}))

for i = 1:size(geometryvar_names,1)
    disp([char(geometryvar_names(i,1)),' = ', num2str(geometryvar_values(i)),'  ',
char(geometryvar_names(i,2))])
end
%% Slab
%Material Properties
sortofmaterialslab='CLB';%Cross-laminated Bamboo or Timber or solid wood
strengthclassslab='C24';%important only for timber
if strcmp(sortofmaterialslab,'CLB')
    f_m_k=80;
    f_m_d=f_m_k/1.5;%=53.33
    f_v_k=4;
    f_v_d=f_v_k/1.5;%=2.33
elseif strcmp(sortofmaterialslab,'CLT')%CLT C24
    f_m_k=24;
    f_m_d=f_m_k/1.5;%=16,reduced by k_mod=1, humidity class 1
    f_v_k=1.8;
    f_v_d=f_v_k/1.5;%=1.2, k_mod=1, humidity class 1
else%Solid C24
    f_m_d=14;%=f_m_k/1.7, because of natural material and not engineered
    f_v_d=1.5;
end
E_0_m=11000;%approximately equal for all materials
G_R=50;

%Bending Resistence verification
%Profile
b=1;%for 1m plate
d_long=0.04;
d_trans=0.02;
h_sl=3*d_long+2*d_trans;%calculation for a 5-layer CLT plate
A = zeros(3,1);
I = zeros(3,1);
a = zeros(3,1);
```

```
gamma = zeros(3,1);
I_steiner = zeros(3,1);
for i=1:3
   A(i)=b*d_long;
   I(i)=b*d_long^3/12;
   a(i)=d_long+d_trans;
   gamma(i)=1/(1+(pi^2*E_0_m*A(i))/(G_R*b/d_long*s1^2));
   I_steiner(i)=A(i)*a(i)^2*gamma(i);
end
A(2)=b*d_long;
I(2)=b*d_long^3/12;
a(2)=0;
gamma(2)=1;
I_steiner(2)=0;
A_tot=sum(A(1:3))+2*b*d_trans;
I_tot_eff=sum(I(1:3)+I_steiner(1:3));
W_tot_eff=I_tot_eff/(h_sl/2);
S_eff=b*(gamma(3)*d_long*a(3)+d_long/2*d_long/4);

%Loads
g_0k_sl=5;
g_Ak_sl=1;%Assumption of 100kg/m^2
q_Nk_S=2.5;
q_Nk_resid=2;
q_Nk_office=3;
q_k_sl_s1=A_tot*g_0k_sl+1*(g_Ak_sl+max(q_Nk_S,q_Nk_resid*usage_resid+q_Nk_office*usage_
office));
q_d_sl_s1=1.35*(A_tot*g_0k_sl+1*g_Ak_sl)+1.5*1*max(q_Nk_S,q_Nk_resid*usage_resid+q_Nk_o
ffice*usage_office);
m_d_sl_s1=q_d_sl_s1*s1^2/8;
v_d_sl_s1=q_d_sl_s1*s1/2;

%Bending resistence (SIA 265,(14))
sigma_m_d_sl_s1=m_d_sl_s1/W_tot_eff/1000;%brought to N/mm^2
exploit_bending=sigma_m_d_sl_s1/f_m_d;

%Shear resistence
tau_d_sl_s1=v_d_sl_s1*S_eff/(I_tot_eff*b)/1000;%brought to N/mm^2
exploit_shear=tau_d_sl_s1/f_v_d;

%Deflection verification
%Material Properties
phi=0.6;%humidity class 1
E_0_phi=E_0_m/(1+phi);

%Loads (SIA 260,A,tab.2)
psi_1=0.5;%frequent
psi_2=0.3;%quasi-permanent
%rare
q_k_sl_s1_rare=A_tot*g_0k_sl+1*(g_Ak_sl+...
   max(q_Nk_S,q_Nk_resid*usage_resid+q_Nk_office*usage_office));%only one payload acting
%frequent
q_k_sl_s1_fr=A_tot*g_0k_sl+1*(g_Ak_sl+...
   psi_1*max(q_Nk_S,q_Nk_resid*usage_resid+q_Nk_office*usage_office));%only one payload
acting
%quasi-permanent
```

```
q_k_sl_s1_qp=A_tot*g_0k_sl+1*(g_Ak_sl+...
    psi_2*max(q_Nk_S,q_Nk_resid*usage_resid+q_Nk_office*usage_office));%only one payload
acting

%Deflections (SIA 260,A,tab.3)
%rare
w_sl_s1_rare=1/192*q_k_sl_s1_rare*s1^4/(E_0_phi*I_tot_eff);%2-field carrier
w_sl_s1_lim_rare=s1/0.500;
exploit_defl_rare=w_sl_s1_rare/w_sl_s1_lim_rare;
%frequent
w_sl_s1_fr=1/192*q_k_sl_s1_fr*s1^4/(E_0_phi*I_tot_eff);%2-field carrier
w_sl_s1_lim_fr=s1/0.350;
exploit_defl_fr=w_sl_s1_fr/w_sl_s1_lim_fr;
%quasi-permanent
w_sl_s1_qp=1/192*q_k_sl_s1_qp*s1^4/(E_0_phi*I_tot_eff);%2-field carrier
w_sl_s1_lim_qp=s1/0.300;
exploit_defl_qp=w_sl_s1_qp/w_sl_s1_lim_qp;

%Exploit of geometry
maxexploitslabs_value=max([exploit_bending,exploit_shear,exploit_defl_rare,exploit_defl_fr,exploit
_defl_qp]);
if maxexploitslabs_value == exploit_bending
    maxexploitslabs_name = 'Bending';
elseif maxexploitslabs_value == exploit_shear
    maxexploitslabs_name = 'Shear';
elseif maxexploitslabs_value == exploit_defl_rare
    maxexploitslabs_name = 'Deflection rare loading';
elseif maxexploitslabs_value == exploit_defl_fr
    maxexploitslabs_name = 'Deflection frequen loading';
else
    maxexploitslabs_name = 'Deflection quasi-permanent loading';
end

%Volumes
volumeslabs(n_st_o)=B*L*h_sl*n_st_o;
%% Walls
%Material Properties
sortofmaterialwall='CLB';%Cross-laminated Bamboo or Timber or solid wood
strengthclasswall='C24';%important only for timber
if strcmp(sortofmaterialwall,'CLB')
    f_c_0_d=63;
elseif strcmp(sortofmaterialwall,'CLT')%CLT C24
    f_c_0_d=14.5;%k_mod=1, humidity class 1
else%Solid C24
    f_c_0_d=12;
end

%Profile
b=1;%for 1m plate
d_long=0.02;
d_trans=0.02;
h_co=3*d_long+2*d_trans;%calculation for a 5-layer CLT plate
L_co=2.8;%static & geometrical length of wall
A = zeros(3,1);
I = zeros(3,1);
a = zeros(3,1);
```

```
gamma = zeros(3,1);
I_steiner = zeros(3,1);
for i=1:3
   A(i)=b*d_long;
   I(i)=b*d_long^3/12;
   a(i)=d_long+d_trans;
   gamma(i)=1/(1+(pi^2*E_0_m*A(i))/(G_R*b/d_long*L_co^2));
   I_steiner(i)=A(i)*a(i)^2*gamma(i);
end
A(2)=b*d_long;
I(2)=b*d_long^3/12;
a(2)=0;
gamma(2)=1;
I_steiner(2)=0;
A_tot=sum(A(1:3))+2*b*d_trans;
A_tot_eff=sum(A(1:3));%only long. cross-section
I_tot_eff=sum(I(1:3)+I_steiner(1:3));
i_y_co=sqrt(I_tot_eff/A_tot_eff);

%Loads (own Weight could be neglected for static)
g_0k_co=2.35;%kN/m
n_k_co_s1=(n_st_o-1)*(s1*(h_sl*g_0k_sl+g_Ak_sl))+g_0k_co+...%self-load of wall acting on it
   s1*(q_Nk_S+(n_st_o-1)*(q_Nk_resid*usage_resid+q_Nk_office*usage_office));
n_d_co_s1=1.35*((n_st_o-1)*(s1*(h_sl*g_0k_sl+g_Ak_sl))+g_0k_co)+...
   1.5*(s1*(q_Nk_S+(n_st_o-1)*(q_Nk_resid*usage_resid+q_Nk_office*usage_office)));

%Compression & Buckling (SIA 265,(10)/(29))
bucklingfactor_y=1.0;%Pin supported
L_k_y_co=bucklingfactor_y*L_co;
lambda_y=L_k_y_co/i_y_co;
if (strcmp(sortofmaterialwall,'CLB')) || (strcmp(sortofmaterialwall,'CLT'))
   lambda_rel=lambda_y/(20*pi);
   betha_c=0.1;
else%solid
   lambda_rel=lambda_y/(18*pi);
   betha_c=0.2;
end
if lambda_rel < 0.3%control of minimal Slenderness
   buckling='lambda_rel < 0.3 -> no buckling';
else
   buckling='buckling to proof ';
end
k=0.5*(1+betha_c*(lambda_rel-0.3)+lambda_rel^2);
k_c=1/(k+sqrt(k^2-lambda_rel^2));
if k_c > 1
   disp(char(''))
   disp(char('k_c > 1 -> no buckling'));
   break;
end
sigma_c_0_k=n_k_co_s1/A_tot_eff/1000;%brought to N/mm^2
sigma_c_0_d=n_d_co_s1/A_tot_eff/1000;%brought to N/mm^2
exploit_compression=sigma_c_0_d/f_c_0_d;
exploit_buckling=sigma_c_0_d/(k_c*f_c_0_d);

%Exploit of geometry
```

```
maxexploitwalls_value=max([exploit_compression,exploit_buckling]);%this part has no sense, of
course buckling is allways determinant
if maxexploitwalls_value == exploit_compression
   maxexploitwalls_name = 'Compression';
else
   maxexploitwalls_name = 'Buckling';
end

%Volumes
volumewalls(n_st_o)=L_co*h_co*(max(B,L)*(min(B,L)/s1+1)+min(B,L)/2)*n_st_o;%3 long walls and
1 intern 5m-long wall
%% Foundation
%Material Properties
%Ground
sigma_bd=300;%in kN/m^2;
%Concrete
sortofmaterialfoundation='concrete';
strengthclassfoundation='C25/30';
if strcmp(strengthclassfoundation,'C25/30')
   f_cd=16.5;%connect this value to the material with if function.
else%C30/37
   f_cd=20;
end

%Profile
%b_co and h_co of cross-section known
P_found=0.03;%armature content
t_plate=0.1;%10cm of concrete for closing of flat foundation for isolation
t_found=t_plate;%total thickness under columns
s_found=h_co+2*t_found;%Force propagation 1:1
f_found=s_found;%width of local foundation, f_found > s_found

%Compression
%Ground
sigma_b_d=n_d_co_s1/(f_found*1);%in kN/m^2, ribbon foundation for 1m length
%sigma_bd ? sigma_b_d

%Concrete
%sigma_c_0_d=N_d_co_s1/A_co/1000; ? sigma_c_Rd
%SIA262,4.2.1.10 with 1:2 compression propagation resistence
k_c_found(n_st_o)=1/sqrt((h_co*b)/((h_co+t_found)*(b+t_found)));
if k_c_found(n_st_o) > 3
   disp(char('k_c_found > 3 (SIA 262,(32))'));%if k_c_found > 3, the compression of the concrete is
too high -> either bigger foundation or more columns, for less compression
   break;
end
sigma_c_Rd=k_c_found(n_st_o)*f_cd;%k_c_found contains distribution of force


while sigma_c_0_d > sigma_c_Rd || sigma_b_d > sigma_bd
   %Profile
   %b_co and h_co of cross-section known
   t_found=t_found + 0.01;%total thickness under columns
   s_found=h_co+2*t_found;%Force propagation 1:1
   f_found=s_found;%width of local foundation, f_found > s_found
```

```
    %Compression
    %Ground
    sigma_b_d=n_d_co_s1/(f_found*1);%in kN/m^2, ribbon foundation for 1m length
    %sigma_bd ? sigma_b_d

    %Concrete
    %sigma_c_0_d=N_d_co_s1/A_co/1000; ? sigma_c_Rd
    %SIA262,4.2.1.10 with 1:2 compression propagation resistence
    k_c_found(n_st_o)=1/sqrt((h_co*b)/((h_co+t_found)*(b+t_found)));
    if k_c_found(n_st_o) > 3
        disp(char(''))
        disp(char('k_c_found > 3 (SIA 262,(32))'));%if k_c_found > 3, the compression of the concrete
is too high -> either bigger foundation or more columns, for less compression
        break;
    end
    sigma_c_Rd=k_c_found(n_st_o)*f_cd;%k_c_found contains distribution of force
end

%Volumes
volumeplate=t_plate*(B+2*(s_found/2-h_co/2))*(L+2*(s_found/2-h_co/2));
volumeribbon=(t_found-t_plate)*f_found*(2*min(B,L)+max(B,L)*(min(B,L)/s1+1));
volumefoundation_concrete(n_st_o)=volumeplate + volumeribbon;
volumefoundation_armature(n_st_o)=volumefoundation_concrete(n_st_o)*P_found;
%% Summary
disp(char(' '))
disp(char(' '))
disp(char('Summary of Values'))
disp(char(' '))
disp(char('SCHEME 3 : Cross-laminated bamboo (CLB) load-bearing walls and floor panels'))
disp([char('Nr. stories'),' = ', num2str(n_st_o)])
disp(char(' '))
disp(char('Slabs'))
if strcmp(sortofmaterialslab,'CLB')
    disp([char('Material'),': ', num2str(sortofmaterialslab)])
else
    disp([char('Material'),': ', num2str(sortofmaterialslab),', ', num2str(strengthclassslab)])
end
slab_names = {'b' 'm';'h_sl' 'm';'Total Volume' 'm^3'};
slab_values = [b,h_sl,volumeslabs(n_st_o)];
% for i = 1:size(slab_values,2)
%     disp([char(slab_names(i,1)),' = ', num2str(slab_values(i)),'  ', char(slab_names(i,2))])
% end
for i = 1:size(slab_values,2)%only values output
    disp(num2str(slab_values(i)))
end
disp([char('Max exploit'),': ', char(maxexploitslabs_name),' = ', num2str(maxexploitslabs_value)])
disp(char(' '))
disp(char(' '))
disp(char(' '))
disp(char(' '))
disp(char(' '))
disp(char(' '))
disp(char(' '))
disp(char('Walls'))
if strcmp(sortofmaterialwall,'CLB')
    disp([char('Material'),': ', num2str(sortofmaterialwall)])
```

```
else
   disp([char('Material'),': ', num2str(sortofmaterialwall),', ', num2str(strengthclasswall)])
end
walls_names = {'b' 'm';'h_co' 'm';'Total Volume' 'm^3'};
walls_values = [b,h_co,volumewalls(n_st_o)];
% for i = 1:size(walls_values,2)
%     disp([char(walls_names(i,1)),' = ', num2str(walls_values(i)),'  ', char(walls_names(i,2))])
% end
for i = 1:size(walls_values,2)%only values output
   disp(num2str(walls_values(i)))
end
disp([char('Max exploit'),': ', char(maxexploitwalls_name),' = ', num2str(maxexploitwalls_value)])
disp(char(' '))
disp(char(' '))
disp(char('Foundation'))
disp([char('Material'),': ', 'Concrete' ,', ', num2str(strengthclassfoundation)])
foundation_names = {'t_plate' 'm';'t_found' 'm';'s_found' 'm';'f_found' 'm'; 'Armature content' '-';
'Volume plate' 'm^3';'Volume ribbon foundation' 'm^3';'Tot. Volume foundation' 'm^3';'Tot. Volume
armature' 'm^3'};
foundation_values =
[t_plate,t_found,s_found,f_found,P_found,volumeplate,volumeribbon,volumefoundation_concrete(n
_st_o),volumefoundation_armature(n_st_o)];
% for i = 1:size(foundation_values,2)
%     disp([char(foundation_names(i,1)),' = ', num2str(foundation_values(i)),'  ',
char(foundation_names(i,2))])
% end
for i = 1:size(foundation_values,2)%only values output
   disp(num2str(foundation_values(i)))
end
disp(char(' '))
disp(char(' '))
disp(char(' '))
disp(char(' '))
disp(char('TOTAL Materials'))
disp(char(' '))
volume_Biobased_tot(n_st_o)=volumeslabs(n_st_o)+volumewalls(n_st_o);
% if strcmp(sortofmaterialslab,'CLB')
%     disp([char('Tot. Volume CLB in building'),' = ', num2str(volume_Biobased_tot(n_st_o)),'  ',
char('m^3')])
% else
%     disp([char('Tot. Volume CLT in building'),' = ', num2str(volume_Biobased_tot(n_st_o)),'  ',
char('m^3')])
% end
TOTAL_names = {'Tot. Volume Biobased' 'm^3';'Tot. Volume foundation C25/30' 'm^3';'Tot.
Volume Armature' 'm^3'};
TOTAL_values =
[volume_Biobased_tot(n_st_o),volumefoundation_concrete(n_st_o),volumefoundation_armature(n
_st_o)];
% for i = 1:size(TOTAL_values,2)
%     disp([char(TOTAL_names(i,1)),' = ', num2str(TOTAL_values(i)),'  ', char(TOTAL_names(i,2))])
% end
for i = 1:size(TOTAL_values,2)%only values output
   disp(num2str(TOTAL_values(i)))
end
disp(char(' '))
disp(char(' '))
```

```
disp(char(' '))
usageratio_Biobased_tot(n_st_o)=volume_Biobased_tot(n_st_o)/(B*L*n_st_o);
% if strcmp(sortofmaterialslab,'CLB')
%     disp([char('Usage ratio CLB'),' = ', num2str(usageratio_Biobased_tot(n_st_o)),'  ',
char('m^3/m^2 RES')])
% else
%     disp([char('Usage ratio CLT'),' = ', num2str(usageratio_Biobased_tot(n_st_o)),'  ',
char('m^3/m^2 RES')])
% end
usageratio_foundation_concrete(n_st_o)=volumefoundation_concrete(n_st_o)/(B*L*n_st_o);
usageratio_foundation_armature(n_st_o)=volumefoundation_armature(n_st_o)/(B*L*n_st_o);
usage_names = {'Usage ratio Biobased' 'm^3/m^2 RES';'Usage ratio Concrete C25/30' 'm^3/m^2
RES';'Usage Armature' 'm^3/m^2 RES'};
usage_values =
[usageratio_Biobased_tot(n_st_o),usageratio_foundation_concrete(n_st_o),usageratio_foundation
_armature(n_st_o)];
% for i = 1:size(usage_values,2)
%     disp([char(usage_names(i,1)),' = ', num2str(usage_values(i)),'  ', char(usage_names(i,2))])
% end
for i = 1:size(usage_values,2)%only values output
   disp(num2str(usage_values(i)))
end

usageratio_impact_gxlam(n_st_o)=167.47*usageratio_Biobased_tot(n_st_o);%CLB)
usageratio_impact_concrete(n_st_o)=227.70*usageratio_foundation_concrete(n_st_o);%C25/30
usageratio_impact_steel(n_st_o)=5353.7*usageratio_foundation_armature(n_st_o);
usageratio_impact_tot(n_st_o)=usageratio_impact_gxlam(n_st_o)+usageratio_impact_concrete(n_
st_o)+usageratio_impact_steel(n_st_o);
end
figure(31);
plot(1:maxstories,volumeslabs)
hold on;
plot(1:maxstories,volumewalls)
plot(1:maxstories,volume_Biobased_tot)
plot(1:maxstories,volumefoundation_concrete)
plot(1:maxstories,volumefoundation_armature)
legend('slabs','walls','Biobased total','foundation Concrete C25/30','foundation Armature')
xlabel('number of stories')
ylabel('volume [m^3]')
xlim([1 8])
ylim([-100 250])
annotation('doublearrow',[0.13,0.31],[0.24,0.24])
annotation('textbox',[0.17,0.24,0.1,0],'string','SFH & TH')
annotation('doublearrow',[0.13,0.545],[0.2,0.2])
annotation('textbox',[0.29,0.2,0.05,0],'string','MFH')
annotation('doublearrow',[0.13,0.905],[0.16,0.16])
annotation('textbox',[0.47,0.16,0.04,0],'string','AB')
hold off;
saveas(gcf,'images/Matlab plots/31_herbaceous_totvolumes_all','epsc')
figure(32);
plot(1:maxstories,volume_Biobased_tot)
hold on;
plot(1:maxstories,volumefoundation_concrete)
plot(1:maxstories,volumefoundation_armature)
legend('Biobased total','foundation Concrete C25/30','foundation Armature')
xlabel('number of stories')
```

```
ylabel('volume [m^3]')
xlim([1 8])
ylim([-100 250])
annotation('doublearrow',[0.13,0.31],[0.24,0.24])
annotation('textbox',[0.17,0.24,0.1,0],'string','SFH & TH')
annotation('doublearrow',[0.13,0.545],[0.2,0.2])
annotation('textbox',[0.29,0.2,0.05,0],'string','MFH')
annotation('doublearrow',[0.13,0.905],[0.16,0.16])
annotation('textbox',[0.47,0.16,0.04,0],'string','AB')
hold off;
saveas(gcf,'images/Matlab plots/32_ herbaceous_totvolumes_summary','epsc')
figure(33);
plot(1:maxstories,usageratio_Biobased_tot)
hold on;
plot(1:maxstories,usageratio_foundation_concrete)
plot(1:maxstories,usageratio_foundation_armature)
legend('Total ratio Biobased ','ratio foundation Concrete C25/30','ratio foundation Armature')
xlabel('number of stories')
ylabel('ratio [m^3/m^2 RES]')
xlim([1 8])
ylim([-0.1 0.35])
annotation('doublearrow',[0.13,0.31],[0.24,0.24])
annotation('textbox',[0.17,0.24,0.1,0],'string','SFH & TH')
annotation('doublearrow',[0.13,0.545],[0.2,0.2])
annotation('textbox',[0.29,0.2,0.05,0],'string','MFH')
annotation('doublearrow',[0.13,0.905],[0.16,0.16])
annotation('textbox',[0.47,0.16,0.04,0],'string','AB')
hold off;
saveas(gcf,'images/Matlab plots/33_ herbaceous_RES','epsc')
figure(34);
plot(1:maxstories,usageratio_impact_gxlam)
hold on;
plot(1:maxstories,usageratio_impact_concrete)
plot(1:maxstories,usageratio_impact_steel)
plot(1:maxstories,usageratio_impact_tot)
legend('ratio CLB impact','ratio concrete impact','ratio steel impact','ratio total building impact')
xlabel('number of stories')
ylabel('ratio impact [kg CO_2 / m^2 RES]')
xlim([1 8])
ylim([-20 90])
annotation('doublearrow',[0.13,0.31],[0.24,0.24])
annotation('textbox',[0.17,0.24,0.1,0],'string','SFH & TH')
annotation('doublearrow',[0.13,0.545],[0.2,0.2])
annotation('textbox',[0.29,0.2,0.05,0],'string','MFH')
annotation('doublearrow',[0.13,0.905],[0.16,0.16])
annotation('textbox',[0.47,0.16,0.04,0],'string','AB')
hold off;
saveas(gcf,'images/Matlab plots/34_ herbaceous_CO2impact','epsc')
%% SCHEME 4 : Post and beams timber or bamboo frame with diagonal bracing and CLT or CLB
floor panels
maxstories = 14;
volumeslabs = zeros(maxstories,1);
volumebeams_tot = zeros(maxstories,1);
k_c_found = zeros(maxstories,1);
A_co = zeros(maxstories,1);
V_co = zeros(maxstories,1);
```

```
N_d_co = zeros(maxstories,1);
volumecolumns = zeros(maxstories,1);
volumediagonal = zeros(maxstories,1);
volumevertical = zeros(maxstories,1);
volumecolumns_tot = zeros(maxstories,1);
volumediagonal_tot = zeros(maxstories,1);
volumevertical_tot = zeros(maxstories,1);
volumefoundation_concrete = zeros(maxstories,1);
volumefoundation_armature = zeros(maxstories,1);
nr_micropiles_tot = zeros(maxstories,1);
volumemicropiles = zeros(maxstories,1);
volume_Biobased_beamsandverticals_tot = zeros(maxstories,1);
usageratio_slab_tot = zeros(maxstories,1);
usageratio_Biobased_beamsandverticals_tot = zeros(maxstories,1);
usageratio_concrete_tot = zeros(maxstories,1);
usageratio_armature_tot = zeros(maxstories,1);
usageratio_micropiles = zeros(maxstories,1);
usageratio_impact_slab = zeros(maxstories,1);
usageratio_impact_Biobased_beamsandverticals = zeros(maxstories,1);
usageratio_impact_concrete = zeros(maxstories,1);
usageratio_impact_steel = zeros(maxstories,1);
usageratio_impact_tot = zeros(maxstories,1);
for n_st_o=1:maxstories
%% Building Geometry
H_st=2.7;%Intern Height of story
L=10;
B=10;
H=n_st_o*(H_st+0.5);%30cm of suspended ceiling and 40cm/2 of Floor Beams height
s1=10;% s1 => s2 , as the static verifications are apported on the length of s1
s2=10;%spacing of intern walls
usage_resid=0;
usage_office=1;

%Display Variables
geometryvar_names = {'n_st_o' 'stories';
    'H_st' 'm';
    'L' 'm';
    'B' 'm';
    'H' 'm';
    's1' 'm';
    's2' 'm';
    'usage_resid' '(1=100%)';
    'usage_office' '(1=100%)'};
geometryvar_values = [n_st_o,H_st,L,B,H,s1,s2,...
    usage_resid,usage_office];
disp(char(' '))
disp(char(' '))
disp(char(' '))
disp(char({'Building Geometry';''}))

for i = 1:size(geometryvar_names,1)
    disp([char(geometryvar_names(i,1)),' = ', num2str(geometryvar_values(i)),'  ',
char(geometryvar_names(i,2))])
end
%% Slab
%Material Properties
```

```
sortofmaterialslab='CLT';%Cross-laminated Bamboo or Timber or solid wood
strengthclassslab='C24';%important only for timber
if strcmp(sortofmaterialslab,'CLB')
   f_m_k=80;
   f_m_d=f_m_k/1.5;%=53.33
   f_v_k=4;
   f_v_d=f_v_k/1.5;%=2.33
elseif strcmp(sortofmaterialslab,'CLT')%CLT C24
   f_m_k=24;
   f_m_d=f_m_k/1.5;%=12.8,reduced by k_mod=1, humidity class 1
   f_v_k=1.8;
   f_v_d=f_v_k/1.5;%=1.2, k_mod=1, humidity class 1
else%Solid C24
   f_m_d=14;%=f_m_k/1.7, because of natural material and not engineered
   f_v_d=1.5;
end
E_0_m=11000;%approximately equal for all materials
G_R=50;

%Bending Resistence verification
%Profile
b=1;%for 1m plate
d_long=0.04;
d_trans=0.02;
h_sl=3*d_long+2*d_trans;%calculation for a 5-layer CLT plate
A = zeros(1,3);
I = zeros(1,3);
a = zeros(1,3);
gamma = zeros(1,3);
I_steiner = zeros(1,3);
for i=1:3
   A(i)=b*d_long;
   I(i)=b*d_long^3/12;
   a(i)=d_long+d_trans;
   gamma(i)=1/(1+(pi^2*E_0_m*A(i))/(G_R*b/d_long*s1^2));
   I_steiner(i)=A(i)*a(i)^2*gamma(i);
end
A(2)=b*d_long;
I(2)=b*d_long^3/12;
a(2)=0;
gamma(2)=1;
I_steiner(2)=0;
A_tot=b*h_sl;
I_tot_eff=sum(I(1:3)+I_steiner(1:3));
W_tot_eff=I_tot_eff/(h_sl/2);
S_eff=b*(gamma(3)*d_long*a(3)+d_long/2*d_long/4);

%Loads
g_0k_sl=5;
g_Ak_sl=1;%Assumption of 100kg/m^2
q_Nk_S=2.5;
q_Nk_resid=2;
q_Nk_office=3;
q_k_sl_s1=A_tot*g_0k_sl+1*(g_Ak_sl+max(q_Nk_S,q_Nk_resid*usage_resid+q_Nk_office*usage_
office));
```

```
q_d_sl_s1=1.35*(A_tot*g_0k_sl+1*g_Ak_sl)+1.5*1*max(q_Nk_S,q_Nk_resid*usage_resid+q_Nk_o
ffice*usage_office);
m_d_sl_s1=q_d_sl_s1*(s1/2)^2/8;%Beams are with half of the columns spacing
v_d_sl_s1=q_d_sl_s1*s1/4;%Beams are with half of the columns spacing

%Bending resistence (SIA 265,(14))
sigma_m_d_sl_s1=m_d_sl_s1/W_tot_eff/1000;%brought to N/mm^2
exploit_bending_sl=sigma_m_d_sl_s1/f_m_d;

%Shear resistence
tau_d_sl_s1=v_d_sl_s1*S_eff/(I_tot_eff*b)/1000;%brought to N/mm^2
exploit_shear_sl=tau_d_sl_s1/f_v_d;

%Deflection verification
%Material Properties
phi=0.6;%humidity class 1
E_0_phi=E_0_m/(1+phi);

%Loads (SIA 260,A,tab.2)
psi_1=0.5;%frequent
psi_2=0.3;%quasi-permanent
%rare
q_k_sl_s1_rare=A_tot*g_0k_sl+1*(g_Ak_sl+...
   max(q_Nk_S,q_Nk_resid*usage_resid+q_Nk_office*usage_office));%only one payload acting
%frequent
q_k_sl_s1_fr=A_tot*g_0k_sl+1*(g_Ak_sl+...
   psi_1*max(q_Nk_S,q_Nk_resid*usage_resid+q_Nk_office*usage_office));%only one payload
acting
%quasi-permanent
q_k_sl_s1_qp=A_tot*g_0k_sl+1*(g_Ak_sl+...
   psi_2*max(q_Nk_S,q_Nk_resid*usage_resid+q_Nk_office*usage_office));%only one payload
acting

%Deflections (SIA 260,A,tab.3)
%rare
w_sl_s1_rare=1/192*q_k_sl_s1_rare*(s1/2)^4/(E_0_phi*I_tot_eff);%2-field carrier
w_sl_s1_lim_rare=(s1/2)/0.500;
exploit_defl_rare_sl=w_sl_s1_rare/w_sl_s1_lim_rare;
%frequent
w_sl_s1_fr=1/192*q_k_sl_s1_fr*(s1/2)^4/(E_0_phi*I_tot_eff);%2-field carrier
w_sl_s1_lim_fr=(s1/2)/0.350;
exploit_defl_fr_sl=w_sl_s1_fr/w_sl_s1_lim_fr;
%quasi-permanent
w_sl_s1_qp=1/192*q_k_sl_s1_qp*(s1/2)^4/(E_0_phi*I_tot_eff);%2-field carrier
w_sl_s1_lim_qp=(s1/2)/0.300;
exploit_defl_qp_sl=w_sl_s1_qp/w_sl_s1_lim_qp;

%Exploit of geometry
maxexploitslabs_value=max([exploit_bending_sl,exploit_shear_sl,exploit_defl_rare_sl,exploit_defl_
fr_sl,exploit_defl_qp_sl]);
if maxexploitslabs_value == exploit_bending_sl
   maxexploitslabs_name = 'Bending';
elseif maxexploitslabs_value == exploit_shear_sl
   maxexploitslabs_name = 'Shear';
elseif maxexploitslabs_value == exploit_defl_rare_sl
   maxexploitslabs_name = 'Deflection rare loading';
```

```
elseif maxexploitslabs_value == exploit_defl_fr_sl
   maxexploitslabs_name = 'Deflection frequen loading';
else
   maxexploitslabs_name = 'Deflection quasi-permanent loading';
end

%Volumes
volumeslabs(n_st_o)=B*L*h_sl*n_st_o;
%% Beams
%Material Properties
sortofmaterialbeam='glulam';%solid or glulam wood, or glued bamboo (glubam)
strengthclassbeam='C24';%important only for timber
if strcmp(sortofmaterialbeam,'glubam Bamboo')%Bamboo
   f_m_k=80;%equal to CLB
   f_m_d=f_m_k/1.5;%=53.33
elseif strcmp(sortofmaterialbeam,'glulam')%Glulam GL24h
   f_m_d=16;
else%Solid C24
   f_m_d=14;%=f_m_k/1.7, because of natural material and not engineered
end
E_0_m=11000;%approximately equal for all materials

%Bending Resistence verification
%Profile
b_be=0.3;
h_be=0.01;%ev. minimal thickness of ceiling
A_be=b_be*h_be;
W_be=b_be*h_be^2/6;

%Loads
g_0k_be=5;
g_Ak_be=1;%Assumption of 100kg/m^2
q_Nk_S=2.5;
q_Nk_resid=2;
q_Nk_office=3;
q_k_be_s2=A_be*g_0k_be+s1/2*(g_Ak_be+max(q_Nk_S,q_Nk_resid*usage_resid+q_Nk_office*u
sage_office));%Beams are with half of the columns spacing
q_k_be_s1=A_be*g_0k_be;
Q_k_be_s1=q_k_be_s2*s2/2;%two big beams as supports
q_d_be_s2=1.35*(A_be*g_0k_be+s1/2*g_Ak_be)+1.5*s1/2*max(q_Nk_S,q_Nk_resid*usage_resid
+q_Nk_office*usage_office);%Beams are with half of the columns spacing
q_d_be_s1=1.35*A_be*g_0k_be;
Q_d_be_s1=1.35*q_k_be_s2*s2/2;
m_d_be_s2=q_d_be_s2*s2^2/8;%pin supported by the perpendicular oriented two beams
m_d_be_s1=q_d_be_s1*s1^2/8+Q_d_be_s1*s1/4;%pin supported by the columns

%Bending resistence (SIA 265,(14))
sigma_m_d_be_s1=m_d_be_s1/W_be/1000;%brought to N/mm^2
sigma_m_d_be_s2=m_d_be_s2/W_be/1000;%brought to N/mm^2

%Display Variables
%beamsvar_names = {'f_m_d' 'N/mm^2';'b_be' 'm';'h_be' 'm';'A_be' 'm^2';'W_be' 'm^3';'g_0k_be'
'kN/mm^3';'g_Ak_be' 'kN/mm^2'; 'q_Nk_S' 'kN/mm^2';'q_Nk_resid' 'kN/mm^2';'q_Nk_office'
'kN/mm^2';'q_k_be_s1' 'kN/m';'q_k_be_s2' 'kN/m';'q_d_be_s1' 'kN/m';'q_d_be_s2'
'kN/m';'m_d_be_s1' 'kNm';'m_d_be_s2' 'kNm';'sigma_m_d_be_s1' 'N/mm^2   (must be < f_m_d)';
'sigma_m_d_be_s2' 'N/mm^2   (must be < f_m_d)';'f_m_d' 'N/mm^2'};
```

```
%beamsvar_values =
[f_m_d,b_be,h_be,A_be,W_be,g_0k_be,g_Ak_be,q_Nk_S,q_Nk_resid,q_Nk_office,q_k_be_s1,q_k
_be_s2,q_d_be_s1,q_d_be_s2,m_d_be_s1,m_d_be_s2,sigma_m_d_be_s1,sigma_m_d_be_s2,f_
m_d];

%disp(char(' '))
%disp(char(' '))
%disp('Beams verification')
%disp(['Material:',' ',char(sortofmaterialbeam)])
%disp(['Strength Class:',' ',char(strengthclassbeam)])
%disp(char({'';'';'Start Bending Values Beams';''}))

%for i = 1:size(beamsvar_names,1)
   %disp([char(beamsvar_names(i,1)),' = ', num2str(beamsvar_values(i)),'  ',
char(beamsvar_names(i,2))])
%end
%disp(char(' '))
%disp([char('b_be'),' = ', num2str(b_be),'  ', char('m')])
%disp([char('h_be'),' = ', num2str(h_be),'  ', char('m')])
%disp(char(' '))

while (sigma_m_d_be_s1 > f_m_d) || (sigma_m_d_be_s2 > f_m_d)
    h_be = h_be + 0.01;
    A_be=b_be*h_be;
    W_be=b_be*h_be^2/6;

    %Loads     (own Weight could be neglected for static)
    g_0k_be=5;
    g_Ak_be=1;%Assumption of 100kg/m^2
    q_Nk_S=2.5;
    q_Nk_resid=2;
    q_Nk_office=3;

q_k_be_s2=A_be*g_0k_be+s1/2*(g_Ak_be+max(q_Nk_S,q_Nk_resid*usage_resid+q_Nk_office*u
sage_office));%Beams are with half of the columns spacing
    q_k_be_s1=A_be*g_0k_be;
    Q_k_be_s1=q_k_be_s2*s2/2;%two big beams as supports

q_d_be_s2=1.35*(A_be*g_0k_be+s1/2*g_Ak_be)+1.5*s1/2*max(q_Nk_S,q_Nk_resid*usage_resid
+q_Nk_office*usage_office);%Beams are with half of the columns spacing
    q_d_be_s1=1.35*A_be*g_0k_be;
    Q_d_be_s1=1.35*q_k_be_s2*s2/2;
    m_d_be_s2=q_d_be_s2*s2^2/8;%pin supported by the perpendicular oriented two beams
    m_d_be_s1=q_d_be_s1*s1^2/8+Q_d_be_s1*s1/4;%pin supported by the columns

    %Bending resistence (SIA 265,(14))
    sigma_m_d_be_s1=m_d_be_s1/W_be/1000;%brought to N/mm^2
    sigma_m_d_be_s2=m_d_be_s2/W_be/1000;%brought to N/mm^2
end

%Display Variables
%beamsvar_names = {'f_m_d' 'N/mm^2';'b_be' 'm';'h_be' 'm';'A_be' 'm^2';'W_be' 'm^3';'g_0k_be'
'kN/mm^3';'g_Ak_be' 'kN/mm^2'; 'q_Nk_S' 'kN/mm^2';'q_Nk_resid' 'kN/mm^2';'q_Nk_office'
'kN/mm^2';'q_k_be_s1' 'kN/m';'q_k_be_s2' 'kN/m';'q_d_be_s1' 'kN/m';'q_d_be_s2'
'kN/m';'m_d_be_s1' 'kNm';'m_d_be_s2' 'kNm';'sigma_m_d_be_s1' 'N/mm^2   (must be < f_m_d)';
'sigma_m_d_be_s2' 'N/mm^2   (must be < f_m_d)';'f_m_d' 'N/mm^2'};
```

```
%beamsvar_values =
[f_m_d,b_be,h_be,A_be,W_be,g_0k_be,g_Ak_be,q_Nk_S,q_Nk_resid,q_Nk_office,q_k_be_s1,q_k
_be_s2,q_d_be_s1,q_d_be_s2,m_d_be_s1,m_d_be_s2,sigma_m_d_be_s1,sigma_m_d_be_s2,f_
m_d];

%disp(char({'';'';'Final Bending Values Beams';''}))

%for i = 1:size(beamsvar_names,1)
   %disp([char(beamsvar_names(i,1)),' = ', num2str(beamsvar_values(i)),'  ',
char(beamsvar_names(i,2))])
%end
%disp(char(' '))
%disp([char('b_be'),' = ', num2str(b_be),'  ', char('m')])
%disp([char('h_be'),' = ', num2str(h_be),'  ', char('m')])
%disp(char(' '))


%Deflection verification
%Material Properties
phi=0.6;%humidity class 1
E_0_phi=E_0_m/(1+phi);

%Profile
I_be=b_be*h_be^3/12;%in m^4

%Loads (SIA 260,A,tab.2)
psi_1=0.5;%frequent
psi_2=0.3;%quasi-permanent
%rare
q_k_be_s2_rare=A_be*g_0k_be+s1/2*(g_Ak_be+...
   max(q_Nk_S,q_Nk_resid*usage_resid+q_Nk_office*usage_office));%Beams are with half of the
columns spacing
q_k_be_s1_rare=A_be*g_0k_be;
Q_k_be_s1_rare=q_k_be_s2_rare*s2/2;%two big beams as supports

%frequent
q_k_be_s2_fr=A_be*g_0k_be+s1/2*(g_Ak_be+...
   psi_1*max(q_Nk_S,q_Nk_resid*usage_resid+q_Nk_office*usage_office));
q_k_be_s1_fr=A_be*g_0k_be;
Q_k_be_s1_fr=q_k_be_s2_fr*s2/2;%two big beams as supports

%quasi-permanent
q_k_be_s2_qp=A_be*g_0k_be+s1/2*(g_Ak_be+...
   psi_2*max(q_Nk_S,q_Nk_resid*usage_resid+q_Nk_office*usage_office));
q_k_be_s1_qp=A_be*g_0k_be;
Q_k_be_s1_qp=q_k_be_s2_qp*s2/2;%two big beams as supports


%Deflections (SIA 260,A,tab.3)
%rare
w_be_s2_rare=5/384*q_k_be_s2_rare*s2^4/(E_0_phi*I_be);
w_be_s1_rare=(5/384*q_k_be_s1_rare*s1+1/48*Q_k_be_s1_rare)*s1^3/(E_0_phi*I_be);
w_be_s2_lim_rare=s2/0.500;
w_be_s1_lim_rare=s1/0.500;
exploit_defl_s2_rare_be=w_be_s2_rare/w_be_s2_lim_rare;
exploit_defl_s1_rare_be=w_be_s1_rare/w_be_s1_lim_rare;
```

```
%frequent
w_be_s2_fr=5/384*q_k_be_s2_fr*s2^4/(E_0_phi*I_be);
w_be_s1_fr=(5/384*q_k_be_s1_fr*s1+1/48*Q_k_be_s1_fr)*s1^3/(E_0_phi*I_be);
w_be_s2_lim_fr=s2/0.350;
w_be_s1_lim_fr=s1/0.350;
exploit_defl_s2_fr_be=w_be_s2_fr/w_be_s2_lim_fr;
exploit_defl_s1_fr_be=w_be_s1_fr/w_be_s1_lim_fr;
%quasi-permanent
w_be_s2_qp=5/384*q_k_be_s2_qp*s2^4/(E_0_phi*I_be);
w_be_s1_qp=(5/384*q_k_be_s1_qp*s1+1/48*Q_k_be_s1_qp)*s1^3/(E_0_phi*I_be);
w_be_s2_lim_qp=s2/0.300;
w_be_s1_lim_qp=s1/0.300;

%Display Variables
%beamsvar_names2 = {'E_0_m' 'N/mm^2';'phi' '-';'E_0_phi' 'N/mm^2';'b_be' 'm';'h_be' 'm';'I_be'
'm^4';'g_0k_be' 'N/mm^2';'g_Ak_be' 'N/mm^2';'q_Nk_S' 'N/mm^2';'q_Nk_resid'
'N/mm^2';'q_Nk_office' 'N/mm^2';'psi_1' '-';'psi_2' '-';'q_k_be_s1_rare' 'kN/m';'q_k_be_s2_rare'
'kN/m';'q_k_be_s1_fr' 'kN/m';'q_k_be_s2_fr' 'kN/m';'q_k_be_s1_qp' 'kN/m';'q_k_be_s2_qp'
'kN/m';'w_be_s1_rare' 'mm   (must be < w_be_s1_lim_rare)';'w_be_s2_rare' 'mm   (must be <
w_be_s2_lim_rare)';'w_be_s1_lim_rare' 'mm';'w_be_s2_lim_rare' 'mm';'w_be_s1_fr' 'mm   (must be
< w_be_s1_lim_fr)';'w_be_s2_fr' 'mm   (must be < w_be_s2_lim_fr)';'w_be_s1_lim_fr'
'mm';'w_be_s2_lim_fr' 'mm';'w_be_s1_qp' 'mm   (must be < w_be_s1_lim_qp)';'w_be_s2_qp' 'mm
(must be < w_be_s1_lim_qp)';'w_be_s1_lim_qp' 'mm';'w_be_s2_lim_qp' 'mm'};
%beamsvar_values2 =
[E_0_m,phi,E_0_phi,b_be,h_be,I_be,g_0k_be,g_Ak_be,q_Nk_S,q_Nk_resid,q_Nk_office,psi_1,psi
_2,q_k_be_s1_rare,q_k_be_s2_rare,q_k_be_s1_fr,q_k_be_s2_fr,q_k_be_s1_qp,q_k_be_s2_qp,w
_be_s1_rare,w_be_s2_rare,w_be_s1_lim_rare,w_be_s2_lim_rare,w_be_s1_fr,w_be_s2_fr,w_be_s
1_lim_fr,w_be_s2_lim_fr,w_be_s1_qp,w_be_s2_qp,w_be_s1_lim_qp,w_be_s2_lim_qp];

%disp(char({'';'';'Start Deflection Values Beams';''}))

%for i = 1:size(beamsvar_names2,1)
   %disp([char(beamsvar_names2(i,1)),' = ', num2str(beamsvar_values2(i)),'  ',
char(beamsvar_names2(i,2))])
%end
%disp(char(' '))
%disp([char('b_be'),' = ', num2str(b_be),'  ', char('m')])
%disp([char('h_be'),' = ', num2str(h_be),'  ', char('m')])
%disp(char(' '))

while (w_be_s1_rare > w_be_s1_lim_rare)||(w_be_s1_fr > w_be_s1_lim_fr)||(w_be_s1_qp >
w_be_s1_lim_qp)||...
       (w_be_s2_rare > w_be_s2_lim_rare)||(w_be_s2_fr > w_be_s2_lim_fr)||(w_be_s2_qp >
w_be_s2_lim_qp)
   %Profile
   h_be = h_be + 0.01;
   A_be=b_be*h_be;
   I_be=b_be*h_be^3/12;%in m^4

   %Loads (SIA 260,A,tab.2)
   psi_1=0.5;%frequent
   psi_2=0.3;%quasi-permanent
   %rare
   q_k_be_s2_rare=A_be*g_0k_be+s1/2*(g_Ak_be+...
      max(q_Nk_S,q_Nk_resid*usage_resid+q_Nk_office*usage_office));%Beams are with half of
the columns spacing
```

```
    q_k_be_s1_rare=A_be*g_0k_be;
    Q_k_be_s1_rare=q_k_be_s2_rare*s2/2;%two big beams as supports

    %frequent
    q_k_be_s2_fr=A_be*g_0k_be+s1/2*(g_Ak_be+...
       psi_1*max(q_Nk_S,q_Nk_resid*usage_resid+q_Nk_office*usage_office));
    q_k_be_s1_fr=A_be*g_0k_be;
    Q_k_be_s1_fr=q_k_be_s2_fr*s2/2;%two big beams as supports

    %quasi-permanent
    q_k_be_s2_qp=A_be*g_0k_be+s1/2*(g_Ak_be+...
       psi_2*max(q_Nk_S,q_Nk_resid*usage_resid+q_Nk_office*usage_office));
    q_k_be_s1_qp=A_be*g_0k_be;
    Q_k_be_s1_qp=q_k_be_s2_qp*s2/2;%two big beams as supports


    %Deflections (SIA 260,A,tab.3)
    %rare
    w_be_s2_rare=5/384*q_k_be_s2_rare*s2^4/(E_0_phi*I_be);
    w_be_s1_rare=(5/384*q_k_be_s1_rare*s1+1/48*Q_k_be_s1_rare)*s1^3/(E_0_phi*I_be);
    w_be_s2_lim_rare=s2/0.500;
    w_be_s1_lim_rare=s1/0.500;
    %frequent
    w_be_s2_fr=5/384*q_k_be_s2_fr*s2^4/(E_0_phi*I_be);
    w_be_s1_fr=(5/384*q_k_be_s1_fr*s1+1/48*Q_k_be_s1_fr)*s1^3/(E_0_phi*I_be);
    w_be_s2_lim_fr=s2/0.350;
    w_be_s1_lim_fr=s1/0.350;
    %quasi-permanent
    w_be_s2_qp=5/384*q_k_be_s2_qp*s2^4/(E_0_phi*I_be);
    w_be_s1_qp=(5/384*q_k_be_s1_qp*s1+1/48*Q_k_be_s1_qp)*s1^3/(E_0_phi*I_be);
    w_be_s2_lim_qp=s2/0.300;
    w_be_s1_lim_qp=s1/0.300;
end

%Display Variables
%beamsvar_names2 = {'E_0_m' 'N/mm^2';'phi' '-';'E_0_phi' 'N/mm^2';'b_be' 'm';'h_be' 'm';'I_be'
'm^4';'g_0k_be' 'N/mm^2';'g_Ak_be' 'N/mm^2';'q_Nk_S' 'N/mm^2';'q_Nk_resid'
'N/mm^2';'q_Nk_office' 'N/mm^2';'psi_1' '-';'psi_2' '-';'q_k_be_s1_rare' 'kN/m';'q_k_be_s2_rare'
'kN/m';'q_k_be_s1_fr' 'kN/m';'q_k_be_s2_fr' 'kN/m';'q_k_be_s1_qp' 'kN/m';'q_k_be_s2_qp'
'kN/m';'w_be_s1_rare' 'mm   (must be < w_be_s1_lim_rare)';'w_be_s2_rare' 'mm   (must be <
w_be_s2_lim_rare)';'w_be_s1_lim_rare' 'mm';'w_be_s2_lim_rare' 'mm';'w_be_s1_fr' 'mm   (must be
< w_be_s1_lim_fr)';'w_be_s2_fr' 'mm   (must be < w_be_s2_lim_fr)';'w_be_s1_lim_fr'
'mm';'w_be_s2_lim_fr' 'mm';'w_be_s1_qp' 'mm   (must be < w_be_s1_lim_qp)';'w_be_s2_qp' 'mm
(must be < w_be_s1_lim_qp)';'w_be_s1_lim_qp' 'mm';'w_be_s2_lim_qp' 'mm'};
%beamsvar_values2 =
[E_0_m,phi,E_0_phi,b_be,h_be,I_be,g_0k_be,g_Ak_be,q_Nk_S,q_Nk_resid,q_Nk_office,psi_1,psi
_2,q_k_be_s1_rare,q_k_be_s2_rare,q_k_be_s1_fr,q_k_be_s2_fr,q_k_be_s1_qp,q_k_be_s2_qp,w
_be_s1_rare,w_be_s2_rare,w_be_s1_lim_rare,w_be_s2_lim_rare,w_be_s1_fr,w_be_s2_fr,w_be_s
1_lim_fr,w_be_s2_lim_fr,w_be_s1_qp,w_be_s2_qp,w_be_s1_lim_qp,w_be_s2_lim_qp];

%disp(char({'';'';'Final Deflection Values Beams';''}))

%for i = 1:size(beamsvar_names2,1)
    %disp([char(beamsvar_names2(i,1)),' = ', num2str(beamsvar_values2(i)),'  ',
char(beamsvar_names2(i,2))])
%end
```

```
%disp(char(' '))
%disp([char('b_be'),' = ', num2str(b_be),'  ', char('m')])
%disp([char('h_be'),' = ', num2str(h_be),'  ', char('m')])
%disp(char(' '))

%Volumes
%1 Story
nrbeams_s1=min(L,B)/s1*(max(L,B)/s2+1);
nrbeams_s2=max(L,B)/s2*(min(L,B)/(s1/2)+1);%1 every half span of columns.
%n Stories
Nrbeams_s1=nrbeams_s1*n_st_o;
Nrbeams_s2=nrbeams_s2*n_st_o;

volumebeams_s1=b_be*h_be*Nrbeams_s1*s1;
volumebeams_s2=b_be*h_be*Nrbeams_s2*s2;
volumebeams_tot(n_st_o)=volumebeams_s1+volumebeams_s2;

%Display Volumes
%volumebeamsvar_names = {'nrbeams_s1' 'each story';'nrbeams_s2' 'each story';'Nrbeams_s1'
'over whole building';'Nrbeams_s2' 'over whole building';'Volume beams s1' 'm^3';'Volume beams
s2' 'm^3';'Total Volume beams' 'm^3'};
%volumebeamsvar_values =
[nrbeams_s1,nrbeams_s2,Nrbeams_s1,Nrbeams_s2,volumebeams_s1,volumebeams_s2,volumeb
eams_tot(n_st_o)];

%disp(char({'';'Total Nr. and Volume Beams';''}))

%for i = 1:size(volumebeamsvar_names,1)
   %disp([char(volumebeamsvar_names(i,1)),' = ', num2str(volumebeamsvar_values(i)),'  ',
char(volumebeamsvar_names(i,2))])
%end
%disp(char(' '))
%% Columns
%Material Properties
sortofmaterialcolumn='glulam';%solid or glulam wood, or glued bamboo (glubam)
strengthclasscolumn='C24';%important only for timber
if strcmp(sortofmaterialcolumn,'glubam Bamboo')%Bamboo
   f_c_0_d=63;
   %f_c_90_d=f_c_0_d/10;%assumption of factor 10 for engineered bamboo
elseif strcmp(sortofmaterialcolumn,'glulam')%Glulam GL24h
   f_c_0_d=14.5;%k_mod=1, humidity class 1
   %f_c_90_d=1.9;%this value is determinant, as the columns can buckle in both directions
else%Solid C24
   f_c_0_d=12;
end

%Profile
h_co=0.01;%ev. minimal profile height
b_co=h_co;%quadratic profils
L_co=3.2;%3.2m approximately of static length of columns, one part is in the beam fixment
A_co(n_st_o)=b_co*h_co;
V_co(n_st_o)=A_co(n_st_o)*L_co;%for the loads
I_y_co=b_co*h_co^3/12;
I_z_co=h_co*b_co^3/12;
i_y_co=sqrt(I_y_co/A_co(n_st_o));
i_z_co=sqrt(I_z_co/A_co(n_st_o));
```

```
%Loads (own Weight could be neglected for static)
g_0k_co=5;%5kN/m^3
g_Ak_co=1;%Assumption 100kg/m^2
N_k_co=(n_st_o-
1)*(q_k_be_s2*s2/4+q_k_be_s1*s1/2+Q_k_be_s1/2)+sum(V_co(1:n_st_o))*g_0k_co+B*L*q_Nk_S
/4;%extern beams have only half total load, snow load applies on all 4 columns.
N_d_co(n_st_o)=1.35*((n_st_o-
1)*(q_d_be_s2*s2/4+q_d_be_s1*s1/2+Q_d_be_s1/2)+sum(V_co(1:n_st_o))*g_0k_co)+1.5*B*L*q_
Nk_S/4;

%Compression & Buckling (SIA 265,(10)/(29))
bucklingfactor_y=1.0;%Pin supported
bucklingfactor_z=1.0;
L_k_y_co=bucklingfactor_y*L_co;
L_k_z_co=bucklingfactor_z*L_co;
lambda_y=L_k_y_co/i_y_co;
lambda_z=L_k_z_co/i_z_co;
if strcmp(sortofmaterialcolumn,'solid')
   lambda_rel_y=lambda_y/(18*pi);
   lambda_rel_z=lambda_z/(18*pi);
   betha_c=0.2;
else%engineered materials
   lambda_rel_y=lambda_y/(20*pi);
   lambda_rel_z=lambda_z/(20*pi);
   betha_c=0.1;
end
if (lambda_rel_y < 0.3) || (lambda_rel_z < 0.3) %control of minimal Slenderness
   buckling='lambda_rel < 0.3 -> no buckling';
else
   buckling='buckling to proof ';
end
k_y=0.5*(1+betha_c*(lambda_rel_y-0.3)+lambda_rel_y^2);
k_z=0.5*(1+betha_c*(lambda_rel_z-0.3)+lambda_rel_z^2);
k_c_y=1/(k_y+sqrt(k_y^2-lambda_rel_y^2));
if k_c_y > 1
   disp(char(''))
   disp(char('k_c_y > 1 -> no buckling'));
   break;
end
k_c_z=1/(k_z+sqrt(k_z^2-lambda_rel_z^2));
if k_c_z > 1
   disp(char(''))
   disp(char('k_c_z > 1 -> no buckling'));
   break;
end
sigma_c_0_k=N_k_co/A_co(n_st_o)/1000;%brought to N/mm^2
%sigma_c_90_k=sigma_c_0_k;
sigma_c_0_d=N_d_co(n_st_o)/A_co(n_st_o)/1000;%brought to N/mm^2
%sigma_c_90_d=sigma_c_0_d;

% %Display Variables
% columnsvar_names = {'f_c_0_d' 'N/mm^2';'b_co' 'm';'h_co' 'm';'L_co' 'm';'A_co' 'm^2';'V_co'
'm^3';'I_y_co' 'm^4';'I_z_co' 'm^4';'i_y_co' 'm';'i_z_co' 'm';'g_0k_co' 'kN/m^3';'g_Ak_co'
'kN/m^2';'N_k_co' 'kN';'N_d_co' 'kN';'bucklingfactor_y' '-';'bucklingfactor_z' '-';'L_k_y_co'
'm';'L_k_z_co' 'm';'lambda_y' '-';'lambda_z' '-';'lambda_rel_y' '-';'lambda_rel_z' '-';'betha_c=0.2' '-
```

```
';'k_y' '-';'k_z' '-';'k_c_y' '-';'k_c_z' '-';'sigma_c_0_k' 'N/mm^2   (must be <
k_c*f_c_0_d)';'sigma_c_0_d' 'N/mm^2   (must be < f_c_0_d)';'f_c_0_d' 'N/mm^2';'k_c_y*f_c_0_d'
'N/mm^2';'k_c_z*f_c_0_d' 'N/mm^2'};
% columnsvar_values =
[f_c_0_d,b_co,h_co,L_co,A_co(n_st_o),V_co(n_st_o),I_y_co,I_z_co,i_y_co,i_z_co,g_0k_co,g_Ak_
co,N_k_co,N_d_co,bucklingfactor_y,bucklingfactor_z,L_k_y_co,L_k_z_co,lambda_y,lambda_z,lam
bda_rel_y,lambda_rel_z,betha_c,k_y,k_z,k_c_y,k_c_z,sigma_c_0_k,sigma_c_0_d,f_c_0_d,k_c_y*f
_c_0_d,k_c_z*f_c_0_d];
%
% disp(char(' '))
% disp(char(' '))
% disp('Columns verification')
% disp(['Material:',' ',char(sortofmaterialcolumn)])
% disp(['Strength Class:',' ',char(strengthclasscolumn)])
% disp(char({'';'';'Start Values Columns';''}))
%
% for i = 1:size(columnsvar_names,1)
%     disp([char(columnsvar_names(i,1)),' = ', num2str(columnsvar_values(i)),'  ',
char(columnsvar_names(i,2))])
% end
% disp(char(' '))
% disp(char(buckling))
% disp(char(' '))
% disp([char('b_co'),' = ', num2str(b_co),'  ', char('m')])
% disp([char('h_co'),' = ', num2str(h_co),'  ', char('m')])
% disp(char(' '))

while (sigma_c_0_d > k_c_y*f_c_0_d) || (sigma_c_0_d > k_c_z*f_c_0_d)

    h_co = h_co + 0.05;
    b_co=h_co;%quadratic profils
    L_co=3.2;%3.2m approximately of static length of columns
    A_co(n_st_o)=b_co*h_co;
    V_co(n_st_o)=A_co(n_st_o)*L_co;
    I_y_co=b_co*h_co^3/12;
    I_z_co=h_co*b_co^3/12;
    i_y_co=sqrt(I_y_co/A_co(n_st_o));
    i_z_co=sqrt(I_z_co/A_co(n_st_o));

    %Loads (own Weight could be neglected for static)
    g_0k_co=5;%5kN/m^3
    g_Ak_co=1;%Assumption 100kg/m^2
    N_k_co=(n_st_o-
1)*(q_k_be_s2*s2/4+q_k_be_s1*s1/2+Q_k_be_s1/2)+sum(V_co(1:n_st_o))*g_0k_co+B*L*q_Nk_S
/4;%extern beams have only half total load, snow load applies on all 4 columns.
    N_d_co(n_st_o)=1.35*((n_st_o-
1)*(q_d_be_s2*s2/4+q_d_be_s1*s1/2+Q_d_be_s1/2)+sum(V_co(1:n_st_o))*g_0k_co)+1.5*B*L*q_
Nk_S/4;

    %Compression & Buckling (SIA 265,(10)/(29))
    bucklingfactor_y=1.0;%Pin supported
    bucklingfactor_z=1.0;
    L_k_y_co=bucklingfactor_y*L_co;
    L_k_z_co=bucklingfactor_z*L_co;
    lambda_y=L_k_y_co/i_y_co;
    lambda_z=L_k_z_co/i_z_co;
```

```
        if strcmp(sortofmaterialcolumn,'solid')
            lambda_rel_y=lambda_y/(18*pi);
            lambda_rel_z=lambda_z/(18*pi);
            betha_c=0.2;
        else%engineered materials
            lambda_rel_y=lambda_y/(20*pi);
            lambda_rel_z=lambda_z/(20*pi);
            betha_c=0.1;
        end
        if (lambda_rel_y < 0.3) || (lambda_rel_z < 0.3) %control of minimal Slenderness
            buckling='lambda_rel < 0.3 -> no buckling';
        else
            buckling='buckling to proof ';
        end
        k_y=0.5*(1+betha_c*(lambda_rel_y-0.3)+lambda_rel_y^2);
        k_z=0.5*(1+betha_c*(lambda_rel_z-0.3)+lambda_rel_z^2);
        k_c_y=1/(k_y+sqrt(k_y^2-lambda_rel_y^2));
        if k_c_y > 1
            disp(char(''))
            disp(char('k_c_y > 1 -> no buckling'));
            break;
        end
        k_c_z=1/(k_z+sqrt(k_z^2-lambda_rel_z^2));
        if k_c_z > 1
            disp(char(''))
            disp(char('k_c_z > 1 -> no buckling'));
            break;
        end
        sigma_c_0_k=N_k_co/A_co(n_st_o)/1000;%brought to N/mm^2
        %sigma_c_90_k=sigma_c_0_k;
        sigma_c_0_d=N_d_co(n_st_o)/A_co(n_st_o)/1000;%brought to N/mm^2
        %sigma_c_90_d=sigma_c_0_d;
end

% %Display Variables
% columnsvar_names = {'f_c_0_d' 'N/mm^2';'b_co' 'm';'h_co' 'm';'L_co' 'm';'A_co' 'm^2';'V_co'
'm^3';'I_y_co' 'm^4';'I_z_co' 'm^4';'i_y_co' 'm';'i_z_co' 'm';'g_0k_co' 'kN/m^3';'g_Ak_co'
'kN/m^2';'N_k_co' 'kN';'N_d_co' 'kN';'bucklingfactor_y' '-';'bucklingfactor_z' '-';'L_k_y_co'
'm';'L_k_z_co' 'm';'lambda_y' '-';'lambda_z' '-';'lambda_rel_y' '-';'lambda_rel_z' '-';'betha_c=0.2' '-
';'k_y' '-';'k_z' '-';'k_c_y' '-';'k_c_z' '-';'sigma_c_0_k' 'N/mm^2   (must be <
k_c*f_c_0_d)';'sigma_c_0_d' 'N/mm^2   (must be < f_c_0_d)';'f_c_0_d' 'N/mm^2';'k_c_y*f_c_0_d'
'N/mm^2';'k_c_z*f_c_0_d' 'N/mm^2'};
% columnsvar_values =
[f_c_0_d,b_co,h_co,L_co,A_co(n_st_o),V_co(n_st_o),I_y_co,I_z_co,i_y_co,i_z_co,g_0k_co,g_Ak_
co,N_k_co,N_d_co,bucklingfactor_y,bucklingfactor_z,L_k_y_co,L_k_z_co,lambda_y,lambda_z,lam
bda_rel_y,lambda_rel_z,betha_c,k_y,k_z,k_c_y,k_c_z,sigma_c_0_k,sigma_c_0_d,f_c_0_d,k_c_y*f
_c_0_d,k_c_z*f_c_0_d];
%
%
% disp(char({'';'';'Final Values Columns';''}))
%
% for i = 1:size(columnsvar_names,1)
%     disp([char(columnsvar_names(i,1)),' = ', num2str(columnsvar_values(i)),'  ',
char(columnsvar_names(i,2))])
% end
% disp(char(' '))
```

```
% disp(char(buckling))
% disp(char(' '))
% disp([char('b_co'),' = ', num2str(b_co),'  ', char('m')])
% disp([char('h_co'),' = ', num2str(h_co),'  ', char('m')])
% disp(char(' '))

%Volumes
%Extern walls and structural intern walls too.
%Intern walls assumed to be spaced with s1
%1 Story
nrcolumns=(min(L,B)/s1+1)*(max(L,B)/s2+1);%4 columns
%n Stories
Nrcolumns=nrcolumns*n_st_o;
%1 Story
volumecolumns(n_st_o)=b_co*h_co*H_st*nrcolumns;%geometrical height column is intern storey
height
volumediagonal(n_st_o)=b_co*h_co*H_st;%1 diagonal for wind loads
volumevertical(n_st_o)=volumecolumns(n_st_o)+volumediagonal(n_st_o);
%n Stories
volumecolumns_tot(n_st_o)=sum(volumecolumns(1:n_st_o));
volumediagonal_tot(n_st_o)=sum(volumediagonal(1:n_st_o));
volumevertical_tot(n_st_o)=volumecolumns_tot(n_st_o)+volumediagonal_tot(n_st_o);

% % Display Volumes
% volumecolumnsvar_names = {'nrcolumns' 'each story';'Nr. columns' 'over whole building';'Total
Volume vertical' 'm^3'};
% volumecolumnsvar_values = [nrcolumns,Nrcolumns,volumevertical_tot(n_st_o)];
%
% disp(char({'';'Total Nr. and Volume of Columns';''}))
%
% for i = 1:size(volumecolumnsvar_names,1)
%     disp([char(volumecolumnsvar_names(i,1)),' = ', num2str(volumecolumnsvar_values(i)),'  ',
char(volumecolumnsvar_names(i,2))])
% end
% disp(char(' '))
%% Foundation
%Material Properties
%Ground
sigma_bd=700;%in kN/m^2;for C_c = 30%, compression ground
%Concrete
sortofmaterialfoundation='concrete';
strengthclassfoundation='C25/30';
if strcmp(strengthclassfoundation,'C25/30')
    f_cd=16.5;%connect this value to the material with if function.
else%C30/37
    f_cd=20;
end

%Profile
%b_co and h_co of cross-section known
P_found=0.03;%armature content
t_plate_concrete=0.1;%10cm of concrete for closing of flat foundation for isolation
t_found=t_plate_concrete;%total thickness under columns
s_found=h_co+2*t_found;%Force propagation 1:1
f_found=s_found;%width of local foundation, f_found > s_found
```

```
%Compression
%Ground
sigma_b_d=N_d_co(n_st_o)/(f_found*f_found);%in kN/m^2, concentrated foundation for fxf
%sigma_bd must be greater than sigma_b_d
%this should get the help of the plate compression, else the foundation has
%to get ways to big

%Concrete
%sigma_c_d=N_d_co/A_co/1000; must be smaller sigma_c_Rd
%SIA262,4.2.1.10 with 1:2 compression propagation resistence
k_c_found(n_st_o)=1/sqrt((h_co*b_co)/((h_co+t_found)*(b_co+t_found)));
if k_c_found(n_st_o) > 3
   disp(char(''))
   disp(char('k_c_found > 3 (SIA 262,(32))'));%if k_c_found > 3, the compression of the concrete is too high -> either bigger foundation or more columns, for less compression
   break;
end
sigma_c_Rd=k_c_found(n_st_o)*f_cd;%k_c_found contains distribution of force

%Display Variables
%foundationvar_names = {'sigma_bd' 'kN/m^2';'f_cd' 'N/mm^2'; 'b_co' 'm'; 'h_co' 'm';'A_co' 'm^2';'t_plate_concrete' 'm';'t_found' 'm';'s_found' 'm';'f_found' 'm';'N_d_co' 'kN'; 'sigma_b_d' 'kN/m` (must be < sigma_bd)'; 'sigma_bd' 'kN/m`';'k_c_found' '-'; 'sigma_c_d' 'N/mm^2   (must be < sigma_c_Rd)';'sigma_c_Rd' 'N/mm^2'};
%foundationvar_values = [sigma_bd,f_cd,b_co,h_co,A_co(n_st_o),t_plate_concrete,t_found,s_found,f_found,N_d_co,sigma_b_d,sigma_bd,k_c_found,sigma_c_d,sigma_c_Rd];

%disp(char(' '))
%disp(char(' '))
%disp('Foundation verification')
%disp(['Material:',' ','Concrete'])
%disp(['Strength Class:',' ',char(strengthclassfoundation)])
%disp(char({'';'';'Start Values Foundation';''}))

%for i = 1:size(foundationvar_names,1)
   %disp([char(foundationvar_names(i,1)),' = ', num2str(foundationvar_values(i)),'  ', char(foundationvar_names(i,2))])
%end
%disp(char(' '))
%disp([char('h_co'),' = ', num2str(h_co),'  ', char('m')])
%disp([char('b_co'),' = ', num2str(b_co),'  ', char('m')])
%disp([char('t_plate_concrete'),' = ', num2str(t_plate_concrete),'  ', char('m')])
%disp([char('t_found'),' = ', num2str(t_found),'  ', char('m')])
%disp([char('s_found'),' = ', num2str(s_found),'  ', char('m')])
%disp([char('f_found'),' = ', num2str(f_found),'  ', char('m')])
%disp([char('Armature content'),' = ', num2str(P_found),'  ', char('-')])
%disp(char(' '))

%Micropiles eventually
l_micropiles=0.5*H;%approximation of half of building height
d_micropiles=0.100;
t_micropiles=0.010;
nr_micropiles=0;
N_Rd_micropiles=l_micropiles*d_micropiles*pi*sigma_bd*nr_micropiles;
```

```
while sigma_c_0_d > sigma_c_Rd || sigma_b_d > sigma_bd
    if (t_found < 0.6) && (f_found < 2.0)
        %Profile
        %b_co and h_co of cross-section known
        t_found=t_found + 0.01;%total thickness under columns
        s_found=h_co+2*t_found;%Force propagation 1:1
        f_found=s_found;%width of local foundation, f_found > s_found

        %Compression
        %Ground
        sigma_b_d=N_d_co(n_st_o)/(f_found*f_found);%in kN/m^2, concentrated foundation for fxf
        %sigma_bd must be greater than sigma_b_d
        %this should get the help of the plate compression, else the foundation has
        %to get ways to big

        %Concrete
        %sigma_c_d=N_d_co/A_co/1000; must be smaller sigma_c_Rd
        %SIA262,4.2.1.10 with 1:2 compression propagation resistence
        k_c_found(n_st_o)=1/sqrt((h_co*b_co)/((h_co+t_found)*(b_co+t_found)));
        if k_c_found(n_st_o) > 3
            disp(char(''))
            disp(char('k_c_found > 3 (SIA 262,(32))'));%if k_c_found > 3, the compression of the
concrete is too high -> either bigger foundation or more columns, for less compression
            break;
        end
        sigma_c_Rd=k_c_found(n_st_o)*f_cd;%k_c_found contains distribution of force
    else
        %Profile
        %b_co and h_co of cross-section known
        t_found=min(0.6,(2-h_co)/2);%t_founs so, that s_found doesn't get bigger than 2m
        s_found=h_co+2*t_found;%Force propagation 1:1
        f_found=2;%2m x 2m foundation foot enabling enough place for micropiles

        %Compression
        %Ground
        nr_micropiles=max(nr_micropiles+1,4);%4 minimal amount of micropiles because of the high
costs of work
        N_Rd_micropiles=l_micropiles*d_micropiles*pi*sigma_bd*0.1*nr_micropiles;%tau = 10% of
sigma
        N_d_b_d=max(0,N_d_co(n_st_o) - N_Rd_micropiles);
        sigma_b_d=N_d_b_d/(f_found*f_found);%in kN/m^2, concentrated foundation for fxf
        %sigma_bd must be greater than sigma_b_d
        %this should get the help of the plate compression, else the foundation has
        %to get ways to big

        %Concrete
        %sigma_c_d=N_d_co/A_co/1000; must be smaller sigma_c_Rd
        %SIA262,4.2.1.10 with 1:2 compression propagation resistence
        k_c_found(n_st_o)=1/sqrt((h_co*b_co)/((h_co+t_found)*(b_co+t_found)));
        if k_c_found(n_st_o) > 3
            disp(char(''))
            disp(char('k_c_found > 3 (SIA 262,(32))'));%if k_c_found > 3, the compression of the
concrete is too high -> either bigger foundation or more columns, for less compression
            break;
        end
        sigma_c_Rd=k_c_found(n_st_o)*f_cd;%k_c_found contains distribution of force
```

```
   end
end
nr_micropiles_tot(n_st_o)=nr_micropiles*4;%4columns

%Display Variables
%foundationvar_names = {'sigma_bd' 'kN/m^2';'f_cd' 'N/mm^2'; 'b_co' 'm'; 'h_co' 'm';'A_co'
'm^2';'t_plate_concrete' 'm';'t_found' 'm';'s_found' 'm';'f_found' 'm';'N_d_co' 'kN'; 'sigma_b_d' 'kN/m`
(must be < sigma_bd)'; 'sigma_bd' 'kN/m`';'k_c_found' '-'; 'sigma_c_d' 'N/mm^2   (must be <
sigma_c_Rd)';'sigma_c_Rd' 'N/mm^2'};
%foundationvar_values =
[sigma_bd,f_cd,b_co,h_co,A_co(n_st_o),t_plate_concrete,t_found,s_found,f_found,N_d_co,sigma
_b_d,sigma_bd,k_c_found,sigma_c_d,sigma_c_Rd];

%disp(char({'';'';'Final Values Foundation';''}))

%for i = 1:size(foundationvar_names,1)
   %disp([char(foundationvar_names(i,1)),' = ', num2str(foundationvar_values(i)),'  ',
char(foundationvar_names(i,2))])
%end
%disp(char(' '))
%disp([char('h_co'),' = ', num2str(h_co),'  ', char('m')])
%disp([char('t_plate_concrete'),' = ', num2str(t_plate_concrete),'  ', char('m')])
%disp([char('t_found'),' = ', num2str(t_found),'  ', char('m')])
%disp([char('s_found'),' = ', num2str(s_found),'  ', char('m')])
%disp([char('f_found'),' = ', num2str(f_found),'  ', char('m')])
%disp([char('Armature content'),' = ', num2str(P_found),'  ', char('-')])
%disp(char(' '))


%Volumes
volumeplate=t_plate_concrete*(B+2*(s_found/2-h_co/2))*(L+2*(s_found/2-h_co/2));
volumeconcentratedfoundation=(t_found-t_plate_concrete)*f_found*f_found*nrcolumns;%under
columns
volumefoundation_concrete(n_st_o)=volumeplate + volumeconcentratedfoundation;
volumefoundation_armature(n_st_o)=volumefoundation_concrete(n_st_o)*P_found;
volumemicropiles(n_st_o)=l_micropiles*(d_micropiles^2-
t_micropiles^2)*pi/4*nr_micropiles_tot(n_st_o);

%Display Volumes
%disp([char('Volume plate'),' = ', num2str(volumeplate),'  ', char('m^3')])
%disp([char('Volume concentrated foundation'),' = ', num2str(volumeconcentratedfoundation),'  ',
char('m^3')])
%disp([char('Volume ribbon foundation'),' = ', num2str(volumeribbonfoundation),'  ', char('m^3')])
%disp([char('Tot. Volume foundation'),' = ', num2str(volumefoundation_concrete(n_st_o)),'  ',
char('m^3')])
%disp([char('Tot. Volume armature'),' = ', num2str(volumefoundation_armature(n_st_o)),'  ',
char('m^3')])
%disp(char(' '))
%% Summary
disp(char(' '))
disp(char(' '))
disp(char('Summary of Values'))
disp(char(' '))
disp(char('SCHEME 4 : Post and beams timber or bamboo frame with diagonal bracing and CLT or
CLB floor panels'))
disp([char('Nr. stories'),' = ', num2str(n_st_o)])
```

```
disp(char(' '))
disp(char('Slabs'))
if strcmp(sortofmaterialslab,'CLB')
   disp([char('Material'),': ', num2str(sortofmaterialslab)])
else
   disp([char('Material'),': ', num2str(sortofmaterialslab),', ', num2str(strengthclassslab)])
end
slab_names = {'b' 'm';'h_sl' 'm';'Total Volume' 'm^3'};
slab_values = [b,h_sl,volumeslabs(n_st_o)];
% for i = 1:size(slab_values,2)
%     disp([char(slab_names(i,1)),' = ', num2str(slab_values(i)),'  ', char(slab_names(i,2))])
% end
for i = 1:size(slab_values,2)%only values output
   disp(num2str(slab_values(i)))
end
disp([char('Max exploit'),': ', char(maxexploitslabs_name),' = ', num2str(maxexploitslabs_value)])
disp(char(' '))
disp(char('Beams'))
if strcmp(sortofmaterialbeam,'glubam Bamboo')
   disp([char('Material'),': ', num2str(sortofmaterialbeam)])
else
   disp([char('Material'),': ', num2str(sortofmaterialbeam),', ', num2str(strengthclassbeam)])
end
beams_names = {'b_be' 'm';'h_be' 'm';'Total Volume' 'm^3'};
beams_values = [b_be,h_be,volumebeams_tot(n_st_o)];
% for i = 1:size(beams_values,2)
%     disp([char(beams_names(i,1)),' = ', num2str(beams_values(i)),'  ', char(beams_names(i,2))])
% end
for i = 1:size(beams_values,2)%only values output
   disp(num2str(beams_values(i)))
end
disp(char(' '))
disp(char('Columns & Wind diagonal'))
if strcmp(sortofmaterialcolumn,'glubam Bamboo')
   disp([char('Material'),': ', num2str(sortofmaterialcolumn)])
else
   disp([char('Material'),': ', num2str(sortofmaterialcolumn),', ', num2str(strengthclasscolumn)])
end
columns_names = {'h_co' 'm';'b_co' 'm';'Total Volume' 'm^3'};
columns_values = [h_co,b_co,volumevertical_tot(n_st_o)];
% for i = 1:size(columns_values,2)
%     disp([char(columns_names(i,1)),' = ', num2str(columns_values(i)),'  ',
char(columns_names(i,2))])
% end
for i = 1:size(columns_values,2)%only values output
   disp(num2str(columns_values(i)))
end
disp(char(' '))
disp(char(' '))
disp(char(' '))
disp(char('Foundation'))
disp([char('Material'),': ', 'Concrete' ,', ', num2str(strengthclassfoundation)])
foundation_names = {'t_plate_concrete' 'm';'t_found' 'm';'s_found' 'm';'f_found' 'm'; 'Armature
content' '-'; 'Volume plate' 'm^3';'Volume concentrated foundation' 'm^3';'Volume ribbon foundation'
'm^3';'Tot. Volume armature' 'm^3'; 'Tot Number Micropiles (steel)' 'm^3';'Tot. Volume Micropiles
(steel)' 'm^3'};
```

```
foundation_values =
[t_plate_concrete,t_found,s_found,f_found,P_found,volumeplate,volumeconcentratedfoundation,vo
lumefoundation_concrete(n_st_o),volumefoundation_armature(n_st_o),nr_micropiles_tot(n_st_o),v
olumemicropiles(n_st_o)];
% for i = 1:size(foundation_values,2)
%     disp([char(foundation_names(i,1)),' = ', num2str(foundation_values(i)),'  ',
char(foundation_names(i,2))])
% end
for i = 1:size(foundation_values,2)%only values output
   disp(num2str(foundation_values(i)))
end
disp(char(' '))
disp(char(' '))

disp(char('TOTAL Materials'))
volume_Biobased_beamsandverticals_tot(n_st_o)=volumebeams_tot(n_st_o)+volumevertical_tot(
n_st_o);
% if strcmp(sortofmaterialslab,'CLB')
%     disp([char('Tot. Volume CLB in building'),' = ', num2str(volumeslabs(n_st_o)),'  ', char('m^3')])
% else
%     disp([char('Tot. Volume CLT in building'),' = ', num2str(volumeslabs(n_st_o)),'  ', char('m^3')])
% end
% if strcmp(sortofmaterialbeam,'glubam Bamboo')
%     disp([char('Tot. Volume Glubam Bamboo in building'),' = ',
num2str(volume_Biobased_beamsandverticals_tot(n_st_o)),'  ', char('m^3')])
% else
%     disp([char('Tot. Volume Glulam in building'),' = ',
num2str(volume_Biobased_beamsandverticals_tot(n_st_o)),'  ', char('m^3')])
% end
TOTAL_names = {'Tot. Volume Biobased slabs' 'm^3';'Tot. Volume Biobased beams and verticals'
'm^3';'Tot. Volume Concrete C25/30' 'm^3';'Tot. Volume Armature' 'm^3';'Tot. Volume Micropiles
(steel)' 'm^3'};
TOTAL_values =
[volumeslabs(n_st_o),volume_Biobased_beamsandverticals_tot(n_st_o),volumefoundation_concre
te(n_st_o),volumefoundation_armature(n_st_o),volumemicropiles(n_st_o)];
% for i = 1:size(TOTAL_values,2)
%     disp([char(TOTAL_names(i,1)),' = ', num2str(TOTAL_values(i)),'  ', char(TOTAL_names(i,2))])
% end
for i = 1:size(TOTAL_values,2)%only values output
   disp(num2str(TOTAL_values(i)))
end
disp(char(' '))
usageratio_slab_tot(n_st_o)=volumeslabs(n_st_o)/(B*L*n_st_o);
usageratio_Biobased_beamsandverticals_tot(n_st_o)=volume_Biobased_beamsandverticals_tot(n
_st_o)/(B*L*n_st_o);
usageratio_concrete_tot(n_st_o)=volumefoundation_concrete(n_st_o)/(B*L*n_st_o);
usageratio_armature_tot(n_st_o)=volumefoundation_armature(n_st_o)/(B*L*n_st_o);
usageratio_micropiles(n_st_o)=volumemicropiles(n_st_o)/(B*L*n_st_o);
% if strcmp(sortofmaterialslab,'CLB')
%     disp([char('Usage ratio CLB'),' = ', num2str(usageratio_slab_tot(n_st_o)),'  ', char('m^3/m^2
RES')])
% else
%     disp([char('Usage ratio CLT'),' = ', num2str(usageratio_slab_tot(n_st_o)),'  ', char('m^3/m^2
RES')])
% end
% if strcmp(sortofmaterialbeam,'glubam Bamboo')
```

```
%     disp([char('Usage ratio Glubam Bamboo'),' = ',
num2str(usageratio_Biobased_beamsandverticals_tot(n_st_o)),'  ', char('m^3/m^2 RES')])
% else
%     disp([char('Usage ratio Glulam'),' = ',
num2str(usageratio_Biobased_beamsandverticals_tot(n_st_o)),'  ', char('m^3/m^2 RES')])
% end
usage_names = {'Usage ratio Biobased slabs' 'm^3/m^2 RES';'Usage ratio Biobased beams and
verticals' 'm^3/m^2 RES';'Usage ratio Concrete C25/30' 'm^3/m^2 RES';'Usage ratio Armature'
'm^3/m^2 RES';'Usage ratio Micropiles (steel)' 'm^3/m^2 RES'};
usage_values =
[usageratio_slab_tot(n_st_o),usageratio_Biobased_beamsandverticals_tot(n_st_o),usageratio_con
crete_tot(n_st_o),usageratio_armature_tot(n_st_o),usageratio_micropiles(n_st_o)];
% for i = 1:size(usage_values,2)
%     disp([char(usage_names(i,1)),' = ', num2str(usage_values(i)),'  ', char(usage_names(i,2))])
% end
for i = 1:size(usage_values,2)%only values output
   disp(num2str(usage_values(i)))
end

if strcmp(sortofmaterialslab,'CLB')
   usageratio_impact_slab(n_st_o)=167.47*usageratio_slab_tot(n_st_o);%CLB
else
   usageratio_impact_slab(n_st_o)=495.09*usageratio_slab_tot(n_st_o);%CLT
end
if strcmp(sortofmaterialcolumn,'glubam Bamboo')%assumption columns and beams same material

usageratio_impact_Biobased_beamsandverticals(n_st_o)=273.17*usageratio_Biobased_beamsan
dverticals_tot(n_st_o);%glubam Bamboo
else

usageratio_impact_Biobased_beamsandverticals(n_st_o)=101.21*usageratio_Biobased_beamsan
dverticals_tot(n_st_o);%glulam
end
usageratio_impact_concrete(n_st_o)=170.2*usageratio_concrete_tot(n_st_o);%C25/30
usageratio_impact_steel(n_st_o)=5353.7*(usageratio_armature_tot(n_st_o)+usageratio_micropiles
(n_st_o));
usageratio_impact_tot(n_st_o)=
usageratio_impact_slab(n_st_o)+usageratio_impact_Biobased_beamsandverticals(n_st_o)+usage
ratio_impact_concrete(n_st_o)+usageratio_impact_steel(n_st_o);

if strcmp(sortofmaterialslab,'CLB')
   diet=' bam ';
else
   diet=' tim ';
end
end
figure(41);
plot(1:maxstories,volumeslabs)
hold on;
plot(1:maxstories,volumebeams_tot)
plot(1:maxstories,volumecolumns_tot)
plot(1:maxstories,volumediagonal_tot)
plot(1:maxstories,volume_Biobased_beamsandverticals_tot)
plot(1:maxstories,volumefoundation_concrete)
plot(1:maxstories,volumefoundation_armature)
plot(1:maxstories,volumemicropiles)
```

```
legend(['slabs',' ', num2str(sortofmaterialslab)],['beams',' ',
num2str(sortofmaterialbeam)],['columns',' ', num2str(sortofmaterialcolumn)],['diagonal',' ',
num2str(sortofmaterialbeam)],['TOTAL',' ', num2str(sortofmaterialbeam)],'foundation Concrete
C25/30','foundation Armature','Volume Micropiles (steel)')
xlabel('number of stories')
ylabel('volume [m^3]')
xlim([1 14])
ylim([-100 300])
annotation('doublearrow',[0.13,0.31],[0.24,0.24])
annotation('textbox',[0.17,0.24,0.1,0],'string','SFH & TH')
annotation('doublearrow',[0.13,0.545],[0.2,0.2])
annotation('textbox',[0.29,0.2,0.05,0],'string','MFH')
annotation('doublearrow',[0.13,0.905],[0.16,0.16])
annotation('textbox',[0.47,0.16,0.04,0],'string','AB')
hold off;
saveas(gcf,['images/Matlab plots/41_',diet,'_totvolumes_all'],'epsc')
figure(42);
plot(1:maxstories,volumeslabs)
hold on;
plot(1:maxstories,volume_Biobased_beamsandverticals_tot)
plot(1:maxstories,volumefoundation_concrete)
plot(1:maxstories,volumefoundation_armature)
plot(1:maxstories,volumemicropiles)
legend(['slabs',' ', num2str(sortofmaterialslab)],['Total',' ', num2str(sortofmaterialbeam)],'foundation
Concrete C25/30','foundation Armature','Volume Micropiles (steel)')
xlabel('number of stories')
ylabel('volume [m^3]')
xlim([1 14])
ylim([-100 300])
annotation('doublearrow',[0.13,0.31],[0.24,0.24])
annotation('textbox',[0.17,0.24,0.1,0],'string','SFH & TH')
annotation('doublearrow',[0.13,0.545],[0.2,0.2])
annotation('textbox',[0.29,0.2,0.05,0],'string','MFH')
annotation('doublearrow',[0.13,0.905],[0.16,0.16])
annotation('textbox',[0.47,0.16,0.04,0],'string','AB')
hold off;
saveas(gcf,['images/Matlab plots/42_',diet,'_totvolumes_summary'],'epsc')
figure(43);
plot(1:maxstories,usageratio_slab_tot)
hold on;
plot(1:maxstories,usageratio_Biobased_beamsandverticals_tot)
plot(1:maxstories,usageratio_concrete_tot)
plot(1:maxstories,usageratio_armature_tot)
plot(1:maxstories,usageratio_micropiles)
legend(['Total ratio',' ', num2str(sortofmaterialslab)],['Total ratio',' ',
num2str(sortofmaterialbeam)],'ratio foundation Concrete C25/30','ratio foundation Armature','ratio
Micropiles (steel)')
xlabel('number of stories')
ylabel('ratio [m^3/m^2 RES]')
xlim([1 14])
ylim([-0.060 0.22])
annotation('doublearrow',[0.13,0.31],[0.24,0.24])
annotation('textbox',[0.17,0.24,0.1,0],'string','SFH & TH')
annotation('doublearrow',[0.13,0.545],[0.2,0.2])
annotation('textbox',[0.29,0.2,0.05,0],'string','MFH')
annotation('doublearrow',[0.13,0.905],[0.16,0.16])
```

```
annotation('textbox',[0.47,0.16,0.04,0],'string','AB')
hold off;
saveas(gcf,['images/Matlab plots/43_',diet,'_RES'],'epsc')
figure(44);
plot(1:maxstories,usageratio_impact_slab)
hold on;
plot(1:maxstories,usageratio_impact_Biobased_beamsandverticals)
plot(1:maxstories,usageratio_impact_concrete)
plot(1:maxstories,usageratio_impact_steel)
plot(1:maxstories,usageratio_impact_tot)
legend(['ratio',' ', num2str(sortofmaterialslab),' ', 'impact'],['ratio',' ', num2str(sortofmaterialbeam),' ',
'impact'],'ratio concrete impact','ratio steel impact','ratio total building impact')
xlabel('number of stories')
ylabel('ratio impact [kg CO_2 / m^2 RES]')
xlim([1 14])
ylim([-40 150])
annotation('doublearrow',[0.13,0.31],[0.24,0.24])
annotation('textbox',[0.17,0.24,0.1,0],'string','SFH & TH')
annotation('doublearrow',[0.13,0.545],[0.2,0.2])
annotation('textbox',[0.29,0.2,0.05,0],'string','MFH')
annotation('doublearrow',[0.13,0.905],[0.16,0.16])
annotation('textbox',[0.47,0.16,0.04,0],'string','AB')
hold off;
saveas(gcf,['images/Matlab plots/44_',diet,'_CO2impact'],'epsc')
%% ------------- END CODE --------------
```
